# The structural basis for LRRK2’s activation and autoinhibition

**DOI:** 10.1016/j.cell.2026.07.027

**Published:** 2026-08-10

**Authors:** Amalia Villagran Suarez, Kathryn S. Hatch, Tatyana Bodrug, Wei Gai, Katherine J. Surridge, Elizabeth Moussikhina, Kendrick H.V. Nguyen, Marta Sanz-Murillo, Robert Callahan, Erica Xiong, Delisa Ramos, Lawrence Zhu, Verena Dederer, Sebastian Mathea, Janet Iwasa, Stefan Knapp, Kevan M. Shokat, Samara L. Reck-Peterson, Andres E. Leschziner

**Affiliations:** 1 Department of Cellular and Molecular Medicine, University of California, San Diego, La Jolla, CA 92093, USA; 2 Department of Cellular and Molecular Pharmacology, University of California, San Francisco, San Francisco, CA 94143, USA; 3 Institute of Pharmaceutical Chemistry and Structural Genomics Consortium, Goethe-University Frankfurt am Main, Frankfurt, Germany; 4 Department of Biochemistry, University of Utah, Salt Lake City, UT 84132, USA; 5 Howard Hughes Medical Institute, Chevy Chase, MD 20815, USA; 6 Department of Cell and Developmental Biology, University of California, San Diego, La Jolla, CA 92093, USA; 7 Department of Molecular Biology, University of California, San Diego, La Jolla, CA 92093, USA; 8 LRRK2 Investigative Therapeutics Exchange (LITE), New York, NY 10120, USA; 9 Present address: Department of Biochemistry and Biophysics, Weill Cornell Medicine, New York, NY 10065, USA; 10 Present address: Spanish National Cancer Research Centre (CNIO), 28029 Madrid, Spain; 11 Present address: Penn Medical Scientist Training Program, Perelman School of Medicine, University of Pennsylvania, Philadelphia, PA 19104, USA; 12 Present address: Salk Institute for Biological Studies, La Jolla, CA 92037, USA; 13 Present address: Medical Scientist Training Program, University of California San Francisco, San Francisco, CA 94143, USA; 14 Present address: AaviGen GmbH, Heidelberg 69120, Germany; 15 These authors contributed equally; 16 These authors contributed equally; 17 Lead contact

## Abstract

Mutations in leucine-rich repeat kinase 2 (LRRK2) are the second most common cause of autosomal-dominant Parkinson’s disease (PD), and increased LRRK2 kinase activity is also observed in idiopathic PD, making LRRK2 a major actionable therapeutic target. LRRK2 is a 286-kDa multidomain enzyme containing a Ras-like GTPase (ROC) and a kinase domain. Using cryo-electron microscopy (cryo-EM), biochemical reconstitution, and cell-based assays, we show that the ROC GTPase governs switching between autoinhibited and active states: GTP binding promotes activation, whereas GDP binding enforces autoinhibition. Two common PD-linked mutations, G2019S and R1441C/G/H, activate LRRK2 through distinct structural mechanisms, revealing genotype-specific routes to dysregulation. These findings provide a unified framework for understanding LRRK2 regulation with broad therapeutic implications. Stabilizing the guanosine diphosphate (GDP)-bound state may inhibit LRRK2 by maintaining autoinhibition, whereas promoting the GTP-bound state could be advantageous in specific cellular contexts, such as the lung, where increased LRRK2 kinase activity may play protective or regulatory roles.

## In brief

Elevated kinase activity in leucine-rich repeat kinase 2 (LRRK2) is linked to Parkinson’s disease; this study shows that its ROC GTPase acts as a nucleotide-dependent molecular switch that regulates activation and autoinhibition and reveals how some but not all PD mutations disrupt this regulatory switch.

## Graphical Abstract

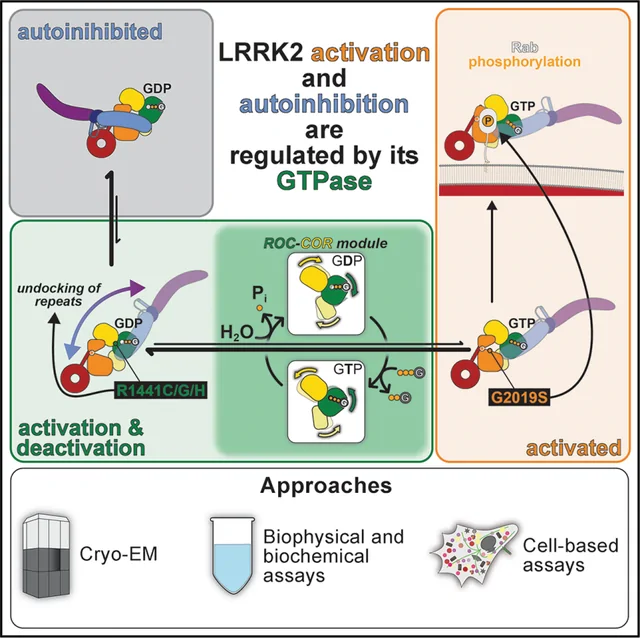

## INTRODUCTION

Mutations in leucine-rich repeat kinase 2 (LRRK2) are one of the main causes of familial Parkinson’s disease (PD).^1^ LRRK2 phosphorylates a subset of Rab GTPases associated with membrane vesicles, which includes those in the endolysosomal system and secretory pathway.^2–4^ PD-linked mutations increase its kinase activity,^5–7^ and increased Rab phosphorylation has been reported in postmortem samples from idiopathic PD patients.^8^ This has made LRRK2 one of the main actionable targets for PD therapeutics, with several clinical trials targeting LRRK2 currently under way (clinicaltrials.gov).

LRRK2 is a large (286 kDa), multidomain protein, with an N-terminal half comprising armadillo (ARM), ankyrin (ANK), and leucine-rich repeats (LRR) domains, and a C-terminal half containing both a Ras-family GTPase (Ras of complex, or ROC), and a kinase (Figure 1A). Structural studies revealed that LRRK2 is regulated by autoinhibition: the LRR, which connects to the ROC GTPase domain, covers the kinase’s active site, thus blocking access by Rab substrates (Figures 1A and 1B)^10^ and preventing the kinase from reaching its active state (Figure 1C).^10^ LRRK2 can form dimers, and structural work suggests that the highest affinity dimerization interface is the one mediated by interactions between its C-terminal of ROC (COR) domains (COR-A and COR-B)^10,11^ (Figure 1D).

Despite the importance of autoinhibition in LRRK2’s function, its mechanism and regulation are not understood. Several studies have examined the role of the ROC GTPase domain in regulating LRRK2 activity, but the results are not consistent. Earlier work showed that the addition of GTP or non-hydrolyzable GTP analogs to cell lysates correlates with an increased LRRK2 kinase activity,^12,13^ and that pathogenic mutations in the ROC-COR region can enhance GTP binding^12,14^ and affect GTP hydrolysis.^15,16^ However, while nucleotide-dependent effects were frequently observed in lysate-based assays, they were reduced or absent when nucleotides were added to immunoprecipitated or purified LRRK2, thus indicating a strong dependence on experimental and cellular contexts.^12,13^ Structurally, the direct connection between the LRR, which blocks access to the kinase active site, and the ROC domain makes its nucleotide state a prime candidate to regulate LRRK2. In support of this role, the interface between ROC and the adjacent COR-B domain is a hotspot for PD-linked mutations (Figures 1A and 1B), and some of these mutations have been shown to alter GTP binding^12,14^ or hydrolysis.^15,16^ However, no structural studies have systematically examined the effect of the ROC nucleotide state on LRRK2 activation and autoinhibition. All structures of LRRK2 reported to date where the nucleotide state of the ROC domain could be determined,^10,11,17–20^ including those capturing an active conformation,^19^ were bound to GDP.

Here, we set out to determine how the nucleotide state of the ROC GTPase regulates LRRK2 activation and autoinhibition, structurally and mechanistically. Understanding this regulation could open new therapeutic avenues to allosterically inhibit LRRK2’s kinase activity. The accumulated evidence has shown that different types of kinase inhibitors stabilize different LRRK2 conformations,^11,17,18^ and that these conformations have different cellular localizations and/or interactomes.^6,11,21–23^ Although the clinical relevance of these differences is not yet clear, they suggest that developing multiple approaches for targeting LRRK2 is desirable. In parallel, there is growing evidence that LRRK2 plays an important role in the lung and that loss of function is linked to both pulmonary fibrosis^9,24,25^ and tumorigenesis.^26^ Thus, allosteric activators of LRRK2 may be useful therapeutics as well.

The foundation of this study is an unbiased structural approach to understand whether and how the nucleotide state of LRRK2’s ROC domain correlates with LRRK2’s active and autoinhibited conformations. Throughout this work, we use “activated” to refer to any LRRK2 conformation where its N-terminal repeats are undocked, and “autoinhibited” to refer to those conformations where the N-terminal repeats are docked and thus blocking access to the kinase active site. Note that, in this context, activated refers to accessibility to the kinase active site and not to kinase activity itself.

## RESULTS

### Cryo-EM reveals that GDP loss from the GTPase domain leads to LRRK2 activation

To determine structures of LRRK2 in different nucleotide states, we used a nucleotide-exchange protocol to strip off the guanosine diphosphate (GDP) that is typically bound to purified LRRK2 (see STAR Methods). We then incubated LRRK2 with one of the following: (1) GDP, (2) the non-hydrolyzable GTP analog GMPPNP, or (3) a mixture of GDP and GMPPNP (Figure 1E). We used three different LRRK2 variants: wild type (WT) and the PD-linked mutants G2019S and R1441C (Figures 1A and 1B). G2019S, found in the kinase’s active site, is the most common PD-linked mutation in LRRK2. R1441C is found in the ROC domain at the interface between ROC and COR-B (Figures 1A and 1B). We collected data from the resulting samples and combined all the datasets into one for the initial data processing. Later, we split some of the data according to the LRRK2 variant or the nucleotide used in the samples (see STAR Methods and Figures S1–S7). All samples were incubated with the designed ankyrin repeat protein (DARPin) E11, which we previously showed binds to the WD40 domain and does not affect kinase activity *in vitro*.^27^ The reason for including DARPin E11 in our samples is that its presence increases the range of orientations LRRK2 adopts on cryo-electron microscopy (cryo-EM) grids, which leads to higher resolution structures.^27^

Data processing revealed a significant level of compositional and conformational heterogeneity in our samples, as expected, which includes multiple cryo-EM maps representing activated LRRK2 (Figures 1F and S1). We were able to build models for 16 distinct cryo-EM maps (Figures 1F and S1–S7). Nine maps corresponded to autoinhibited LRRK2 and could be classified into three states (Figure 1F): a LRRK2 monomer with the N-terminal repeats docked, the dimeric form of this species, and a mixed dimer with one LRRK2 protomer autoinhibited and the other activated. The eight maps representing activated LRRK2 could be classified into three structures (Figure 1F): the same mixed dimer (counted twice as it contains both autoinhibited and activated LRRK2); structures showing only the C-terminal half of LRRK2 containing the ROC, COR, kinase, and WD40 domains; and structures showing only the N-terminal repeats.

### Autoinhibited LRRK2 is bound to GDP

Next, we determined the nucleotide state of ROC for the highest-resolution map representing the monomeric form of autoinhibited LRRK2 (Figures 2A, S2, and S3). In agreement with previous structures,^10,17,18^ this map fitted GDP better than GMPPNP (Figures 2B and 2C). Surprisingly, we found that particles coming from samples treated with any nucleotide condition contributed to this map, with a majority coming from samples treated with GMPPNP. We then generated new maps comprising particles from samples treated either with GDP (Figure 2D) or GMPPNP (Figure 2E). The map arising from samples treated with GMPPNP also accommodated GDP better than GMPPNP (Figures 2F and 2G). We interpreted this as incomplete nucleotide stripping, with some LRRK2 molecules maintaining their originally bound GDP throughout the process.

To test this, we prepared a sample where we extended the nucleotide-stripping step from 1 to 24 h; this sample showed 100% of the particles in the activated state (Figures 2H and S8). Taken together, our data show that autoinhibited LRRK2 is in a GDP-bound state.

### Active LRRK2 is bound to GMPPNP or lacks a nucleotide

We next focused on the structures of activated LRRK2, where the N-terminal repeats are undocked and thus not seen in our cryo-EM maps because the repeats are flexible relative to the C-terminal half of LRRK2 (Figures 3A–3F, S4, and S6). The activated structures of LRRK2 showed increased conformational heterogeneity in their ROC and COR-A/B domains (Figures 3G–3J and S9; Video S1).

We obtained five volumes representing activated LRRK2 for which we were able to build models (Figures 3B–3F and S4–S6) and could determine the nucleotide state of the ROC domain for four of them; two volumes contained GMPPNP (Figures 3B and 3C) while two lacked a nucleotide (Figures 3D and 3E). These structures suggest that LRRK2 activation is coupled to the loss of GDP. One GMPPNP-bound structure of activated LRRK2 was unique because we observed density for the C-terminal portion of the LRR, which connects to the GTPase (Figure 3C), thus possibly representing an intermediate of activation or autoinhibition (Figures 3K, S9B, and S9C).

We first tested the idea that removal of GDP was sufficient to activate LRRK2 structurally by applying our nucleotide-stripping protocol to LRRK2 without adding back a nucleotide. Data processing of images collected from this sample showed that a significant fraction of the LRRK2 molecules were in their activated conformation (Figures S10A–S10C). We next tested this functionally by measuring the effect of GDP removal on LRRK2 phosphorylation of Rab8a *in vitro* (Figure 4A). Incubation of LRRK2 with EDTA increased Rab8a phosphorylation relative to untreated LRRK2 (Figures 4B and S10D).

One molecular species of LRRK2 that was conspicuously absent from our data sets was a dimer of activated LRRK2s (Figure 1F). We wondered whether activation of LRRK2 might destabilize the dimer. This was indeed the case: exposure of LRRK2 to the nucleotide-stripping protocol resulted in a shift from dimers to monomers, as measured by mass photometry (Figures 4C, 4D, and S16A).

We also solved the structure of the undocked N-terminal repeats (Figures 3L and S7), which contained a significant fraction of particles coming from monomeric activated LRRK2. This structure shows that the repeats remain structured when undocked from the C-terminal half of LRRK2. Importantly, modeling suggested that the N-terminal repeats could not redock when LRRK2 is in its activated conformation, as this would require flexibility in the repeats that we have not seen in our data (Figure S11). Thus, the combination of conformational changes at the ROC-COR interface and the rigidity of the N-terminal repeats, appear to be key elements in LRRK2’s regulation.

### The PD-linked R1441C/G/H mutations promote undocking of the N-terminal repeats

R1441 is located at the interface between the ROC GTPase and the COR-B domain (Figures 1A and 1B), a region where several PD-linked mutations cluster. To understand how the R1441C/G/H mutations increase Rab phosphorylation by LRRK2,^28^ we first compared structures of LRRK2[WT] in its autoinhibited and activated conformations (Figures 3M–3S). In GDP-bound, autoinhibited LRRK2[WT], R1441 packs against the COR-B domain, which likely contributes to the stabilization of this interface and thus to autoinhibition (Figures 3N and 3O). In contrast, the structure of GMPPNP-bound, activated LRRK2[WT] revealed a density that can accommodate R1441 in two different rotamers, one pointing toward COR-B and the other away from it (Figures 3P and 3Q); importantly, neither rotamer interacts with COR-B in the activated state, where COR-B has moved away from ROC. We hypothesize that R1441, which is far away from the nucleotide-binding pocket and thus unlikely to directly affect binding or hydrolysis, acts as a latch that helps to stabilize the ROC-COR interactions when LRRK2 returns to its autoinhibited conformation. This interaction between R1441 and COR-B is absent in the PD-linked R1441C mutant (Figures 3R and 3S), which would therefore favor the activated state of LRRK2. Cryo-EM data from LRRK2[WT] and LRRK2[R1441C] showed a moderate increase in the fraction of particles with their N-terminal repeats undocked in LRRK2[R1441C] (Figure S12). Thus, our data suggest that the increased Rab phosphorylation seen in LRRK2[R1441C/G/H] is a result of a shift in the LRRK2 population toward its activated conformation rather than a direct effect on the catalytic activity of the kinase. A test of this hypothesis is discussed below.

### Development of DARPins to control LRRK2’s activation

Given the correlation we observed between the conformations of the ROC-COR module and the N-terminal repeats and the nucleotide state of the ROC domain, we wondered whether the conformation of the N-terminal repeats could regulate nucleotide binding and hydrolysis in ROC. To test this, we needed a way to promote or prevent the undocking of the N-terminal repeats that did not require directly exchanging the nucleotide of the GTPase. We had previously identified DARPins that bound to LRRK2.^27^ The first DARPin we characterized,^27^ E11, bound to the WD40 domain in an area where it does not interact or interfere with any other part of LRRK2 and did not affect LRRK2 kinase activity *in vitro*^27^ (Figure 5K). Here, we screened for other DARPins that might alter LRRK2’s activation state and identified two additional DARPins that bind to the WD40 domain: one that activates LRRK2 (C12) and one that stabilizes its autoinhibited conformation (G10) (Figure 5). DARPin C12 binds at a location on the WD40 domain that is sterically incompatible with the docked conformation of the N-terminal repeats (Figures 5A–5D and S13). DARPin G10 binds both to the WD40 domain and to the N-terminal repeats, bridging them (Figures 5E–5H and S14). As predicted from the structures (Figures 5I and 4J), DARPin C12 increased LRRK2’s kinase activity toward Rab8a *in vitro*, whereas DARPin G10 decreased it (Figures 5K and S15).

### G2019S and R1441H increase Rab phosphorylation by LRRK2 via different mechanisms

Our structural analysis suggested that R1441C/G/H mutations activate LRRK2 by favoring the undocked conformation of the N-terminal repeats (Figures 3M–3S). G2019S, on the other hand, is in the catalytic DYG triad in the kinase’s active site. Thus, we hypothesized that these two PD-linked mutations lead to increased Rab phosphorylation in cells by acting in different steps of the LRRK2 activation/phosphorylation pathway (Figure 6A): R1441C/G/H mutations enrich the population of activated LRRK2, while G2019S activates the kinase itself. We tested this in two different ways.

First, we used the C12 DARPin, which activates LRRK2 by favoring the undocking of the N-terminal repeats. Mirroring our results *in vitro* (Figure 5K), expressing DARPin C12 in cells led to a significant increase in the phosphorylation of Rab8a by LRRK2[WT] (Figure 6B). This effect is not exclusive to Rab8a; we observed a similar increase in the phosphorylation of Rab10 (Figures S15B and S15C). If R1441C/G/H mutations increase activity by favoring undocking of the repeats, as we hypothesized, the presence of C12 should not lead to a significant additional increase in Rab phosphorylation. LRRK2[G2019S], on the other hand, acts on the kinase itself and should be further activated by C12. This was indeed the case when we measured Rab8a phosphorylation in cells co-expressing C12 and either LRRK2[G2019S] or LRRK2[R1441H] (Figures 6B and S15D).

A second prediction from our hypothesis (Figure 6A) is that the activating effects of the R1441C/G/H and G2019S mutations should be additive to some extent, as they act in series, with R1441C/G/H mutations increasing the pool of activated LRRK2 and G2019S increasing the kinase activity of that pool. Indeed, the increase in Rab8a phosphorylation in cells by the double-mutant LRRK2[R1441H/G2019S] was close to the sum of the effects seen with the single mutants (Figures 6C and S15E).

Taken together, these data support the hypothesis that even though R1441C/G/H and G2019S both lead to an increase in Rab phosphorylation in cells, they do so by acting on different steps in the LRRK2 pathway.

### Nucleotide exchange and hydrolysis by ROC are regulated both by the N-terminal repeats and the Rab substrate

We next tested the effect of DARPins C12 and G10 on the GTPase activity of LRRK2. Our hypothesis (Figure 7A) was that G10 would stabilize the autoinhibited conformation of LRRK2 and thus prevent cycles of nucleotide exchange and hydrolysis, while C12 would stimulate them by promoting the undocking of the N-terminal repeats. Our initial data agreed with our prediction that G10 would inhibit the GTPase activity of LRRK2; however, C12 had no effect on it (Figure 7B). We wondered whether undocking of the N-terminal repeats was not sufficient to stimulate GTP hydrolysis, and if adding a Rab substrate might do so. Indeed, the combination of C12 and Rab8a (the Q67L variant that is locked in its GTP-bound state) led to a significant activation of the GTPase (Figure 7C). Importantly, Rab8a[Q67L] on its own had no effect on the baseline GTPase activity of LRRK2 (Figure 7C), which suggests that the undocking of the N-terminal repeats by C12 is required for Rab8a to exert its effect on hydrolysis.

The effect of Rab8a on the rate of GTP hydrolysis could be the result of an increase in hydrolysis itself, an increase in the rate of GDP release, or a combination of both. To address this, we measured the rate of GDP release from LRRK2 pre-loaded with a fluorescently labeled nucleotide in a single-turnover assay (see STAR Methods). Consistent with our model where the undocking of the N-terminal repeats lowers the affinity for GDP, the rate of GDP release from LRRK2[WT] was increased by the addition of DARPin C12 (Figure 7D). The addition of Rab8a on its own had no effect on GDP release, but the combination of C12 and Rab8a led to a small but significant increase relative to C12 alone, thus indicating that Rab8a is promoting GDP release (Figure 7D).

Last, we wanted to confirm that the effects we were seeing with C12 were due to its promoting the undocked conformation of LRRK2. For this, we turned to LRRK2[R1441C], which our data showed favors the undocked conformation of LRRK2 (Figure 6). Consistent with those results, GDP release from LRRK2[R1441C] was faster than from LRRK2[WT] and was in turn insensitive to the addition of C12 (Figure 7D). Finally, addition of Rab8a to LRRK2[R1441C] significantly increased GDP release, and was again insensitive to C12, thus supporting the idea that Rab8a is acting downstream of the undocking of the N-terminal repeats. Taken together, our data showed that Rab8a stimulates both GDP release and GTP hydrolysis in activated LRRK2.

## DISCUSSION

### The nucleotide state of the ROC GTPase controls LRRK2 activation and autoinhibition

The work presented here, together with contributions from others in the field, has led to a model for how the activation and autoinhibition of LRRK2 are regulated (Figure 7E; Video S2). The autoinhibited conformation of LRRK2 was always accompanied by a ROC domain bound to GDP in our structures (Figure 2), which is in agreement with all the structures of this species published to date where the nucleotide state could be determined.^10,17,18^ This suggests that autoinhibited LRRK2 has high affinity for GDP (Figure 7Ea), which is consistent with our observation that complete stripping of GDP from autoinhibited LRRK2 requires long treatment with EDTA (Figures 2H and S8).

Our model proposes that undocking of the N-terminal repeats and the resulting flexibility in the ROC-COR module lead to a decrease in the ROC domain’s affinity for GDP (Figures 7Eb–7Ee). This is supported by the increase in the rate of GDP release from LRRK2[R1441C], a mutant that favors the undocked conformation of the N-terminal repeats (Figures 3N–3S and 6). It is likely that any protein that interacts with LRRK2 and stabilizes its N-terminal domains in the undocked conformation will act as an activator by promoting nucleotide exchange. While a few activators have been identified—including Rab12,^29,30^ Rab32,^31,32^ and GABARAP^33,34^—we do not yet understand whether they exert their effects by recruiting LRRK2 to membranes or additionally by stabilizing the undocked conformation once LRRK2 is on the membrane.

Our data also revealed that the Rab substrate plays a role in stimulating the GTPase cycle in the ROC domain, thus accelerating both GDP release and GTP hydrolysis (Figures 7C, 7D, and 7Ee–7Eg). This mechanism allows the N-terminal repeats to act as a substrate sensor; promoting GTP hydrolysis to return LRRK2 to its GDP-bound state puts LRRK2 in a state compatible with a return to autoinhibition, a process that is prevented if Rab substrate is available to sterically block redocking of the N-terminal repeats (Figure 7Ec). If redocking is prevented, ROC-COR maintains its low affinity for GDP (Figure 7Ee), thus leading to GDP release, GTP binding, and a return of LRRK2 to its stably active conformation (Figures 7Eg and 7Eh). Given the multiple Rab-binding sites reported for LRRK2,^35,36^ we cannot say at this point where Rabs exert these activating effects we observed.

Several studies have addressed the role of the GTPase in regulating LRRK2 activity. While some are consistent with the work we presented here, others are not. In general, work done in cells or with lysates agrees with our observations: the addition of GTP or non-hydrolyzable GTP analogs to cell lysates correlates with increased LRRK2 kinase activity.^12,13^ Similarly, LRRK2 localization and association with microtubules in cells correlate more closely with the ability to bind GTP than with kinase activity,^14^ and we now understand that binding to microtubules requires LRRK2 to be in its active conformation.^11,37,38^ Also in agreement with our data are observations that pathogenic mutations in the ROC-COR region can enhance GTP binding^12,14^ and affect GTP hydrolysis,^15,16^ and that disruption of guanine nucleotide binding by mutations in the P-loop of the GTPase domain (e.g., K1347A or T1348N) generally impaired kinase activity and showed cellular phenotypes across multiple systems.^13,39,40^ However, nucleotide-dependent effects were reduced or absent when nucleotides were added to immunoprecipitated or purified LRRK2, thus indicating a strong dependence on experimental and cellular context.^12,13^ In addition, measurements of GTPase activity have yielded inconsistent results: while some studies reported low intrinsic hydrolysis and predominantly GTP-bound LRRK2 in cells,^39^ others showed that mutations that either increase or decrease GTP hydrolysis reduce kinase activity,^40^ which suggests that nucleotide binding is more important than hydrolysis in regulating kinase activity. Finally, there is evidence pointing to a role for autophosphorylation of the ROC domain in regulating GTP hydrolysis.^16^ It is difficult to determine where discrepancies arise as the studies are not directly comparable in terms of experimental approaches. Our work has shown that the activation state of LRRK2 is sensitive to how the protein is treated in guanine nucleotide-exchange protocols. Given this, it is likely that some of the disagreements in the literature reflect differences in the distribution of active and autoinhibited populations of LRRK2 in cells and lysates vs. purified protein, or because of differences in the nucleotide-exchange protocols used.

Finally, our data also showed that the common PD-linked mutations R1441C/G/H and G2019S act on different steps of the activation mechanism and provided an explanation for how PD-associated mutations found on either side of this ROC-COR interface (e.g., N1437H and Y1699C) lead to increased Rab phosphorylation.

### The active form of LRRK2 is likely a monomer

Several lines of evidence lead us to propose that the active form of LRRK2 is a monomer. Previous work has shown that disruption of dimerization is activating.^7,38^ Stripping LRRK2 of its bound GDP, a process that is activating and leads both to undocking of the N-terminal repeats (Figure S10) and an increase in kinase activity *in vitro* (Figures 4A, 4B, and S11), correlates with a shift in the LRRK2 population from dimer to monomer (Figures 4C, 4D, and S16A). In agreement with these data, modeling a dimer in which both LRRK2s are activated showed that the conformational changes in the COR-A and COR-B domains involved in activation lead to significant changes and clashes at the dimer interface that make this species unlikely (Figures S16B–S16H).

In contrast to our observations and proposal, it has been suggested that LRRK2 is activated in the context of a tetramer.^19^ This model was based on a cryo-EM structure where LRRK2 formed a dimer of dimers, with each dimer containing one protomer of autoinhibited LRRK2 and one protomer of activated LRRK2, with its repeats undocked. LRRK2 is known to dimerize, and structural work suggests that the highest affinity dimerization interface is the one mediated by interactions between its COR-A and COR-B domains^10,11^ (Figure 1D); this was the interface mediating the dimers that formed the tetramer. Given this, the model predicts that disrupting dimerization at the COR interface, which would disrupt the tetramer, should decrease LRRK2 activity. However, the opposite is true: disrupting the dimer interface increases Rab phosphorylation, both *in vitro*^7^ and in cells.^7,38^ While the physiological relevance of the reported tetramer remains to be determined, it is possible that its appearance was a consequence of the high concentrations of LRRK2 used for cryo-EM (21–28 μM).^19^

### The ROC-COR module as a regulatory nexus

Our work points to the ROC-COR module as a regulatory nexus in LRRK2, with its conformation being coupled to the nucleotide state of the ROC GTPase. A recent cryo-EM structure of a bacterial Roco protein (which contains the LRR-ROC-COR module but lacks a kinase) showed the undocking of its LRR domain when its GTPase was in a GTP-like state.^41^ We propose that stabilizing the ROC-COR module in a specific conformation may be a strategy that other regulatory factors use. Identifying such factors may lead to novel targets for PD therapeutics.

The best-established regulators of LRRK2 are 14–3-3 proteins.^42–45^ These proteins, which function as dimers, bind to phosphorylated residues (pS910 and pS935) in a flexible loop in the LRR domain, and are understood to keep LRRK2 in its autoinhibited form in the cytosol.^22^ The pS910 and pS935 marks are used as biomarkers in the diagnosis of LRRK2-linked PD, as the majority of pathogenic mutations that increase LRRK2 activity suppress biomarker site phosphorylation.^23^

A structure of LRRK2 bound to 14–3-3 was recently reported.^20^ In this structure, a 14–3-3 dimer was bound simultaneously to the COR-A and COR-B domains of LRRK2, and densities for two phosphoserines were observed interacting with 14–3-3. The authors proposed that 14–3-3 maintains LRRK2 in an autoinhibited state by restricting the movement of the N-terminal repeats, as these are tethered to 14–3-3 via the loop containing pS910 and pS935. The authors also noted that 14–3-3 could only fully engage with autoinhibited LRRK2.^20^

Based on the data presented here, we propose that the role of the pS910/pS935 residues in the loop is not to restrict the conformation of the N-terminal repeats but rather to recruit 14–3-3 to LRRK2, as the loop is long enough to accommodate undocking even when bound to 14–3-3. In this model, 14–3-3’s main function would be to clamp together the COR-A and COR-B domains and prevent the conformational changes that lead to GDP release and LRRK2 activation.

Interestingly, the binding of 14–3-3 to LRRK2 prevents dimerization by blocking the COR-COR interface.^20^ Since LRRK2 dimerization and 14–3-3 binding both engage COR-A and COR-B in their autoinhibited conformation, they could be alternative mechanisms to prevent the conformational changes required for LRRK2 activation. Understanding the interplay among these different regulatory mechanisms could also lead to novel therapeutic targets.

### Nucleotide control of the docking and undocking of the N-terminal repeats

Our structural data suggest a mechanistic model for how the nucleotide state of ROC might control the docking and undocking of the N-terminal repeats (Figure S17). The LRRs are connected to the ROC-COR module in the autoinhibited state of LRRK2 via a short two-stranded β-sheet (Figures S17A and S19B). At one end, both β-strands connect to the ROC domain. At the other end, they connect to different domains: one from the LRR to ROC and the other from ROC to COR-A. This arrangement means that changes in the relative orientations of ROC and COR-A, as happens during activation of LRRK2, would destabilize this β-sheet. In fact, the β-sheet is absent from all our structures of activated LRRK2. We only saw it in the structure of GMPPNP-bound activated LRRK2, where a partial density for the LRR is present (Figures S17C–S19F). The potential importance of this β-sheet is highlighted by the high conservation of the sequences that encompass it (Figures S17G and S19H). A closer look at changes in the ROC domain that correlate with its nucleotide state suggests that a conformational switch may be controlling the presence or absence of the β-sheet, with GDP stabilizing it and GMPPNP leading to its disruption (Figures S17I–S19L).

### Roadmap for therapeutics

The conformation of the ROC-COR module changes when the N-terminal repeats undock and no longer constrain it (Figures 7Ec–7Ee). Additional changes in the interfaces between the ROC and COR domains occur in response to the nucleotide bound to ROC (Figures 3G–3J; Video S1). These changes provide a roadmap for identifying small molecules that stabilize either the GDP-bound autoinhibited LRRK2 or the GTP-bound or Apo activated LRRK2. The importance of the interface between ROC and COR-B for PD has been appreciated for a long time, as it is a hotspot for disease-linked mutations (Figures 1A and 1B). Molecules that stabilize the autoinhibited LRRK2 would represent a novel category of allosteric LRRK2 kinase inhibitors for the treatment of PD, which are distinct from the type-1 kinase inhibitors that are and were previously used in clinical trials (clinicaltrials.gov). Molecules that activate LRRK2 could also be interesting therapeutically, as loss of LRRK2 activity has been linked to both lung fibrosis^9,24^ and lung tumorigenesis^26^ in rodents and lung fibrosis in humans.^25^

### Limitations of the study

Our model for how the nucleotide state of the ROC domain controls the activation and autoinhibition of LRRK2 is largely based on the correlation between nucleotide state and conformation in our cryo-EM data. Direct evidence for the interplay between the conformation of LRRK2 and nucleotide binding and/or hydrolysis at the ROC domain would require single-molecule fluorescence resonance energy transfer (FRET) experiments that simultaneously measure conformational changes and nucleotide binding and release. Additionally, our inferences about the relative affinities for the different nucleotides—a high affinity for GDP in autoinhibited LRRK2 and a lower affinity for GDP and a preference for GTP in the activated state—are based on the nucleotide states seen in cryo-EM data and how these nucleotide states vary in response to the nucleotide stripping and exchange protocol. Because our data suggest that nucleotide affinity is conformation dependent, obtaining dissociation constants would be challenging experimentally. Finally, although we proposed a mechanism for how the nucleotide state of ROC might control docking and undocking of the N-terminal repeats, this model remains hypothetical. Additional structures and structure-guided functional assays will be required to test it.

## RESOURCE AVAILABILITY

### Lead contact

Requests for further information and resources should be directed to and will be fulfilled by the lead contact, Andres E. Leschziner (ale4009@med.cornell.edu).

### Materials availability

All unique/stable reagents generated in this study are available from the lead contact upon request.

### Data and code availability

All cryo-EM maps and models have been deposited in the EM Data Bank and Protein Data Bank, respectively. All accession numbers are listed in Table S1.

## STAR★METHODS

### EXPERIMENTAL MODEL AND STUDY PARTICIPANT DETAILS

#### Mammalian Cells

All cellular assays were conducted using 293Ts (ATCC, RRID: CVCL_0063). Cells were cultured in Dulbecco’s Modified Eagle’s Medium (Corning) with 10% FBS and 1X Penicillin-Streptomycin (Corning) at 37 °C in 5% CO2.

#### Insect Cells

For protein expression, SF9 insect cells (RRID:CVCL_0549) were cultured with a density of 2×10^6^ cells/ml in serum-free Insect-XPRESS Medium (Lonza) at 27°C.

### METHOD DETAILS

#### Expression and purification of LRRK2

For LRRK2 expression, DNA coding for full-length LRRK2-WT (residues 1–2527) or LRRK2^RCKW^ (residues 1327–2527) (from the Mammalian Gene Collection) was polymerase chain reaction (PCR)-amplified, and the amplicon was inserted into the expression vector pKL: (RRDID:Addgene_110741). The plasmid was used as template for G2019S and R1441C site-directed mutagenesis (Q5 Site-Directed Mutagenesis Kit, NEB). The resulting plasmids were used to generate recombinant baculovirus, according to BAC-to-BAC expression system protocols (Invitrogen). The virus was used to infect SF9 insect cells (RRID:CVCL_0549) with a density of 2×10^6^ cells/ml. Infected cells were cultured in serum-free Insect-Xpress Medium (Lonza) at 27°C for 3 days post infection. For LRRK2 purification, pelleted Sf9 cells were washed with phosphate-buffered saline (PBS), resuspended in lysis buffer [50mM Hepes (pH 7.4), 500mM NaCl, 20mM imidazole, 0.5mM TCEP, 5% glycerol, 5mM MgCl_2_, and 20μM GDP] and lysed by homogenization. The supernatant was cleared by centrifugation and loaded onto a Ni-NTA (Qiagen) column. After rinsing with lysis buffer, the His_6_-Z–tagged protein was eluted in lysis buffer containing 300mM imidazole. The eluate was then diluted to 250mM NaCl with dilution buffer [50mM Hepes (pH 7.4), 0.5mM TCEP, 5% glycerol, 5mM MgCl_2_, and 20μM GDP] and loaded onto an SP Sepharose column (Cytiva). His_6_-Z-TEV-LRRK2^RCKW^/His_6_-Z-TEV-LRRK2 were eluted with a gradient of 250mM to 2.5 M NaCl in dilution buffer and then treated with recombinantly expressed Tobacco Etch Virus (TEV) protease overnight to cleave the His_6_-Z. Contaminating proteins, the cleaved tag, uncleaved protein, and TEV protease were removed by another combined SP sepharose Ni-NTA (Cytiva) step. Lastly, both LRRK2 and LRRK2^RCKW^ were concentrated and subjected to gel filtration in 20mM Hepes (pH 7.4), 700mM NaCl, 0.5mM TCEP, 5% glycerol, 2.5mM MgCl_2_, and 20μM GDP, using an ÄKTAxpress system with an S200 16/200 gel filtration column (GE Healthcare). Final yields, as calculated from ultraviolet absorbance, were in the range of 0.9 to 2.2 mg for LRRK2 and LRRK2^RCKW^ respectively per liter of insect cell medium.

#### Expression and purification of DARPins E11, C12 and G10

DARPin E11, C12 and G10 were cloned into a pQE30 vector (Qiagen) containing an N-terminal 8× His tag and a C-terminal 3x-FLAG tag, as previously described.^27^ Plasmids were transformed into *E. coli* Rosetta cells. Cells were grown at 37 °C with shaking until the *A*_600_ reached 1.0. Then, the temperature was reduced to 18 °C and protein expression was induced by adding 0.5mM IPTG. After 18 (H) the cells were harvested by centrifugation and resuspended in lysis buffer (50mM Hepes pH 7.4, 500mM NaCl, 20mM imidazole, 0.5mM TCEP, and 5% glycerol) prior to lysis by sonication. The lysate was cleared by centrifugation and loaded onto preequilibrated Ni-NTA Sepharose beads (GE Healthcare). The beads were washed with 50 column volumes of lysis buffer and protein was eluted in lysis buffer supplemented with 300mM imidazole. The eluate was concentrated to 5 ml and subjected to SEC using an S200 16/200 column (GE HealthCare) in SEC buffer (20mM Hepes pH 7.4, 150mM NaCl, 0.5mM TCEP, and 5% glycerol). Final concentrations for DARPin E11, C12 and G10 were: 6.3mg/mL, 3.6mg/mL and 3mg/mL respectively.

#### Kinase activity assays with DARPins

The impact of DARPin binding on kinase activity was assessed by measuring the phosphorylation of Rab8a. The reaction was set up using 100nM LRRK2 and incubated for 40 min at room temperature with 1.5μM His-Rab8a T22N and with or without 1mM ATP, 10μM DARPin (E11, C12, or G10), or 2.5μM Mli-2 in reaction buffer (50mM HEPES pH 7.4, 150mM NaCl, 0.5mM TCEP, 5mM MgCl_2_, 5% glycerol, 20μM GTP, 2μM GDP). Reactions were quenched using SDS-PAGE sample buffer, run on an SDS-PAGE gel, and then transferred via Western blot for immunoblot analysis to visualize LRRK2 (1:1000, MJFF2 (c41–2); Abcam ab133474, Lot # GR3445288–3; RRID:AB_2713963), His-Rab8a (1:2000, 6*His Monoclonal antibody; Proteintech 66005, Lot # 10027680; RRID: AB_3086567), and phospho-Rab8a (pT72) (1:500, Abcam 230260, Lot #GR3216587–1;RRID:AB_2814988). All membranes were imaged using the Biorad Chemidoc MP Imaging system. Band intensities were quantified using ImageJ (version 2.14.0). One-way ANOVA with Tukey’s multiple comparison correction was performed using GraphPad Prism (version 10.5.0, RRID: SCR_002798) on the ratio of background corrected phospho-Rab in each condition to the average of phospho-Rab in the LRRK2 alone condition (n=3).

#### Kinase activity assay with nucleotide-stripped LRRK2

LRRK2[WT] was incubated with (‘EDTA treated) or without EDTA (‘mock treated’) for 24 h followed by addition of 20mM MgCl_2_ and further incubation for 24 h. Untreated LRRK2 was used immediately upon thawing from frozen aliquots. The effect of nucleotide stripping on kinase activity was assayed by measuring the phosphorylation of Rab8a as described above. The reaction was set up using 100nM LRRK2 and incubated for 40 min at room temperature with 1.5μM His-Rab8a T22N and with or without 1mM ATP, in reaction buffer (50mM HEPES pH 7.4, 150mM NaCl, 0.5mM TCEP, 5mM MgCl2, 5% glycerol). All LRRK2 samples were incubated with Rab8a in the presence of ATP, with one negative control lacking ATP. Background-corrected pRab8a was normalized to the average pRab8a in the untreated LRRK2 + ATP condition from [*n*=3] independent Western blots. Images were processed in ImageJ.

#### Guanine nucleotide exchange of LRRK2 samples for cryo-EM

We prepared a working stock of 0.1M EDTA (pH 7.5) by diluting 0.2M EDTA pH 7.5 in half with Exchange Buffer (20mM Hepes pH 7.4; 200mM NaCl and 20μM TCEP). Ten μL aliquots of full-length LRRK2 at concentrations of 11–20μM were mixed with 0.5μL of 0.1M EDTA (pH 7.5) (for a final EDTA concentration of 5mM) and incubated for 30 min at 4 °C. After the incubation, a Zeba micro desalting column (Thermo Fisher Scientific) was equilibrated with Exchange buffer according to the manufacturer’s instructions. A total of 10.5 μL of sample was applied to the spin column, followed by 3 μL of Exchange Buffer, and centrifuged for 2 min at 1000 × *g* at 4 °C. After centrifugation, 1.3 μL of 10mM nucleotide (GMPPNP, Abcam AB146660, or GDP, Sigma-Aldrich G7127) was added followed by 1.3 μL of 20mM MgCl_2_ to the 13.5 μL sample, for a total volume of 16.1 μL and a final concentration of 0.8mM nucleotide and 1.6mM MgCl_2_. For mixed-nucleotide samples, 1.3 μL of 750μM GMPPNP and 250μM GDP were added, followed by 1.3 μL of 20mM MgCl_2_ to the 13.5 μL sample, for a total volume of 16.1 μL and a final concentration of 0.8mM total nucleotide (200μM GDP and 600μM GMPPNP) and 1.6mM MgCl_2_. Samples were incubated for 30–60 min at 4 °C.

Some of our initial cryo-EM analysis revealed that the nucleotide stripping protocol described above was incomplete, and that some LRRK2 remained bound to GDP even in samples that had been stripped and incubated with GMPPNP (Figure 2). To address this issue, we tested a longer EDTA treatment. Ten μL of purified full-length LRRK2 (15μM) was incubated with 5mM EDTA (pH 7.5) for 24 hr at 4 °C. After incubation, a Zeba micro desalting column was equilibrated with Exchange buffer as described above. A total of 10.5 μL of sample was applied to the spin column, followed by 3 μL of Exchange buffer, and centrifuged for 2 min at 1000 × *g* at 4 °C. After centrifugation, 1.3 μL of 10mM GMP-PNP was added, followed by 1.3 μL of 20mM MgCl_2_, and the sample was incubated for 24 hr at 4 °C, for a final concentration of 0.8mM nucleotide and 1.6mM MgCI_2_.

#### Guanine nucleotide exchange of LRRK2 samples for kinase activity and fluorescence measurements

Full-length human LRRK2 at 20μM was thawed on ice and diluted to 500nM in a nucleotide stripping buffer (50mM HEPES, 150mM NaCl, 1mM TCEP, 5mM EDTA, and 1mM GMPPNP or GDP). Because of the incomplete nucleotide exchange we detected in our cryo-EM samples that had been stripped of their nucleotide for 30–60 min at 4 °C, each sample was incubated at room temperature for 3 h. We verified that LRRK2 was stable at room temperature for this long by measuring fluorescence emission spectra of LRRK2 incubated at room temperature for up to 3 h (Figure S18). After the stripping, 10mM MgCl_2_ was added to neutralize EDTA, then samples were incubated for another 30 min at room temperature and nucleotide (GMPPNP or GDP) was added to a final 100μM. Each sample (LRRK2 with GMPPNP or LRRK2 with GDP) was transferred into mini dialysis devices (Thermo Scientific, 69572) and dialyzed against 300 mL of nucleotide exchange buffer (50mM HEPES, 150mM NaCl, 5mM MgCl_2_, 1mM TCEP, and 0.1μM GMPPNP or GDP) overnight in the cold room with gentle stirring. The next day samples were spun down on a tabletop centrifuge at maximum speed and directly used for the measurements.

#### Guanine nucleotide exchange for mass photometry

12.7μM of LRRK2[WT] in 5 μL of buffer (20mM Hepes pH 7.4, 150mM NaCl, 5% glycerol, 0.5mM TCEP, 20uM GDP and 2.5mM MgCl2 ) was mixed with one of the following: (1) 0.28 μL of 100mM EDTA pH 7.5 and 0.28 uL of 100μM GDP (‘EDTA treated’), or (2) 0.28 μL of milliQ H2O and 0.28 μL of 100μM GDP (‘mock treated’). Samples were incubated on ice for 2 h, at which point 0.6 μL of 20mM MgCl_2_ were added and samples were incubated on ice for another 24 h. Samples were then diluted using with 20mM Hepes pH 7.4, 150mM NaCl, 5% glycerol, 0.5mM TCEP, and 1mM MgCl_2_, to a final concentration of LRRK2[WT] of 100nM and incubated on ice for 30 min before mass photometry measurements.

Total particle counts corresponding to the molecular weights of either monomer or dimer were determined from mass photometry traces. The percentage of dimers contributing to total monomer/dimer LRRK2 landing events were analyzed with one-way analysis of variance (ANOVA) and corrected for multiple comparisons using Tukey’s test.

#### Sample preparation for Cryo-EM: nucleotide-exchanged LRRK2

LRRK2, following nucleotide stripping and exchange, was incubated with MLi-2 (10mM; Tocris, RRID: Addgene_226784), and E11-DARPin, purified as previously described.^27^ The incubation was performed at a molar ratio of 1:1.5:1.25 (LRRK2:MLi-2:E11) for 10 min at room temperature, followed by 15 min at 4 °C. The resulting complex was diluted to a final concentration of 7μM LRRK2 in Exchange Buffer (20mM HEPES, pH 7.4, 200mM NaCl, 20μM TCEP) and supplemented with 21μM DDM (GOLDBIO: DDM5_69227). A volume of 2.8–3.5 μL was applied to glow-discharged UltrAuFoil Holey gold 300 mesh R1.2/1.3 grids (Quantifoil, Q350AR13A) and incubated in a FEI Vitrobot IV chamber at 4 °C and 95 % humidity for 5 s. Excess liquid was blotted for 2 s using filter paper 595 at blot force 4, and the grids were vitrified by plunging into liquid ethane cooled to liquid-nitrogen temperature.

One data set for LRRK2^R1441C^ was acquired on a Talos Arctica (FEI) operated at 200 keV and equipped with a Falcon 4i detector (Thermo Fisher Scientific). Images were recorded at a nominal magnification of 150,000× in EF-TEM mode (0.95 Å/pixel) with a cumulative electron exposure of approximately 55 e^−^/Å^2^. The remaining data sets for LRRK2^WT^, LRRK2^G2019S^, and LRRK2^R1441C^ were acquired on a Titan Krios G3 (Thermo Fisher Scientific) operated at 300 keV, equipped with a Falcon 4 direct electron detector and a Gatan BioContinuum energy filter. Images were collected at a nominal magnification of 130,000× in EF-TEM mode (0.935 Å/pixel) using a 20 eV energy filter slit width, with a cumulative electron exposure of approximately 55 e^−^/Å^2^.

#### Sample preparation for Cryo-EM: DARPin-bound LRRK2 and LRRK2^RCKW^

For LRRK2 bound to G10-DARPin, a 1:5 molar ratio (LRRK2:G10) was incubated for 10 min at room temperature, followed by 15 min at 4 °C. The complex was diluted to a final concentration of 5μM LRRK2 in buffer containing 20mM HEPES (pH 7.4), 200mM NaCl, 20μM TCEP, 20μM GDP, 2.5mM MgCl_2_, and 5 % glycerol, and supplemented with 21μM DDM (GOLDBIO: DDM5_69227). A 2.8 μL volume was applied to glow-discharged UltraAUFoil Holey gold 300 mesh R1.2/1.3 grids (Quantifoil, Q350AR13A) and incubated in a FEI Vitrobot IV chamber at 4 °C and 95 % humidity for 5 s. Excess liquid was blotted for 2 s using filter paper 595 at blot force 4, and the grids were vitrified by plunging into liquid ethane cooled to liquid-nitrogen temperature. Data was collected under the same conditions described above on a Titan Krios G3.

As previously described^27^ LRRK2^RCKW^[R1441H]:C12-DARPin was prepared by desalting LRRK2 into a buffer containing 20mM HEPES (pH 7.4), 150mM NaCl, 20μM TCEP, 20μM GDP, 2.5mM MgCl_2_, and 5 % glycerol using the Zeba micro desalting column (Thermo Fisher). LRRK2^RCKW^ was then incubated with C12-DARPin in a ratio of 1:1.25 (LRRK2:C12) for 10 min at room temperature, followed by 15 min at 4 °C. The sample was diluted to a final LRRK2^RCKW^ concentration of 6μM. A 4 μL volume was applied to glow-discharged UltrAUFoil Holey gold 300 mesh R1.2/1.3 grids (Quantifoil, Q350AR13A) and incubated in a FEI Vitrobot IV chamber at 4 °C and 95 % humidity for 20 s. Excess liquid was blotted for 4 s using filter paper 595 at blot force 4, and the grids were vitrified by plunging into liquid ethane cooled to liquid-nitrogen temperature. Images were collected at a nominal magnification of 36000x in EF-TEM mode (1.16 Å/pixel), with a cumulative electron exposure of approximately 51 e^−^/Å^2^. Data was collected on a FEI Talos Arctica operated at 200 kV and equipped with a K2 summit direct electron detector (Gatan).

#### Cryo-EM data data processing and model building

Movies for all datasets were pre-processed using Patch Motion Correction and Patch CTF estimation in CryoSPARC^46^ (RRID:SCR_016501) to perform motion correction and estimate the contrast transfer function (CTF). Micrographs with a CTF fit worse than 5.5 Å were excluded from further processing. Particles were picked for all datasets using a Topaz^47^ model (version 0.2.4, https://cb.csail.mit.edu/topaz/) previously trained on the dataset. For combined datasets, several additional rounds of Topaz training and two-dimensional (2D) classification were performed. Particle stacks were extracted at the original pixel size. Ab initio and heterogeneous refinement jobs were then used to discard poor-quality particles, followed by non-uniform refinement and local refinement (see Figures S1–S6, S9, S13, and S14). To build the initial LRRK2 model, the highest-resolution local maps obtained for the LRRK2:G10 complex were used (EMD-70981). The available structure of full-length LRRK2^10^ (PDB: 7LHW) was used as the starting point, yielding the final model PDB: 9OXH. This model was subsequently split into domains, docked into the corresponding cryo-EM maps using UCSF ChimeraX^48^ (version 1.9, RRID:SCR_015872), and merged into one chain. For models containing density for MLi-2, we built the model for the part of the map corresponding to the kinase and WD40 domains starting from our previous structure of MLi-2-bound LRRK2^RCKW^ (PDB: 8TXZ).^17^ For DARPin C12 and G10, initial models were generated using AlphaFold 3.^49^ Models were fit into the density maps using Coot^50^ (version 0.9.8, www2.mrc-lmb.cam.ac.uk/personal/pemsley/coot/, RRID:SCR_014222) and refined in Phenix to generate the final models. Maps for LRRK2^WT^[GMPPNP] and LRRK2^R1441C^ were improved with Resolve in Phenix.^51^ Data collection and refinement statistics are summarized in Table S1.

Movies for LRRK2^RCKW^:C12 DARPin complex, were collected and pre-processed using Patch Motion Correction and Patch CTF estimation in CryoSPARC^46^ (RRID:SCR_016501). Micrographs with CTF fit worse than 4 Å were excluded from further processing. Particles obtained with blob picker in CryoSPARC were used to train a Topaz^47^ model. After several rounds of 2D Classification, particles were extracted with a 288-pixel box and used for subsequent processing. Two final local refinements were performed using focused masks. The first refinement targeted the C-lobe of the kinase, the WD40 domain, and the C12 DARPin, while the second focused on the ROC domain, COR-A, COR-B, and the N-lobe of the kinase. These refinements yielded maps at 3.48 Å and 3.6 Å resolution, respectively. Local refinement procedures were conducted to evaluate map quality and directional resolution anisotropy. The available structure of LRRK2^RCKW^ (PDB:6VP7) was fitted into the 3D maps using UCSF ChimeraX^48^ and used as a starting point for model building, which was performed in Coot.^50^ The built structures were refined using real-space refinement as implemented in Phenix^51^ Data collection and refinement statistics are summarized in Table S1.

#### Quantitation of the distribution of autoinhibited and activated LRRK2 in samples treated with EDTA for 1h vs. 24h

This analysis included particle counts from samples subjected to the nucleotide stripping protocol for 1h and then incubated with GDP and/or GMPPNP yielding active volumes (see Figures S4C–4D for monomers and Figure S2F for mixed dimers) or autoinhibited volumes (see Figures S2C–2D and S2F for mixed dimers), excluding data for the mutants (LRRK2[G2019S] and LRRK2[R1441C]). The counts were then further separated according to the nucleotide added after stripping. We also used particle counts from LRRK2 subjected to 1h nucleotide stripping without subsequent addition of nucleotide (see Figure S8) and data from LRRK2 subjected to 24h nucleotide stripping followed by addition of GMPPNP (see Figures S5D–5F). The final relative abundance of particles corresponding to the active and autoinhibited states of LRRK2[WT] in the different samples is presented in Figures 2H and S8D.

#### Cryo-EM data collection for untreated (no nucleotide stripping) LRRK2[WT] and LRRK2[R1441C]

Cryo-EM data were collected on a Talos Arctica transmission electron microscope operated at 200 kV equipped with a Gatan K3 direct electron detector. Images were acquired at a nominal magnification of 36,000×, corresponding to a calibrated super-resolution pixel size of 0.5505 Å/pixel (1.101 Å/pixel in counting mode). Movies were collected using Leginon v3.7 at a dose rate of 21.22 e^−^/Å^2^/s over 2.40 s (total dose 50.92 e^−^/Å^2^), fractionated into 40 frames. A total of 2,371 movies were collected over a defocus range of 1.7–2.6 μm. Ice thickness was estimated as described previously.

On-the-fly processing was performed using MotionCor2 for motion correction and CTFFIND4 for CTF estimation within the Appion pipeline. Motion-corrected, dose-weighted micrographs (1.101 Å/pixel) were imported into cryoSPARC. Initial particle picking was performed using Warp, and picks from each dataset (WT and R1441C) were used to generate templates for cryoSPARC-based template picking.

#### Cryo-EM data processing for untreated (no nucleotide stripping) LRRK2[WT] and LRRK2[R1441C]

Motion-corrected micrographs from WT and R1441C datasets were combined prior to downstream processing to ensure consistent classification and minimize bias. After curation based on CTF quality and ice thickness (2,257 micrographs), initial particle picking was performed in cryoSPARC using template-based methods, yielding 1,420,224 particles. Representative particles were selected and used for Topaz-based particle picking, resulting in a refined dataset of 424,769 particles. This was subjected to two rounds of 2D classification to remove junk particles, yielding a final cleaned subset of 92,497 particles for downstream analysis.

These particles were used for ab initio reconstruction and heterogeneous refinement to separate junk, flexible, and autoinhibited conformations. Particles corresponding to autoinhibited states were selected, re-centered, and re-extracted at 3.03 Å/pixel (32,759 particles). These particles were further classified by ab initio reconstruction and heterogeneous refinement to resolve autoinhibited monomeric and dimeric states. The autoinhibited dimer subset was subjected to 3D classification in cryoSPARC into two classes, yielding a mixed monomer/dimer class (11,468 particles) and a dimer class (9,033 particles).

Final particle distributions were determined by tracing particles back to their dataset of origin following joint classification. Particles were grouped into four structural categories: Activated LRRK2 (“RCKW”), Autoinhibited Monomer, Autoinhibited Dimer, and Mixed Dimer. The mixed monomer/dimer class was conservatively treated as contributing equally to active and inactive states for downstream quantification.

#### GTP turnover assay

GTP hydrolysis was monitored using a real-time inorganic phosphate detection assay (EnzCheck Phosphate Assay Kit; Thermo Fisher Scientific/Invitrogen, E6645). This assay relies on the enzymatic conversion of the chromogenic substrate 2-amino-6-mercapto-7-methylpurine ribonucleoside (MESG) by purine nucleoside phosphorylase (PNP) in the presence of inorganic phosphate. Phosphate released during GTP hydrolysis drives the MESG/PNP reaction, resulting in a spectral shift in absorbance from 330 nm to 360 nm, which was continuously recorded using a spectrophotometer (Agilent G6860AA). Reactions were carried out in 60 μL volumes in quartz cuvettes and contained 1x reaction buffer (50mM Tris-HCl, 150mM KCl, 1mM MgCl_2_, pH 7.5), MESG substrate (0.2mM final), and PNP (1 U/mL final). Absorbance at 360 nm was monitored in real time for 3 min at 22°C. Phosphate release was quantified from the change in absorbance using the extinction coefficient of 2-amino-6-mercapto-7-methylpurine (ε = 11,000 M^−1^cm^−1^). To minimize phosphate contamination, all solutions were prepared with P_i_-free water, and cuvettes were thoroughly rinsed with deionized water between measurements. For DARPin assays, purified LRRK2 protein (1μM final) was incubated with or without individual DARPins (G10 or C12; 1.2μM final) for 30 min at room temperature prior to measurement. For Rab8a Q67L–dependent assays, LRRK2 (1μM) was pre-incubated with G10 or C12 (1.2μM) for 30 min, followed by the addition of Rab8a Q67L (20μM). After an additional 10 min incubation, GTP hydrolysis rates were measured as described above.

#### In vitro guanine nucleotide exchange assay

Nucleotide exchange on LRRK2 was measured using a fluorescence-based assay as described previously.^52^ Recombinant LRRK2 protein (1μM) was preloaded with 1mM BODIPY-FL–GDP (G22360, Thermo Fisher Scientific) in exchange buffer containing 20mM HEPES (pH 7.2), 150mM KCl, 5% glycerol, and 1mM DTT. The loading reaction was performed at 4 °C for 5 h. Nucleotide loading was terminated by the addition of MgCl_2_ to a final concentration of 10mM.

To initiate nucleotide exchange, BODIPY-FL–GDP–loaded LRRK2 was rapidly mixed with C12 (1.2μM) and/or Rab8A Q67L (20μM) in exchange buffer supplemented with 5mM GTP. Reactions were carried out in black 384-well plates. Fluorescence signals were monitored in real time using a microplate reader (Cytation 5, Agilent BioTek) with excitation at 488 nm and emission at 535 nm. Measurements were recorded every 10 s for 6 min.

#### Analysis of phosphorylated Rab8a in cells

For in-cell LRRK2 activity assays, 1.2×10^6^ 293T cells were plated into each well in six well plates and incubated at 37 °C in 5% CO2. After two days, cells were transfected with 500ng GFP-Rab8a (EGFP C1 vector backbone; Addgene; RRID: Addgene_49543) plus or minus 500ng 8xHis-DARPin C12–3xFlag (pcDNA3.1 vector backbone) and 1000ng of full-length LRRK2 wild type, G2019S, R1441H, or the double mutant G2019S+R1441H (all LRRK2 in the pCDNA5 vector backbone) using polyethylenimine (Polysciences, catalog # 23966–1). After 48 h, control cells not transfected with DARPin C12 were treated with either DMSO or MLi-2 (Cayman Chemical Company, catalog #19305; 5μM) and incubated for an hour. Cells were washed three times in cold DPBS then lysed in 300 uL cold RIPA buffer per well (50mM Tris pH8, 150mM NaCl, 1% Triton X-100, 0.1% SDS, 0.5% Sodium Deoxycholate, 1mM DTT in the presence of cOmplete mini EASYpack protease inhibitor cocktail and the PhosSTOP EASYpack phosphatase inhibitor cocktail (Sigma-Aldrich, catalog # 05056489001; Roche, 04906837001)). Cell lysates were vortexed twice for 30 s then rotated for 15 min at 4°C. Lysates were clarified by centrifugation at 17,000g for 15 min at 4^◦^C, and the supernatant was moved to a new tube. SDS-PAGE loading buffer and reducing agent (1X, Invitrogen, catalog # NP0007 and NP0009) were added to the supernatant which was then boiled at 95^◦^C for 10 min. Samples were spun again at 13,000g for 5 min, and supernatant was moved to a new tube. Samples were either used immediately or stored at −20°C. For immunoblotting, samples were processed as described previously.^27^ In short, the membrane was cut at the 70kDa molecular weight marker and the top portion was immunoblotted for LRRK2 with a specific antibody (1:1000, MJFF2 (c41–2); Abcam catalog # ab133474, Lot # GR3445288–3; RRID:AB_2713963) in 1% BSA. The lower portion was immunoblotted using antibodies for phospho-Rab8a (pT72) (1:1000, Abcam catalog # 230260, Lot # GR3216587–1;RRID:AB_2814988), GFP (1:2500, Santa Cruz Biotechnology catalog # sc-9996, Lot # C2523; RRID:AB_627695), and GAPDH (1:3000, Cell Signaling Technology catalog # 2118, Lot # 16; RRID:AB_561053) in 1% milk. To visualize DARPin C12, cell lysate was reduced in Tricine SDS sample buffer (Invitrogen, catalog # LC167). Samples were run on Tricine 10%–20% gels (Invitrogen, catalog # EC66252BOX) and run in Tricine running buffer (Invitrogen, catalog # LC1675) for 1 h and 20 mins at 125V. Protein was then transferred onto polyvinylidene fluoride (PVDF) transfer membrane (0.2 μm pore size) for 75 min at 300 mA at 4 °C. Samples were then blocked in 5% milk for 1 h and incubated for 16 h at 4 °C in primary rabbit anti-FLAG polyclonal antibody (ptg labs, catalog # 20543–1-AP, Lot # 00106090; RRID:AB_11232216) in 1% milk. Samples were washed three times in Tris buffered saline buffer with 0.05% Tween-20 (TBS-T) for 10 min then incubated with goat anti-rabbit Licor680 antibody (LI-COR Biosciences; catalog # 926–32211, Lot # D40109–05; RRID:AB_621843 ) in 4% milk for 1 h at room temperature. All membranes were imaged using the LI-COR imaging system. Intensities of phospho-Rab8a, GFP, and LRRK2 bands were measured using ImageJ (version 2.14.0). Two-way ANOVA were conducted on the ratios of phosphorylated Rab8a to total Rab8a and LRRK2, normalized to the mean of the wild type LRRK2 plus DMSO group using GraphPad Prism (version 10.5.0, RRID:SCR_002798^44^) with Tukey’s multiple comparison correction.

#### RMSD analysis

The RMSD values reported in Figure S9 were obtained by doing pairwise comparisons between the ROC-COR modules of the structures shown in the figure. Specifically, we extracted the part of each model corresponding to residues 1332–1878 (ROC, COR-A, and COR-B). For each pairwise comparison, models were aligned to each other in ChimeraX^48^ (“match”), using only their backbones. Once aligned, the RMSD for the entire ROC-COR module was calculated in ChimeraX^48^ (“rmsd”), also using only the backbone atoms.

#### Figure preparation

All structural representations were prepared using ChimeraX.^48^

### QUANTIFICATION AND STATISTICAL ANALYSIS

Statistical tests are noted in figure legends. All data are shown as means ± s.d. unless otherwise stated, and all analyses were performed using Prism 10 software (GraphPad).

## Supplementary Material

1

2

3

4

SUPPLEMENTAL INFORMATION

Supplemental information can be found online at https://doi.org/10.1016/j.cell.2026.07.027.

## Figures and Tables

**Figure 1.**
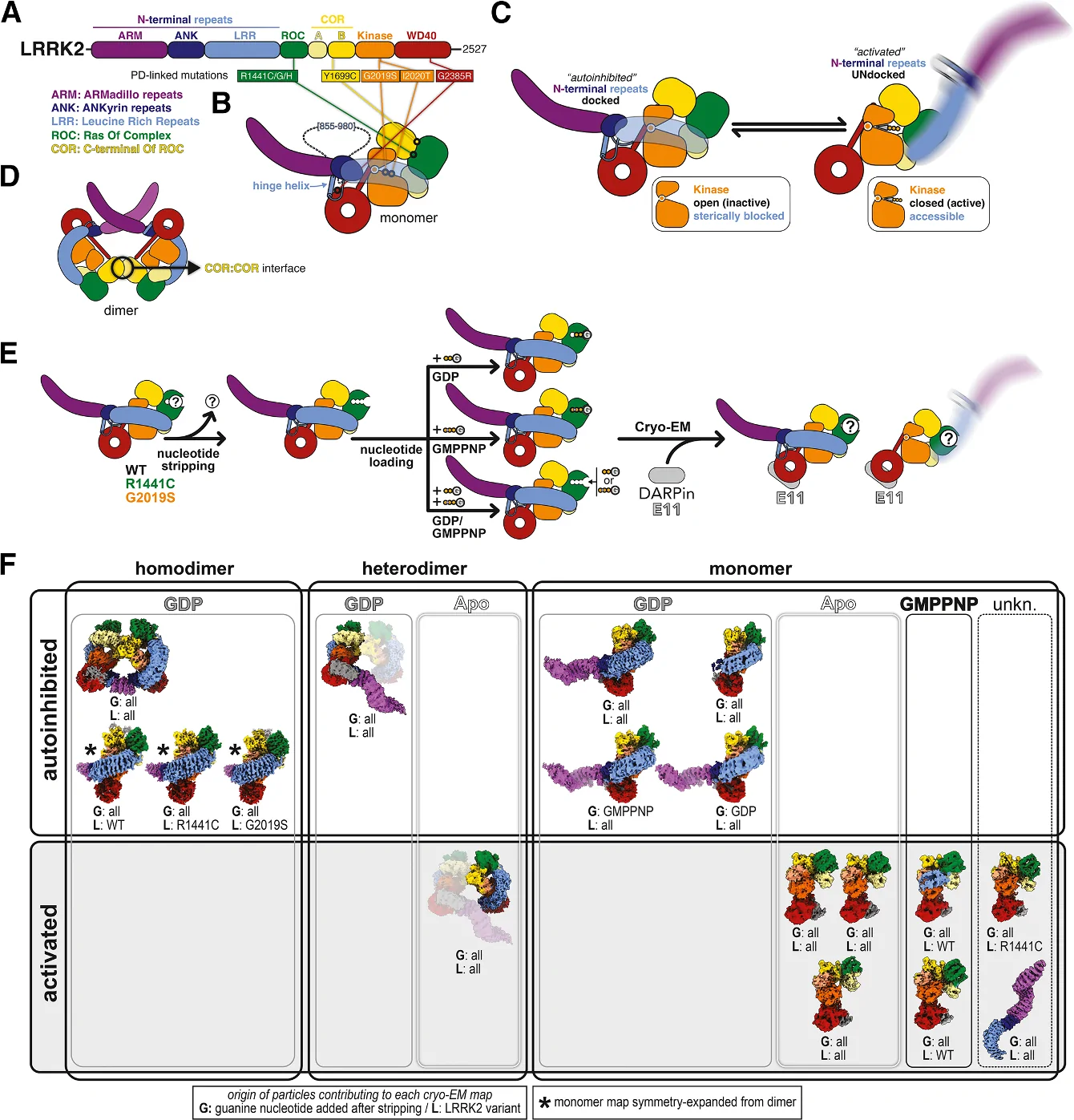
Cryo-EM reveals that GDP loss from the GTPase domain leads to LRRK2 activation (A and B) Cartoon representations of the domain architecture (A) and structure of the LRRK2 monomer (B). These cartoons represent the autoinhibited conformation of LRRK2. Common PD-linked mutations are indicated in (A) and (B). We show the LRR as semi-transparent to make the active site of the kinase visible and use the same depiction in (C). (C) Cartoon representation of the autoinhibited and activated forms of LRRK2; this nomenclature is used throughout this work. Note that although the cartoon for activated LRRK2 here shows the kinase in its closed or active conformation, we use activated in the text to indicate only the conformation of the N-terminal repeats (i.e., undocked) regardless of the conformation of the kinase. (D) Cartoon representation of the LRRK2 dimer, mediated by COR:COR interactions. (E) Schematic representation of the strategy we used for our cryo-EM analysis: LRRK2 [WT, R1441C, or G2019S] was stripped of its nucleotide and then incubated with GDP, GMPPNP, or a mixture of both. Before preparing cryo-EM grids, samples were incubated with DARPin E11, which binds to the WD40 domain and reduces the preferred orientation of LRRK2 on cryo-EM grids.^9^ Note that E11 is omitted from all cartoons of LRRK2 going forward for simplicity, although it is present in the cryo-EM maps. Data was collected from individual samples, but all data sets were merged and processed together initially, with sorting done after initial structures had been obtained and refined (see STAR Methods and Figures S1–S7 for the data processing pipeline). (F) Table of all cryo-EM structures of LRRK2 obtained in this study for which models were built, as well as a map for the N-terminal repeats, for which no model was built. Structures are sorted according to whether they represent an autoinhibited or activated form of LRRK2 by the nucleotide state we determined for their GTPase domains (Apo, GDP, GMPPNP, or unknown) and whether they are dimeric or monomeric. For the heterodimers, the highlighted monomer is the one to which the descriptors apply. We indicate, below each map, what particles contributed to it based on the guanine nucleotide added to the sample (“G”) and the LRRK2 variant (“L”) used.

**Figure 2.**
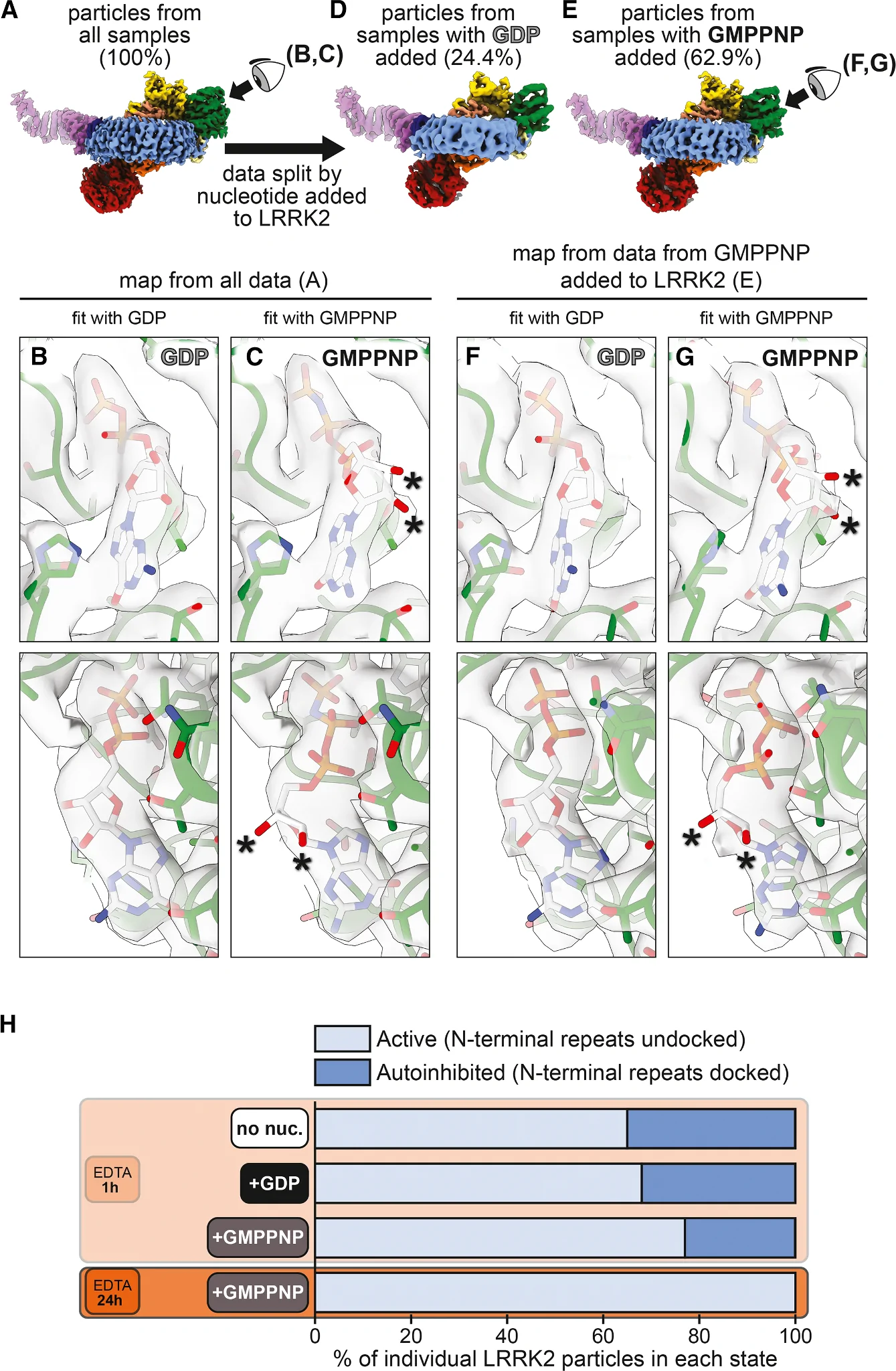
Autoinhibited LRRK2 is bound to GDP (A) Cryo-EM map of autoinhibited LRRK2 comprised of particles coming from all samples (i.e., not sorted based on the nature of the samples). (B–E) Close-ups of the nucleotide-binding pocket for the map in (A) with the model built and refined containing either GDP (B), or GMPPNP (C). The asterisks mark the 2′-OH and 3′-OH of the ribose in GMPPNP, as these are the features whose fit in the map differs most between the two nucleotides. Once we determined that the map in (A) was composed of particles coming from samples treated with GDP (24.4%), GMPPNP (62.9%), and GDP + GMPPNP (12.7%), we reconstructed maps containing particles arising from samples treated only with GDP (D) or GMPPNP (E). (F and G) Close-ups of the nucleotide-binding pocket for the map in (E) with the model built and refined containing either GDP (F), or GMPPNP (G). As in (B) and (C), asterisks mark the 2′-OH and 3′-OH of the ribose in GMPPNP. (H) Distribution of LRRK2 particles (cryo-EM data) into active and autoinhibited conformations as a function of the length of the nucleotide-stripping treatment (EDTA) and nucleotide added after it. (See Figure S8 for data processing pipeline).

**Figure 3.**
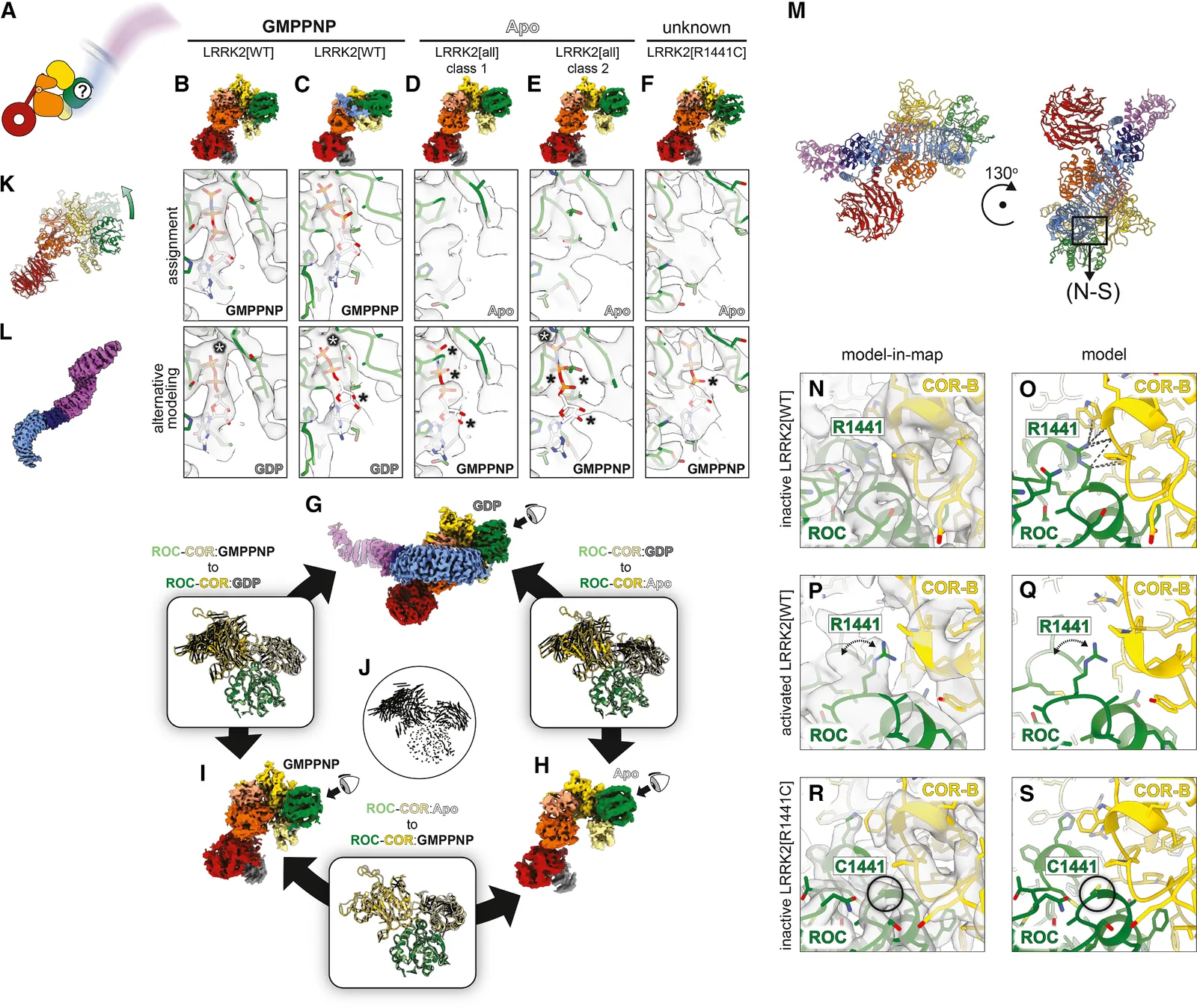
Activated LRRK2 is bound to GMPPNP or lacks a nucleotide (A) Schematic representation of activated LRRK2, with its repeats undocked. (B–F) Guanine nucleotide states of the ROC domains of activated LRRK2. Maps are shown for 5 different forms of activated LRRK2. Below the maps, we show close-ups of the nucleotide-binding pocket of the ROC domain. The top row shows the nucleotide assignments we made based on the analysis of the structures. The bottom row shows an alternative modeling to highlight the reasons why it was rejected. The asterisks highlight problems with the fits: black asterisks mark portions of the nucleotide that do not fit inside the density, while white asterisks mark parts of the map that are not accounted for by the nucleotide or come too close to it. The models on the top row were refined in the presence of GMPPNP (B) and (C) or absence of nucleotide (D)–(F). The images on the bottom row show the same maps and protein models as in the top row, with the alternative nucleotide modeled in by aligning the structure that contains it to the one shown in the image and then displaying only the nucleotide. For images labeled “GDP,” we aligned the ROC domain from LRRK2:G10:GDP (Figure 5E). For those labeled “GMPPNP,” we aligned the structure of LRRK2[WT]:GMPPNP (this figure, B). (G–J) Conformational changes in ROC-COR between different nucleotide states of LRRK2. We aligned the ROC-COR modules of autoinhibited LRRK2:GDP (G), activated LRRK2:Apo (H), and activated LRRK2:GMPPNP (I) by their GTPase domains. The images between the structures show the ROC-COR modules from the two structures along with vectors, in black, connecting equivalent alpha carbons between the structures (shown by themselves as an example in J). (K) Superposition of the model for activated LRRK2 with partial LRR density (C), and the C-terminal half from autoinhibited LRRK2 (LRRK2:G10:GDP, Figure 5E) to highlight that the former is in the activated conformation. The autoinhibited structure is shown in dim colors, and the green arrow shows the different positions of the ROC-COR module in the two models. (L) Cryo-EM map, colored by domain, of the undocked N-terminal repeats of LRRK2. The images contributing to this map originated from the same particles that gave rise to the maps of the C-terminal half of LRRK2 shown in this figure. (M–S) R1441 is involved in stabilizing the ROC:COR-B interface (the area within the box in M). (N and O) Model-in-map (N) and model alone (O) for close-ups of the ROC:COR-B interface in autoinhibited LRRK2 (the LRRK2:G10:GDP structure) showing the interactions between R1441 and COR-B. (P and Q) The same interface in the activated form of LRRK2[WT]:GMPPNP. Because the conformational changes in ROC-COR in activated LRRK2 involve a movement of COR-B away from ROC, R1441 no longer interacts with COR-B. The cryo-EM map of activated LRRK2[WT]:GMPPNP (P) shows density for two conformations of R1441, neither of which interacts with COR-B. The dashed arrow indicates the two conformations. (R and S) Model-in-map and model alone for activated LRRK2[R1441C] showing the R1441C mutation. A comparison with (N) and (O) shows how the stabilizing interaction between ROC and COR-B in autoinhibited LRRK2 cannot happen in the R1441C mutant.

**Figure 4.**
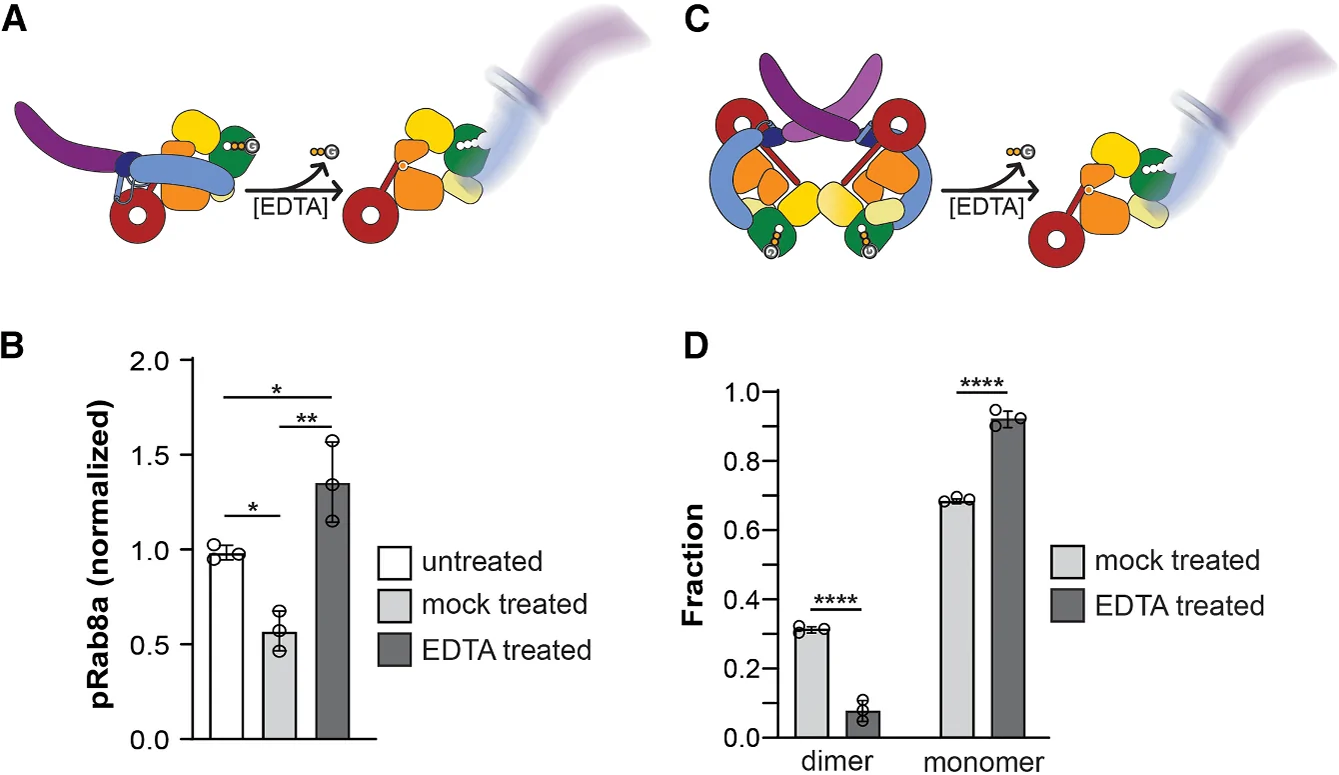
Removal of GDP activates LRRK2 and disrupts the dimer form (A) Schematic representation of the hypothesis being tested: that removal of GDP by exposing LRRK2 to the nucleotide-stripping protocol leads to its activation. (B) LRRK2[WT] was incubated with (“EDTA treated”) or without EDTA (“mock treated”) for 24 h followed by addition of 20-mM MgCl_2_ and further incubation for 24 h. Untreated LRRK2 (“untreated”) was used immediately upon thawing from frozen aliquots. All LRRK2 samples were incubated with Rab8a in the presence of ATP, with one negative control lacking ATP. Background-corrected pRab8a was normalized to the average pRab8a in the untreated LRRK2 + ATP condition from (*n* = 3) independent western blots. Images were processed in ImageJ. All data were analyzed using a one-way analysis of variance (ANOVA) with Tukey’s multiple comparison correction. *p = 0.0246 for untreated vs. mock; *p = 0.0378 for untreated vs. EDTA; **p = 0.0011 for mock vs. EDTA. Error bars ± SD. See Figure S10D for representative immunoblot. (C) Schematic representation of the hypothesis being tested: that removal of GDP by exposing LRRK2 to the nucleotide-stripping protocol destabilizes the dimeric form. (D) Fraction of LRRK2 monomers and dimers relative to (monomer + dimer) for LRRK2 exposed to the nucleotide-stripping protocol or mocked-stripped (see STAR Methods). Total particle counts corresponding to the molecular weights of either monomer or dimer were determined from mass photometry traces. The fraction of dimers contributing to the total (monomer + dimer) number of LRRK2 landing events was analyzed with one-way ANOVA and corrected for multiple comparisons using Tukey’s test. Individual data points represent 3 technical repeats. ****p < 0.0001. Error bars ± SD. See Figure S16A for mass photometry traces and particle counts.

**Figure 5.**
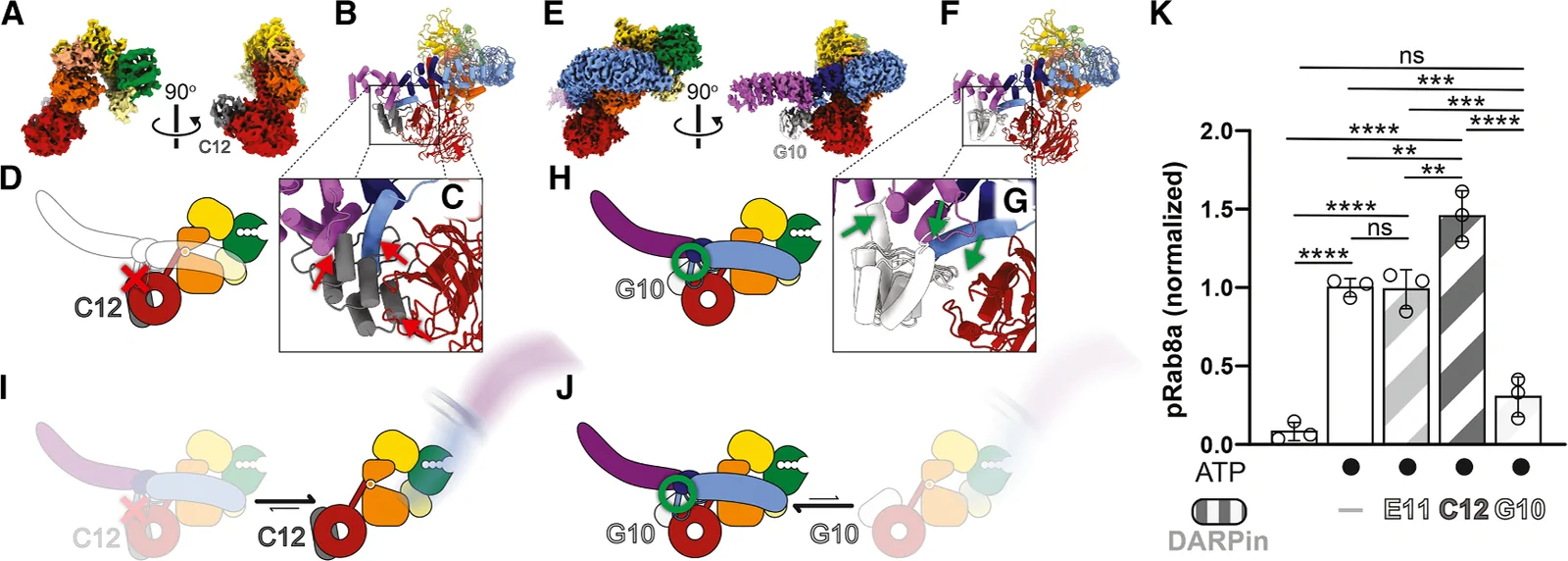
Cryo-EM structures of LRRK2^RCKW^ and LRRK2 bound to DARPins C12 and G10 (A) Cryo-EM map of LRRK2^RCKW^ with DARPin C12 bound to its WD40 domain. (B and C) Model of full-length LRRK2 bound to C12 showing the clashes that would result between the N-terminal repeats and C12 (B), and close-up of those clashes (C). (D) Cartoon illustration of the clashes. (E) Cryo-EM map of LRRK2 with DARPin G10 bound to the WD40 domain. (F and G) Structure of LRRK2 bound to G10 showing that G10 interacts both with the WD40 domain and the N-terminal repeats (F), and close-up of the interactions (G). (H) Cartoon illustration of the interactions. (I) DARPin C12 is predicted to activate LRRK2 by promoting the conformation with the N-terminal repeats undocked. (J) DARPin G10 is predicted to inhibit LRRK2 by stabilizing the conformation with the N-terminal repeats docked. (K) *In vitro* Rab8a phosphorylation assays. LRRK2[WT] was incubated with Rab8a in the presence of ATP (with a no ATP control) plus one of the following: no DARPin, E11, C12, or G10. Background-corrected pRab8a was normalized to the average of pRab8a in the LRRK2 + ATP condition from (*n* = 3) independent western blots. Images were processed in ImageJ; all data were analyzed using a one-way ANOVA with Tukey’s multiple comparison correction. *p* = non-significant for no ATP vs. LRRK2 + G10, all other comparisons with no ATP ****p < 0.0001. LRRK2 vs. LRRK2 + E11 were non-significant; **p = 0.0042 for LRRK2 vs. LRRK2 + C12; ***p = 0.0001 for LRRK2 vs. LRRK2 + G10; **p = 0.0036 for LRRK2 + E11 vs. LRRK2 + C12; ***p = 0.0002 for LRRK2 + E11 vs. LRRK2 + G10; and ****p < 0.0001 for LRRK2 + C12 vs. LRRK2 + G10. Error bars ± SD. See Figure S15A for representative immunoblot.

**Figure 6.**
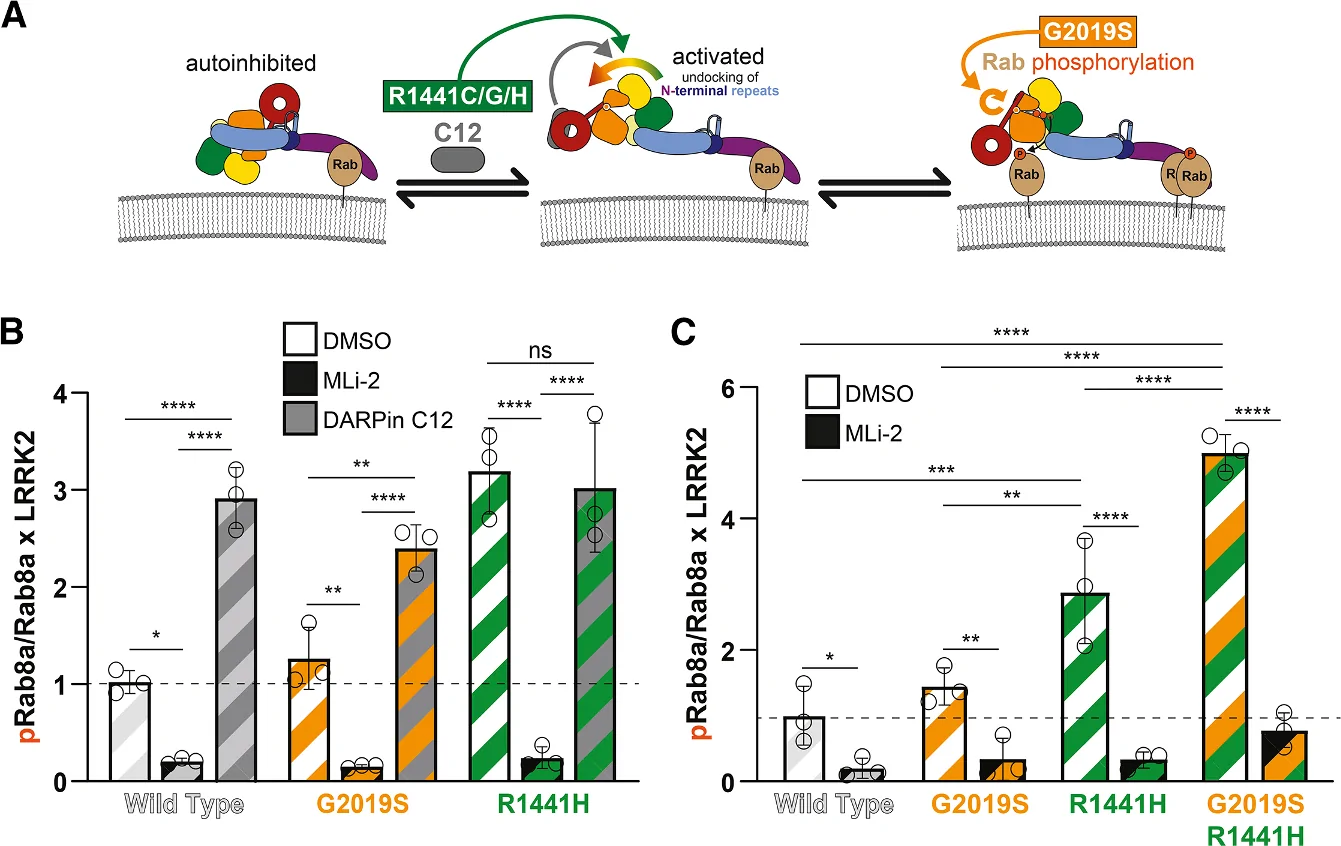
The PD mutants R1441C/G/H and G2019S increase Rab phosphorylation by different mechanisms (A) Proposed mechanism of activation of LRRK2 by the PD-linked mutations R1441C/G/H and G2019S. R1441C/G/H act by promoting the release of autoinhibition, while G2019S increases the activity of the kinase itself. Like R1441C/G/H, DARPin C12 also promotes the release of autoinhibition. (B) Quantification of pRab8a (pT72)/Rab8a × LRRK2, normalized to the mean of LRRK2[WT] + DMSO condition from (*n* = 3) independent western blots. HEK 293T co-expressing GFP-11-tagged LRRK2[WT], LRRK2[G2019S], or LRRK2[R1441H] and GFP-Rab8a with or without FLAG-tagged DARPin C12 were treated with either DMSO, MLi-2, or nothing in the C12 condition. Samples were immunoblotted for LRRK2, phospho-Rab8a (pT72), GFP, and FLAG. Images were processed on ImageJ, and all data were analyzed using a two-way ANOVA with Tukey’s multiple comparison correction. ****p < 0.0001 for WT + C12 vs. DMSO and MLi-2 and *p = 0.0161 for WT + DMSO vs. MLi-2; **p = 0.0011 and ****p < 0.0001 G2019S + C12 vs. DMSO and MLi-2; **p = 0.0013 G2019S + DMSO vs. MLi-2. ****p < 0.0001 R1441H + MLi-2 vs. C12 and DMSO; non-significant R1441H DMSO vs. C12. Error bars ± SD. (C) Quantification of pRab8a (pT72)/Rab8a x LRRK2 normalized to the mean of the LRRK2[WT] + DMSO condition. HEK 293T co-expressing LRRK2[WT], LRRK2[G2019S], and LRRK2[R1441H] or the double-mutant LRRK2[G2019S/R1441H] and GFP-Rab8a were treated, processed and analyzed as before (B). DMSO vs. MLi-2: *p = 0.0229 WT, **p = 0.0031 G2019S, and ***p < 0.0001 R1441H and DM; non-significant G2019S + DMSO vs. WT + DMSO; **p = 0.0016 G2019S + DMSO vs. R1441H + DMSO; ***p = 0.0001 R1441H + DMSO vs. WT + DMSO; ****p < 0.0001 all comparisons with the G2019S/R1441H double mutant. Error bars ± SD.

**Figure 7.**
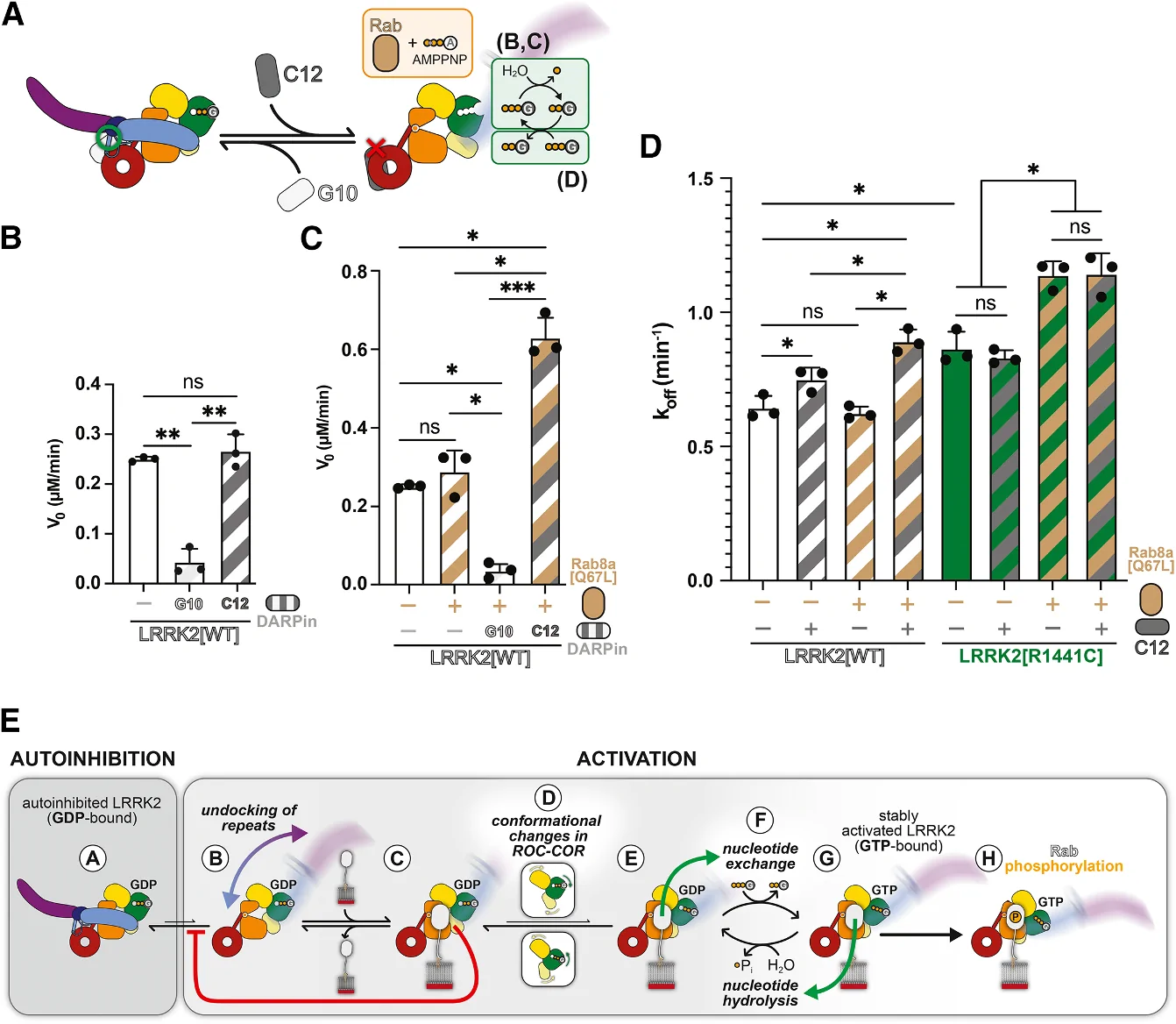
The conformation of the N-terminal repeats regulates LRRK2’s GTPase (A) Schematic of the GTPase and GDP release assays. LRRK2’s rate of GTP hydrolysis (B) and (C) or GDP release (D) were measured for LRRK2 alone or with the addition of DARPin C12 or G10 and/or the substrate Rab8a (the GTP-locked mutant Q67L). DARPin C12 promotes the activated form of LRRK2, while G10 stabilizes the autoinhibited conformation (see Figure 5). (B) Initial GTP hydrolysis velocities (V_0_, μM/min) of LRRK2-catalyzed reactions were measured in the presence of G10 or C12. Data are shown as mean ± SEM from (*n* = 3) independent experiments. Statistical analysis was performed using ANOVA with post hoc test/Student’s *t* test; **p = 0.0046 LRKK2 vs. LRRK2 + G10; 0.0011 LRRK2 + G10 vs. LRRK2 + C12; ns = not significant. (C) Initial GTP hydrolysis velocities (V_0_, μM/min) of LRRK2-catalyzed reactions were measured with Rab8 Q67L alone, or with Rab8 Q67L in combination with G10 or C12. Data are presented as mean ± SEM from (*n* = 3) independent experiments. Statistical analysis was performed using ANOVA with post hoc test/Student’s *t* test; ***p = 0.0003, *p = 0.023 LRRK2 vs. LRRK2 + Rab8a + G10; *p = 0.012 LRRK2 vs. LRRK2 + Rab8a + C12; *p = 0.011 LRRK2 + Rab8a vs. LRRK2 + Rab8a + G10; *p = 0.015 LRRK2 + Rab8a vs. LRRK2 + Rab8a + C12; and ns = not significant. (D) GDP dissociation rates (k_off_) for WT and LRRK2[R1441C]. Measurements were performed with or without DARPin C12 and with and without Rab8a[Q67L]. Data are presented as mean ± SEM from (*n* = 3) independent experiments. Statistical analysis was performed using ANOVA with post hoc test/Student’s *t* test; *p = 0.026 LRRK2[WT] vs. LRRK2[WT] + C12; *p = 0.013 LRRK2[WT] vs. LRRK2[WT] + Rab8a + C12; *p = 0.019 LRRK2[WT] vs. LRRK2[R1441C]; *p = 0.025 LRRK2[WT] + C12 vs. LRRK2[WT] + Rab8a + C12; *p = 0.017 LRRK2[WT] + Rab8a vs. LRRK2[WT] + Rab8a + C12; *p = 0.024 LRRK2[R1441C] vs. LRRK2[R1441C] + Rab8a; *p = 0.027 LRRK2[R1441C] vs. LRRK2[R1441C] + Rab8a + C12; *p = 0.021 LRRK2[R1441C] + C12 vs. LRRK2[R1441C] + Rab8a; *p = 0.026 LRRK2[R1441C] + C12 vs. LRRK2[R1441C] + Rab8a + C12; ns = not significant. (E) Schematic representation of our model for how LRRK2’s GTPase controls its activation and autoinhibition. (a) Autoinhibited LRRK2 is in the GDP-bound state. (b) The N-terminal repeats can occasionally undock, but this state is disfavored in the GDP-bound state. (c) Binding of a Rab substrate to LRRK2 sterically prevents redocking of the N-terminal repeats (red arrow), thus transiently stabilizing the undocked conformation. Once undocking of the N-terminal repeats relieves the conformational constraints on the ROC-COR module (d), the latter can switch to the active conformation (e). This conformational change in ROC-COR lowers the affinity for GDP in the ROC GTPase, leading to GDP release and binding of GTP, which is more abundant in the cell (f). The Rab substrate stimulates the release of GDP (green arrow in e). Binding of GTP stabilizes the undocked conformation of LRRK2 (g). The Rab substrate also stimulates GTP hydrolysis (green arrow in g), which returns LRRK2 to the GDP-bound state and would allow the redocking of the N-terminal repeats if a Rab substrate is not present to sterically prevent the redocking of the N-terminal repeats. The undocked, GTP-bound form of LRRK2 is responsible for the bulk of the Rab phosphorylation (h).

**KEY RESOURCES TABLE:** 

REAGENT or RESOURCE	SOURCE	IDENTIFIER

Antibodies		

LRRK2 MJFF (c41–2)	Abcam	Cat# ab133474; Lot# GR3445288–3; RRID: AB_2713963
6*His Monoclonal antibody	Proteintech	Cat# 66005; Lot# 10027680; RRID: AB_3086567
Phospho-Rab8a (pT72)	Abcam	Cat# 230260, Lot# GR3216587–1; RRID:AB_2814988
GFP	Santa Cruz Biotechnology	Cat# sc-9996; Lot# C2523; RRID:AB_627695
GAPDH	Cell Signaling Technology	Cat# 2118; Lot# 16; RRID:AB_561053
Flag	PGT Labs	Cat# 20543-1-AP, Lot# 00106090; RRID:AB_11232216
IRDye 680RD Goat Anti-Rabbit	LI-COR Biosciences	Cat# 926-68071, Lot# D50729-04; RRID: AB_2721181
IRDye 800CW Donkey Anti-Mouse	LI-COR Biosciences	Cat# 926-32212; Lot# D40820-06; RRID: AB_2716622

Bacterial and virus strains		

BL21(DE3) Competent E. coli	NEB	Cat# C2527H

Chemicals, peptides, and recombinant proteins		

Polyethylenimine	Polysciences	Cat# 23966-1
MLi-2	Cayman Chemical Company	Cat# 19305
MLi-2	Tocris	Cat# 5756
DMSO	ThermoScientific	Cat# D12345
GTP	Thermo Scientific	Cat# J61414.ME
AMPPNP	Roche	Cat# 10102547001
GMPPNP	Abcam	Cat# AB146660
DDM	GOLDBIO	Cat# DDM5_69227
BODIPY^™^ FL GDP	Invitrogen	Cat# G22360

Critical commercial assays		

EnzChek^™^ Phosphate Assay Kit	Invitrogen	Cat# E6646

Deposited data		

LRRK2	Myasnikov et al.^10^	PDB:7LHW
MLi-2-bound LRRK2^RCKW^	Sanz-Murillo et al.^17^	PDB:8TXZ
LRRK2^RCKW^	Leschziner et al.^11^	PDB:6VP7
LRRK2:GDP Dimer	Myasnikov et al.^10^	PDB:7LHT
LRRK2:14-3-3	Martinez Fiesco et al.^20^	PDB:9CI3
LRRK2:G10 DARPin	This paper	PDB:9OXH
LRRK2 with GMPPNP (active)	This paper	PDB:9Y7I
LRRK2 with GMPPNP (inactive)	This paper	PDB:9Y7A
R1441C LRRK2 (active)	This paper	PDB:9YCD
LRRK2 autoinhibited (flexible)	This paper	PDB:9OXI
LRRK2 after symmetry expansion	This paper	PDB:9Y67
G2019S LRRK2 after symmetry expansion	This paper	PDB:9Y68
LRRK2 active state (APO ROC state 1)	This paper	PDB:9YC8
LRRK2 active state (APO ROC state 2)	This paper	PDB:9YBP
R1441C LRRK2 after symmetry expansion	This paper	PDB:9OYA
LRRK2 asymmetry dimer	This paper	PDB:9YBL
LRRK2 with GDP	This paper	PDB:9YGT
LRRK2^RCKW^:C12 DARPin	This paper	PDB:9YQK
Autoinhibited LRRK2	This paper	PDB:9OM2

Experimental models: Cell lines		

Human: 293T	ATCC	CRL-3216; RRID: CVCL_0063
SF9 insect cells	Gibco	Cat# 11496015; RRID:CVCL_0549

Recombinant DNA		

EGFP-Rab8	Huang et al.^53^	Addgene Plasmid #49543; RRID: Addgene_49543
pcDNA5 FRT TO GFP11-LRRK2_G2019S	Raig et al.^54^	Addgene Plasmid #231175; RRID: Addgene_231175
pcDNA5 FRT TO GFP11-LRRK2	Raig et al.^54^	Addgene Plasmid #231174; RRID: Addgene_231174
pcDNA5 FRT TO GFP11-LRRK2_R1441H	This paper	N/A
pcDNA5 FRT TO GFP11-LRRK2_ G2019S_R1441H_DM	This paper	N/A
pcDNA3.1_8xHis_C12_DARPin_FLAG	This paper	N/A
pET14b His Rab8a Q67L	Vides et al.^55^	Addgene Plasmid #186014; RRID: Addgene_186014
pET_Rab8A1-181_WT_TwinStrep	This paper	N/A
pET_Rab8A1-181_T22N	This paper	N/A
pFB-6HZB-LRRK2	This paper	N/A
pKL-6HZB-LRRK2_G2019S	This paper	N/A
pKL-6HZB-LRRK2_R1441C	This paper	N/A
pKL-6HZB-LRRK2_RCKW	This paper	N/A
pQE30-8xHis-E11-DARPin-FLAG	Dederer et al.^27^	Addgene Plasmid: #226784; RRID: Addgene_226784
pQE30-8xHis-C12-DARPin-FLAG	This paper	N/A
pQE30-8xHis-G10-DARPin-FLAG	This paper	N/A
pKL	Fitzgerald et al.^56^	Addgene Plasmid: # 110741; RRID: Addgene_110741

Software and algorithms		

ImageJ	Schindelin et al.^57^	https://imagej.nih.gov/ij/
Prism	GraphPad	https://www.graphpad.com/
CryoSPARC	Punjani et al.^46^	https://cryosparc.com/
Topaz	Bepler et al.^47^	https://cb.csail.mit.edu/topaz/
UCSF ChimeraX	Petterson et al.^48^	https://www.cgl.ucsf.edu/chimerax/
Coot	Emsley and Cowtan^50^	https://www2.mrc-lmb.cam.ac.uk/personal/pemsley/coot/
Phenix	He et al.^58^	https://phenix-online.org/
Alphafold 3	Abramson et al.^49^	https://alphafoldserver.com/welcome

Other		

cOmplete mini EASYpack protease inhibitor cocktail	Sigma-Aldrich	Cat# 05056489001
PhosSTOP EASYpack phosphatase inhibitor cocktail	Roche	Cat# 04906837001
SDS-PAGE loading buffer	Invitrogen	Cat# NP0007
Reducing Buffer	Invitrogen	Cat# NP0009
Tricine SDS sample buffer	Invitrogen	Cat# LC167
Tricine 10%–20% Protein Gels	Invitrogen	Cat# EC66252BOX
Tricine Running Buffer	Invitrogen	Cat# LC1675
NuPAGE MOPS SDS Running Buffer (20X)	Invitrogen	Cat# NP0001
UltrAuFoil Holey Gold 300 mesh R1.2/1.3 grids	Quantifoil	Cat# Q350AR13A
Nupage^™^ 4%–12% Bis-Tris Protein Gels	Invitrogen	Cat# NP0321BOX

## Highlights

- GDP binding stabilizes an autoinhibited conformation of LRRK2
- GTP binding promotes activation via conformational changes in its ROC GTPase domain
- Parkinson’s disease mutations activate LRRK2 through distinct structural mechanisms
- The GTPase nucleotide cycle provides a framework for allosteric modulation of LRRK2

## References

[1] Krüger C, Lim S-Y, Buhrmann A, Fahrig FL, Gabbert C, Bahr N, Madoev H, Marras C, Klein C, and Lohmann K (2025). Updated MDSGene review on the clinical and genetic spectrum of LRRK2 variants in Parkinson’s disease. NPJ Parkinsons Dis. 11, 30. 10.1038/s41531-025-00881-9. PMC11832785 39962078

[2] Steger M, Tonelli F, Ito G, Davies P, Trost M, Vetter M, Wachter S, Lorentzen E, Duddy G, Wilson S, (2016). Phosphoproteomics reveals that Parkinson’s disease kinase LRRK2 regulates a subset of Rab GTPases. eLife 5, e12813. 10.7554/eLife.12813. PMC4769169 26824392

[3] Steger M, Diez F, Dhekne HS, Lis P, Nirujogi RS, Karayel O, Tonelli F, Martinez TN, Lorentzen E, Pfeffer SR, (2017). Systematic proteomic analysis of LRRK2-mediated Rab GTPase phosphorylation establishes a connection to ciliogenesis. eLife 6, e31012. 10.7554/eLife.31012. PMC5695910 29125462

[4] Hutagalung AH, and Novick PJ (2011). Role of Rab GTPases in membrane traffic and cell physiology. Physiol. Rev. 91, 119–149. 10.1152/physrev.00059.2009. PMC3710122 21248164

[5] West AB, Moore DJ, Biskup S, Bugayenko A, Smith WW, Ross CA, Dawson VL, and Dawson TM (2005). Parkinson’s disease-associated mutations in leucine-rich repeat kinase 2 augment kinase activity. Proc. Natl. Acad. Sci. USA 102, 16842–16847. 10.1073/pnas.0507360102. PMC1283829 16269541

[6] Gloeckner CJ, Kinkl N, Schumacher A, Braun RJ, O’Neill E, Meitinger T, Kolch W, Prokisch H, and Ueffing M (2006). The Parkinson disease causing LRRK2 mutation I2020T is associated with increased kinase activity. Hum. Mol. Genet. 15, 223–232. 10.1093/hmg/ddi439. 16321986

[7] Kalogeropulou AF, Purlyte E, Tonelli F, Lange SM, Wightman M, Prescott AR, Padmanabhan S, Sammler E, and Alessi DR (2022). Impact of 100 LRRK2 variants linked to Parkinson’s disease on kinase activity and microtubule binding. Biochem. J. 479, 1759–1783. 10.1042/BCJ20220161. PMC9472821 35950872

[8] Di Maio R, Hoffman EK, Rocha EM, Keeney MT, Sanders LH, De Miranda BR, Zharikov A, Van Laar A, Stepan AF, Lanz TA, (2018). LRRK2 activation in idiopathic Parkinson’s disease. Sci. Transl. Med. 10, eaar5429. 10.1126/scitranslmed.aar5429. PMC6344941 30045977

[9] Tian Y, Lv J, Su Z, Wu T, Li X, Hu X, Zhang J, and Wu L (2021). LRRK2 plays essential roles in maintaining lung homeostasis and preventing the development of pulmonary fibrosis. Proc. Natl. Acad. Sci. USA 118, e2106685118. 10.1073/pnas.2106685118. PMC8536316 34446559

[10] Myasnikov A, Zhu H, Hixson P, Xie B, Yu K, Pitre A, Peng J, and Sun J (2021). Structural analysis of the full-length human LRRK2. Cell 184, 3519–3527.e10. 10.1016/j.cell.2021.05.004. PMC8887629 34107286

[11] Deniston CK, Salogiannis J, Mathea S, Snead DM, Lahiri I, Matyszewski M, Donosa O, Watanabe R, Böhning J, Shiau AK, (2020). Structure of LRRK2 in Parkinson’s disease and model for microtubule interaction. Nature 588, 344–349. 10.1038/s41586-020-2673-2. PMC7726071 32814344

[12] West AB, Moore DJ, Choi C, Andrabi SA, Li X, Dikeman D, Biskup S, Zhang Z, Lim K-L, Dawson VL, (2007). Parkinson’s disease-associated mutations in LRRK2 link enhanced GTP-binding and kinase activities to neuronal toxicity. Hum. Mol. Genet. 16, 223–232. 10.1093/hmg/ddl471. 17200152

[13] Taymans J-M, Vancraenenbroeck R, Ollikainen P, Beilina A, Lobbestael E, De Maeyer M, Baekelandt V, and Cookson MR (2011). LRRK2 kinase activity is dependent on LRRK2 GTP binding capacity but independent of LRRK2 GTP binding. PLOS One 6, e23207. 10.1371/journal.pone.0023207. PMC3155532 21858031

[14] Blanca Ramírez M, Lara Ordóñez AJ, Fdez E, Madero-Pérez J, Gonnelli A, Drouyer M, Chartier-Harlin M-C, Taymans J-M, Bubacco L, Greggio E, (2017). GTP binding regulates cellular localization of Parkinson’s disease-associated LRRK2. Hum. Mol. Genet. 26, 2747–2767. 10.1093/hmg/ddx161. PMC5886193 28453723

[15] Guo L, Gandhi PN, Wang W, Petersen RB, Wilson-Delfosse AL, and Chen SG (2007). The Parkinson’s disease-associated protein, leucine-rich repeat kinase 2 (LRRK2), is an authentic GTPase that stimulates kinase activity. Exp. Cell Res. 313, 3658–3670. 10.1016/j.yexcr.2007.07.007. PMC2083285 17706965

[16] Gilsbach BK, Ho FY, Riebenbauer B, Zhang X, Guaitoli G, Kortholt A, and Gloeckner CJ (2024). Intramolecular feedback regulation of the LRRK2 Roc G domain by a LRRK2 kinase-dependent mechanism. eLife 12, RP91083. 10.7554/eLife.91083. PMC11658767 39699947

[17] Sanz Murillo M, Villagran Suarez A, Dederer V, Chatterjee D, Alegrio Louro J, Knapp S, Mathea S, and Leschziner AE (2023). Inhibition of Parkinson’s disease-related LRRK2 by type I and type II kinase inhibitors: Activity and structures. Sci. Adv. 9, eadk6191. 10.1126/sciadv.adk6191. PMC10691770 38039358

[18] Zhu H, Hixson P, Ma W, and Sun J (2024). Pharmacology of LRRK2 with type I and II kinase inhibitors revealed by cryo-EM. Cell Discov. 10, 10. 10.1038/s41421-023-00639-8. PMC10805800 38263358

[19] Zhu H, Tonelli F, Turk M, Prescott A, Alessi DR, and Sun J (2023). Rab29-dependent asymmetrical activation of leucine-rich repeat kinase 2. Science 382, 1404–1411. 10.1126/science.adi9926. PMC10786121 38127736

[20] Martinez Fiesco JA, Beilina A, Alvarez de la Cruz A, Li N, Metcalfe RD, Cookson MR, and Zhang P. (2025). 14–3-3 binding maintains the Parkinson’s associated kinase LRRK2 in an inactive state. Nat. Commun. 16, 7226. 10.1038/s41467-025-62337-1. PMC12325948 40764514

[21] Kett LR, Boassa D, Ho CC-Y, Rideout HJ, Hu J, Terada M, Ellisman M, and Dauer WT (2012). LRRK2 Parkinson disease mutations enhance its microtubule association. Hum. Mol. Genet. 21, 890–899. 10.1093/hmg/ddr526. PMC3263991 22080837

[22] Dzamko N, Deak M, Hentati F, Reith AD, Prescott AR, Alessi DR, and Nichols RJ (2010). Inhibition of LRRK2 kinase activity leads to dephosphorylation of Ser(910)/Ser(935), disruption of 14–3–3 binding and altered cytoplasmic localization. Biochem. J. 430, 405–413. 10.1042/BJ20100784. PMC3631100 20659021

[23] Doggett EA, Zhao J, Mork CN, Hu D, and Nichols RJ (2012). Phosphorylation of LRRK2 serines 955 and 973 is disrupted by Parkinson’s disease mutations and LRRK2 pharmacological inhibition. J. Neurochem. 120, 37–45. 10.1111/j.1471-4159.2011.07537.x. 22004453

[24] Herzig MC, Kolly C, Persohn E, Theil D, Schweizer T, Hafner T, Stemmelen C, Troxler TJ, Schmid P, Danner S, (2011). LRRK2 protein levels are determined by kinase function and are crucial for kidney and lung homeostasis in mice. Hum. Mol. Genet. 20, 4209–4223. 10.1093/hmg/ddr348. PMC3188995 21828077

[25] Kalayci T, Wikenheiser-Brokamp KA, Gomes S, Rawat E, Peljto A, Cheung A, Citak S, Vayvada M, Montero-Fernandez MA, Mogulkoc N, (2026). Rare biallelic loss-of-function variants in the LRRK2 kinase cause interstitial lung disease. Preprint at medRxiv. 10.64898/2026.05.15.26352763.

[26] Lebovitz C, Wretham N, Osooly M, Milne K, Dash T, Thornton S, Tessier-Cloutier B, Sathiyaseelan P, Bortnik S, Go NE, (2021). Loss of Parkinson’s susceptibility gene LRRK2 promotes carcinogen-induced lung tumorigenesis. Sci. Rep. 11, 2097. 10.1038/s41598-021-81639-0. PMC7822882 33483550

[27] Dederer V, Sanz Murillo M, Karasmanis EP, Hatch KS, Chatterjee D, Preuss F, Abdul Azeez KR, Nguyen LV, Galicia C, Dreier B, (2024). A designed ankyrin-repeat protein that targets Parkinson’s disease-associated LRRK2. J. Biol. Chem. 300, 107469. 10.1016/j.jbc.2024.107469. PMC11284679 38876305

[28] Liu Z, Bryant N, Kumaran R, Beilina A, Abeliovich A, Cookson MR, and West AB (2018). LRRK2 phosphorylates membrane-bound Rabs and is activated by GTP-bound Rab7L1 to promote recruitment to the trans-Golgi network. Hum. Mol. Genet. 27, 385–395. 10.1093/hmg/ddx410. PMC5886198 29177506

[29] Dhekne HS, Tonelli F, Yeshaw WM, Chiang CY, Limouse C, Jaimon E, Purlyte E, Alessi DR, and Pfeffer SR (2023). Genome-wide screen reveals Rab12 GTPase as a critical activator of Parkinson’s disease-linked LRRK2 kinase. eLife 12, e87098. 10.7554/eLife.87098. PMC10708890 37874635

[30] Wang X, Bondar VV, Davis OB, Maloney MT, Agam M, Chin MY, Cheuk-Nga Ho A, Ghosh R, Leto DE, Joy D, (2023). Rab12 is a regulator of LRRK2 and its activation by damaged lysosomes. eLife 12, e87255. 10.7554/eLife.87255. PMC10708889 37874617

[31] McGrath E, Waschbüsch D, Baker BM, and Khan AR. (2021). LRRK2 binds to the Rab32 subfamily in a GTP-dependent manner via its armadillo domain. Small GTPases 12, 133–146. 10.1080/21541248.2019.1666623. PMC7849779 31552791

[32] Follett J, Deng IB, Sharp RC, Wall S, Mamais A, and Farrer MJ (2026). Peripheral inflammation mediates midbrain Lrrk2 kinase activity via Rab32 expression. Preprint at bioRxiv. 10.1101/2025.09.05.674552.

[33] Bentley-DeSousa A, Roczniak-Ferguson A, and Ferguson SM (2025). A STING-CASM-GABARAP pathway activates LRRK2 at lysosomes. J. Cell Biol. 224, e202310150. 10.1083/jcb.202310150. PMC11734622 39812709

[34] Clegg D, Bentley-DeSousa A, Roczniak-Ferguson A, and Ferguson SM (2025). LRRK2 integrates Rab and GABARAP interactions to sense and respond to distinct lysosomal stresses. Preprint at bioRxiv. 10.1101/2025.11.19.689251.

[35] Purlyte E, Dhekne HS, Sarhan AR, Gomez R, Lis P, Wightman M, Martinez TN, Tonelli F, Pfeffer SR, and Alessi DR (2018). Rab29 activation of the Parkinson’s disease-associated LRRK2 kinase. EMBO J. 37, 1–18. 10.15252/embj.201798099. PMC5753036 29212815

[36] Zhu H, Eade L, Alessi DR, and Sun J (2026). Elucidating the molecular interplay between LRRK2 and Rab GTPases. Preprint at bioRxiv. 2026.04.30.722110. 10.64898/2026.04.30.722110.

[37] Watanabe R, Buschauer R, Böhning J, Audagnotto M, Lasker K, Lu TW, Boassa D, Taylor S, and Villa E. (2020). The In Situ Structure of Parkinson’s Disease-Linked LRRK2. Cell 182, 1508–1518.e16. 10.1016/j.cell.2020.08.004. PMC7869717 32783917

[38] Snead DM, Matyszewski M, Dickey AM, Lin YX, Leschziner AE, and Reck-Peterson SL (2022). Structural basis for Parkinson’s disease-linked LRRK2’s binding to microtubules. Nat. Struct. Mol. Biol. 29, 1196–1207. 10.1038/s41594-022-00863-y. PMC9758056 36510024

[39] Ito G, Okai T, Fujino G, Takeda K, Ichijo H, Katada T, and Iwatsubo T (2007). GTP binding is essential to the protein kinase activity of LRRK2, a causative gene product for familial Parkinson’s disease. Biochemistry 46, 1380–1388. 10.1021/bi061960m. 17260967

[40] Biosa A, Trancikova A, Civiero L, Glauser L, Bubacco L, Greggio E, and Moore DJ (2013). GTPase activity regulates kinase activity and cellular phenotypes of Parkinson’s disease-associated LRRK2. Hum. Mol. Genet. 22, 1140–1156. 10.1093/hmg/dds522. 23241358

[41] Galicia C, Guaitoli G, Fislage M, Gloeckner CJ, and Versées W. (2024). Structural insights into the GTP-driven monomerization and activation of a bacterial LRRK2 homolog using allosteric nanobodies. eLife 13, RP94503. 10.7554/eLife.94503. PMC11052575 38666771

[42] Giusto E, Yacoubian TA, Greggio E, and Civiero L (2021). Pathways to Parkinson’s disease: a spotlight on 14–3-3 proteins. NPJ Parkinsons Dis. 7, 85. 10.1038/s41531-021-00230-6. PMC8455551 34548498

[43] Manschwetus JT, Wallbott M, Fachinger A, Obergruber C, Pautz S, Bertinetti D, Schmidt SH, and Herberg FW (2020). Binding of the Human 14–3-3 Isoforms to Distinct Sites in the Leucine-Rich Repeat Kinase 2. Front. Neurosci. 14, 302. 10.3389/fnins.2020.00302. PMC7155755 32317922

[44] Obsilova V, and Obsil T (2020). The 14–3-3 Proteins as Important Allosteric Regulators of Protein Kinases. Int. J. Mol. Sci. 21, 8824. 10.3390/ijms21228824. PMC7700312 33233473

[45] Obsilova V, and Obsil T (2022). Structural insights into the functional roles of 14–3–3 proteins. Front. Mol. Biosci. 9, 1016071. 10.3389/fmolb.2022.1016071. PMC9523730 36188227

[46] Punjani A, Rubinstein JL, Fleet DJ, and Brubaker MA (2017). cryoSPARC: algorithms for rapid unsupervised cryo-EM structure determination. Nat. Methods 14, 290–296. 10.1038/nmeth.4169. 28165473

[47] Bepler T, Morin A, Rapp M, Brasch J, Shapiro L, Noble AJ, and Berger B (2019). Positive-unlabeled convolutional neural networks for particle picking in cryo-electron micrographs. Nat. Methods 16, 1153–1160. 10.1038/s41592-019-0575-8. PMC6858545 31591578

[48] Pettersen EF, Goddard TD, Huang CC, Meng EC, Couch GS, Croll TI, Morris JH, and Ferrin TE (2021). UCSF ChimeraX: Structure visualization for researchers, educators, and developers. Protein Sci. 30, 70–82. 10.1002/pro.3943. PMC7737788 32881101

[49] Abramson J, Adler J, Dunger J, Evans R, Green T, Pritzel A, Ronneberger O, Willmore L, Ballard AJ, Bambrick J, (2024). Accurate structure prediction of biomolecular interactions with AlphaFold 3. Nature 630, 493–500. 10.1038/s41586-024-07487-w. PMC11168924 38718835

[50] Emsley P, and Cowtan K (2004). Coot: model-building tools for molecular graphics. Acta Crystallogr., D 60, 2126–2132. 10.1107/S0907444904019158. 15572765

[51] He J, Li T, and Huang S-Y (2023). Improvement of cryo-EM maps by simultaneous local and non-local deep learning. Nat. Commun. 14, 3217. 10.1038/s41467-023-39031-1. PMC10239474 37270635

[52] Blaise AM, Corcoran EE, Wattenberg ES, Zhang Y-L, Cottrell JR, and Koleske AJ (2022). In vitro fluorescence assay to measure GDP/GTP exchange of guanine nucleotide exchange factors of Rho family GTPases. Biol. Methods Protoc. 7, bpab024. 10.1093/biomethods/bpab024. PMC8789339 35087952

[53] Huang B, Hubber A, McDonough JA, Roy CR, Scidmore MA, and Carlyon JA (2010). The Anaplasma phagocytophilum-occupied vacuole selectively recruits Rab-GTPases that are predominantly associated with recycling endosomes. Cell. Microbiol. 12, 1292–1307. 10.1111/j.1462-5822.2010.01468.x. PMC2923681 20345488

[54] Raig ND, Surridge KJ, Sanz-Murillo M, Dederer V, Krämer A, Schwalm MP, Lattal NM, Elson L, Chatterjee D, Mathea S, (2025). Type II kinase inhibitors that target Parkinson’s disease-associated LRRK2. Sci. Adv. 11, eadt2050. 10.1126/sciadv.adt2050. PMC12136037 40465731

[55] Vides EG, Adhikari A, Chiang CY, Lis P, Purlyte E, Limouse C, Shumate JL, Spıńola-Lasso E, Dhekne HS, Alessi DR, (2022). A feed-forward pathway drives LRRK2 kinase membrane recruitment and activation. eLife 11, e79771. 10.7554/eLife.79771. PMC9576273 36149401

[56] Fitzgerald DJ, Berger P, Schaffitzel C, Yamada K, Richmond TJ, and Berger I (2006). Protein complex expression by using multigene baculoviral vectors. Nat. Methods 3, 1021–1032. 10.1038/nmeth983. 17117155

[57] Schindelin J, Arganda-Carreras I, Frise E, Kaynig V, Longair M, Pietzsch T, Preibisch S, Rueden C, Saalfeld S, Schmid B, (2012). Fiji: an open-source platform for biological-image analysis. Nat. Methods 9, 676–682. 10.1038/nmeth.2019. PMC3855844 22743772

[58] Liebschner D, Afonine PV, Baker ML, Bunkóczi G, Chen VB, Croll TI, Hintze B, Hung LW, Jain S, McCoy AJ, (2019). Macromolecular structure determination using X-rays, neutrons and electrons: recent developments in Phenix. Acta Crystallogr. D Struct. Biol. 75, 861–877. 10.1107/S2059798319011471. PMC6778852 31588918

[59] Adhikari A, Tripathi A, Chiang CY, Sherpa P, and Pfeffer SR (2025). Allosteric regulation of the Golgi-localized PPM1H phosphatase by Rab GTPases modulates LRRK2 substrate dephosphorylation in Parkinson’s disease. J. Biol. Chem. 301, 110679. 10.1016/j.jbc.2025.110679. PMC12528898 40912655

[60] Waschbüsch D, Berndsen K, Lis P, Knebel A, Lam YP, Alessi DR, and Khan AR (2021). Structural basis for the specificity of PPM1H phosphatase for Rab GTPases. EMBO Rep. 22, e52675. 10.15252/embr.202152675. PMC8567228 34580980

[61] Berndsen K, Lis P, Yeshaw WM, Wawro PS, Nirujogi RS, Wightman M, Macartney T, Dorward M, Knebel A, Tonelli F, (2019). PPM1H phosphatase counteracts LRRK2 signaling by selectively dephosphorylating Rab proteins. eLife 8, e50416. 10.7554/eLife.50416. PMC6850886 31663853

