# Supplementary material for: The structural basis for LRRK2’s activation and autoinhibition
Source: Cell. Author manuscript; Available in PMC 2026 Oct 2. (PMC13632595; doi:10.1016/j.cell.2026.07.027)
Supplement: Supplementary file 1 [file NIHMS2204259-supplement-1.pdf]

SUPPLEMENTARY FIGURES

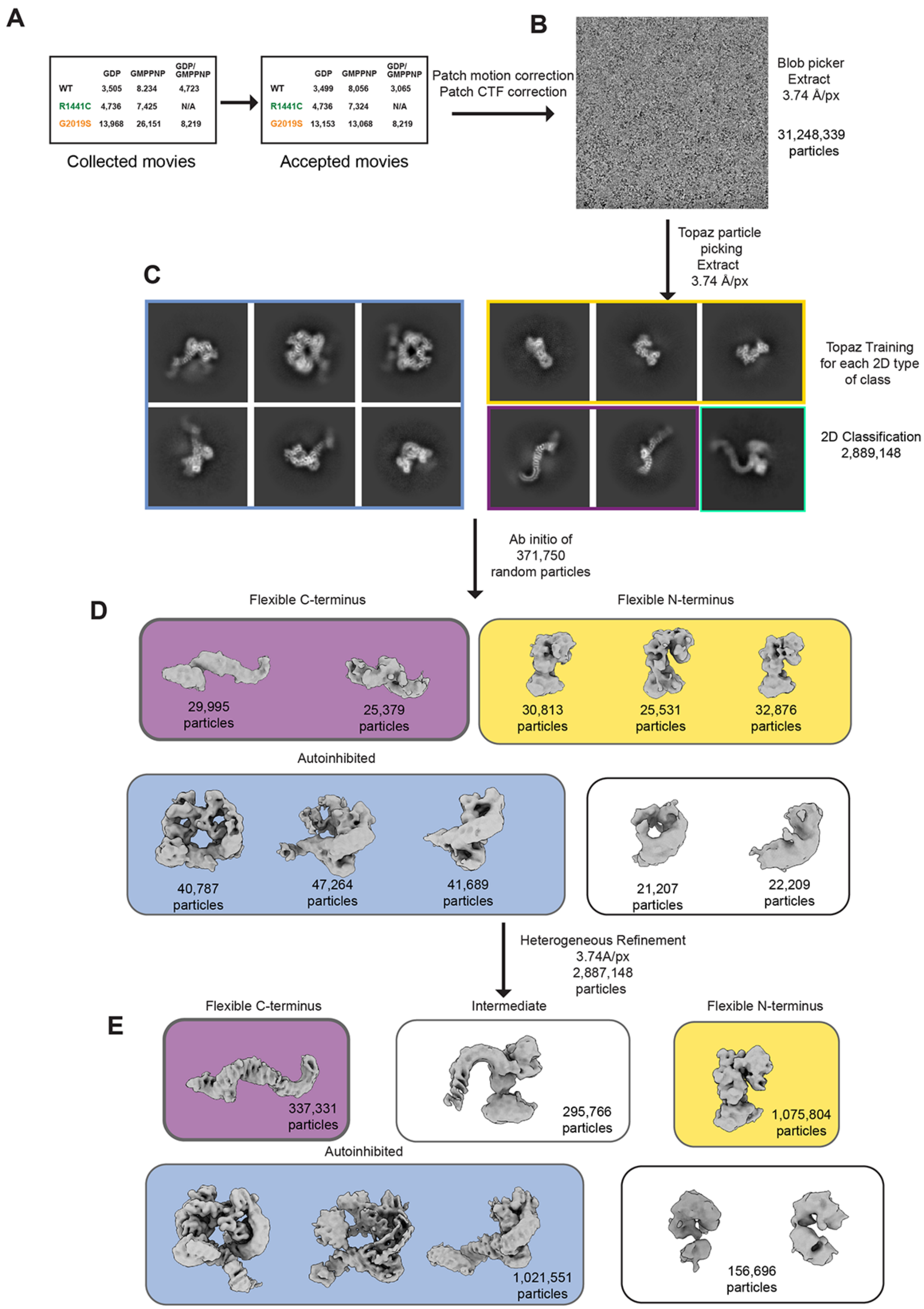

Figure S1. Overall Cryo-EM processing pipeline for the combined data set, related to Figures 1-3. (A) Number of movies that were collected and kept for further processing. **(B, C)** Representative micrograph (B) and class averages (C). **(D)** Data processing strategy. **(E)** Splitting of data into volumes representing different states/conformations of LRRK2.

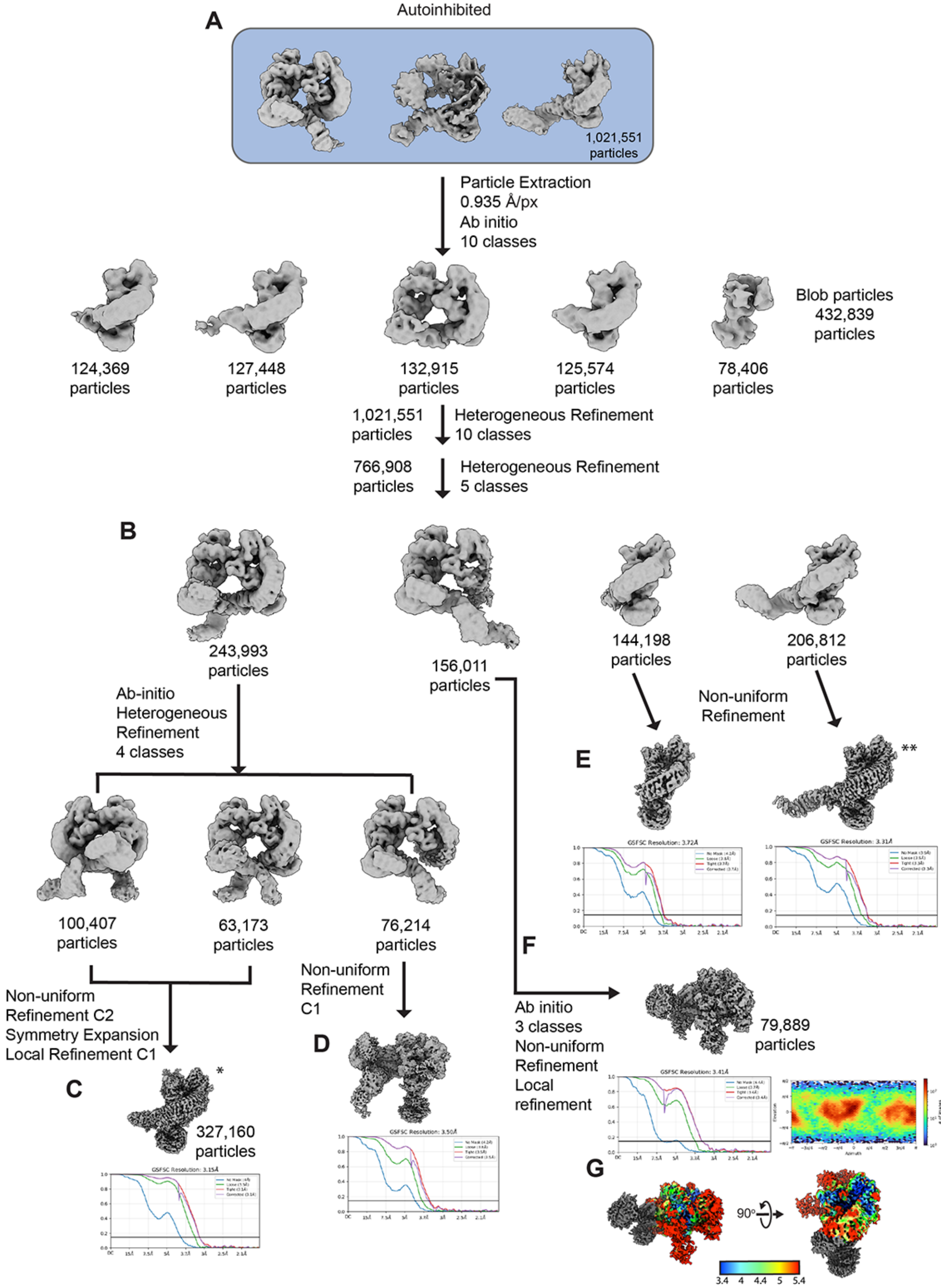

Figure S2. Cryo-EM processing pipeline for the combined data set: autoinhibited LRRK2 and mixed dimers, related to Figures 1-3. (A) Data processing strategy. **(B)** Processing of the autoinhibited dimer. **(C)** Volume and FSC curve for the dimer after symmetry expansion. **(D)** Volume and FSC curve for the asymmetric dimer (autoinhibited protomer). **(E)** Volumes and FSC curves for autoinhibited monomers. **(F)** Volume, FSC curve and Euler angle distribution for the asymmetric dimer (activated protomer). **(G)** Local resolution map. * and ** indicate volumes whose processing continues in Figure S3.

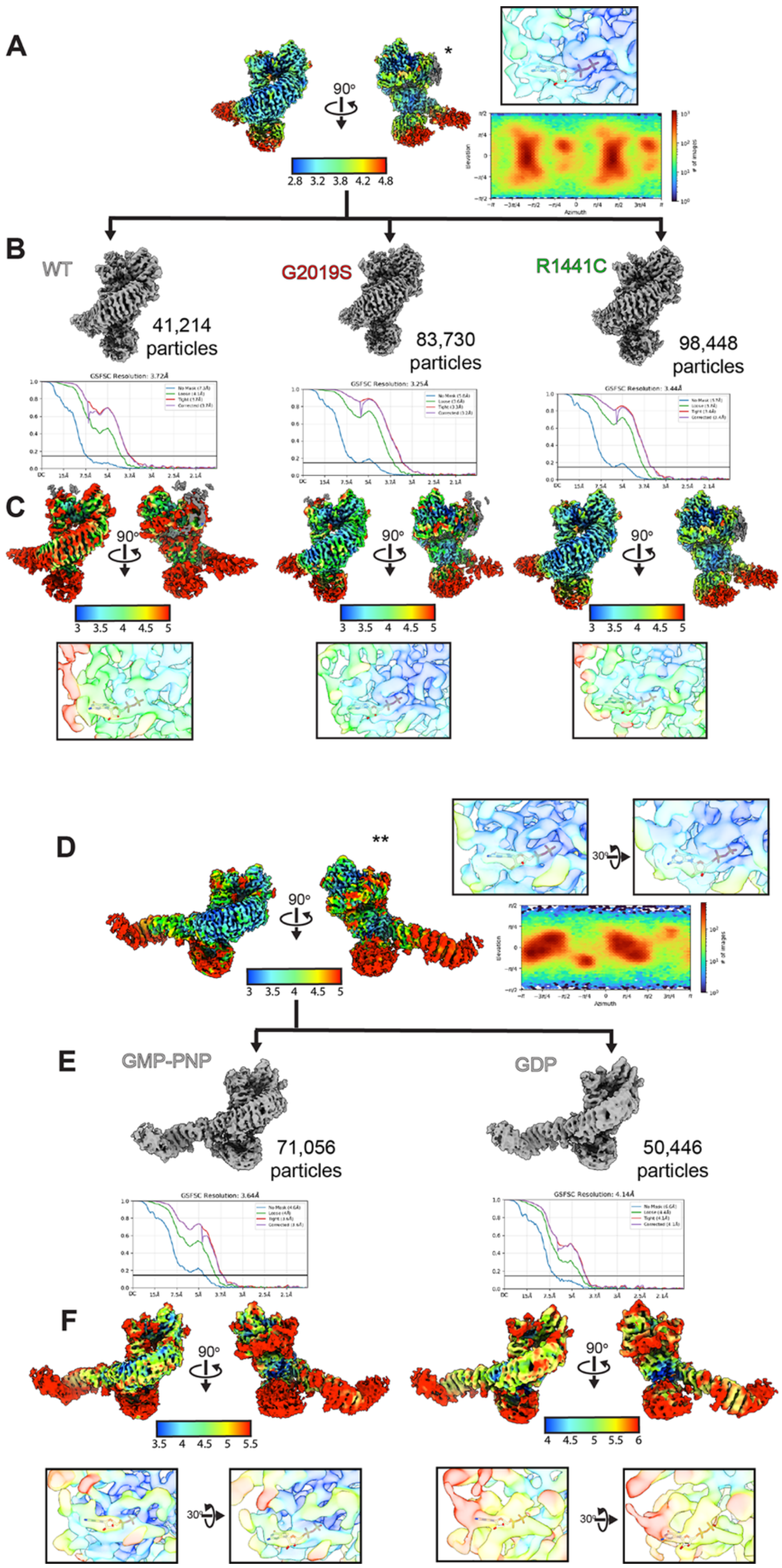

Figure S3. Cryo-EM processing pipeline for autoinhibited LRRK2 and mixed dimers: splitting by nucleotide added or by LRRK2 variant, related to Figures 1-3. (A) Local resolution map and Euler angle distribution for the dimer after symmetry expansion. **(B)** Data splitting by LRRK2 variant; volumes and FSC curves. **(C)** Local resolution map in the vicinity of the nucleotide-binding pocket of the ROC domain. **(D)** Local resolution map and Euler angle distribution for the monomer. **(E)** Data splitting by the guanine nucleotide added to the sample; volumes and FSC curves. **(F)** Local resolution map in the vicinity of the nucleotide-binding pocket of the ROC domain.

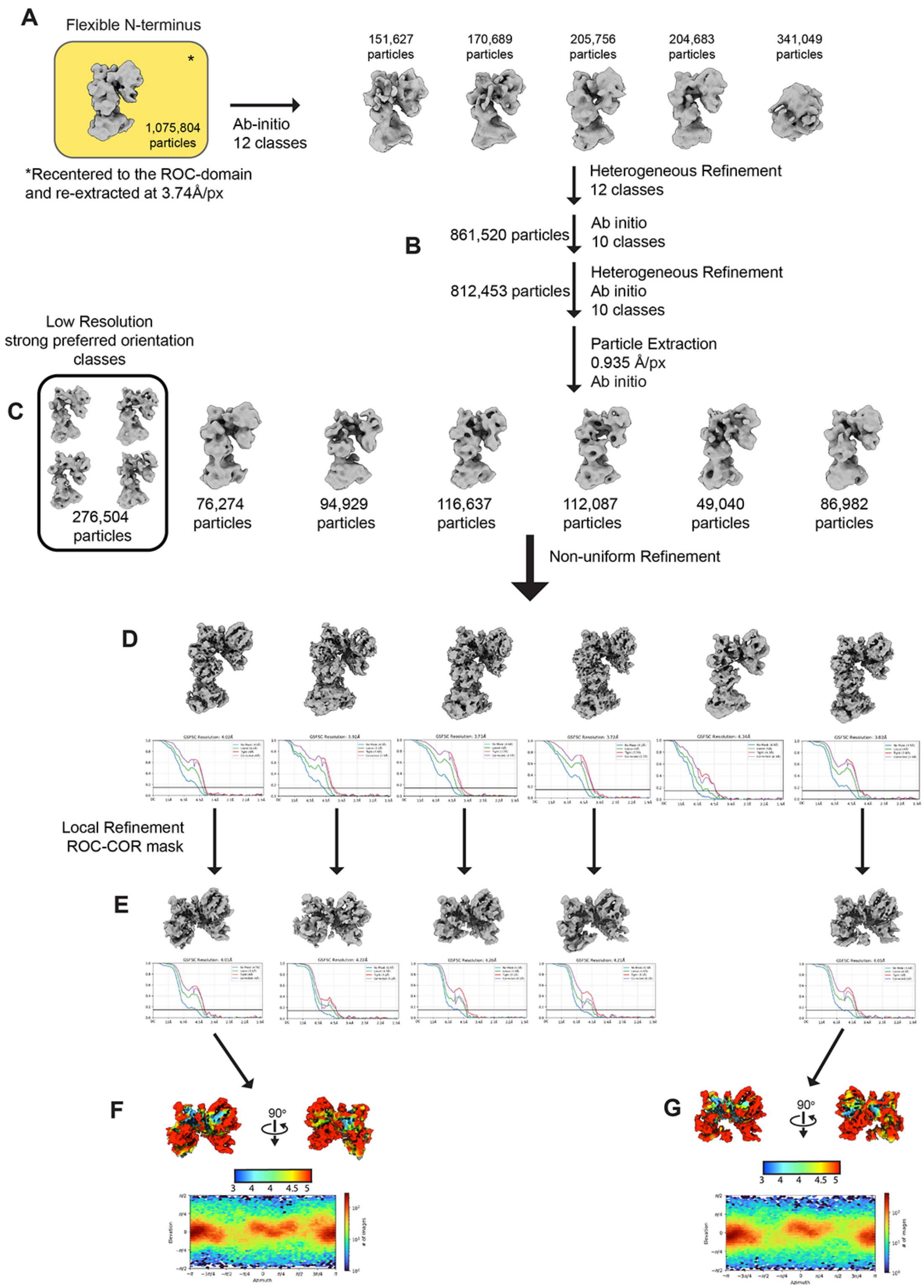

Figure S4. Cryo-EM processing pipeline for the combined data set: activated LRRK2 (C-terminal half), related to Figures 1 and 3. (A, B) Data processing strategy. **(C)** Splitting of activated volumes by conformation. **(D)** Volumes and FSC curves of activated LRRK2. **(E)** Local refinement volumes and FSC curves. **(F, G)** Local resolution maps and Euler angle distributions for activated LRRK2 (apo).

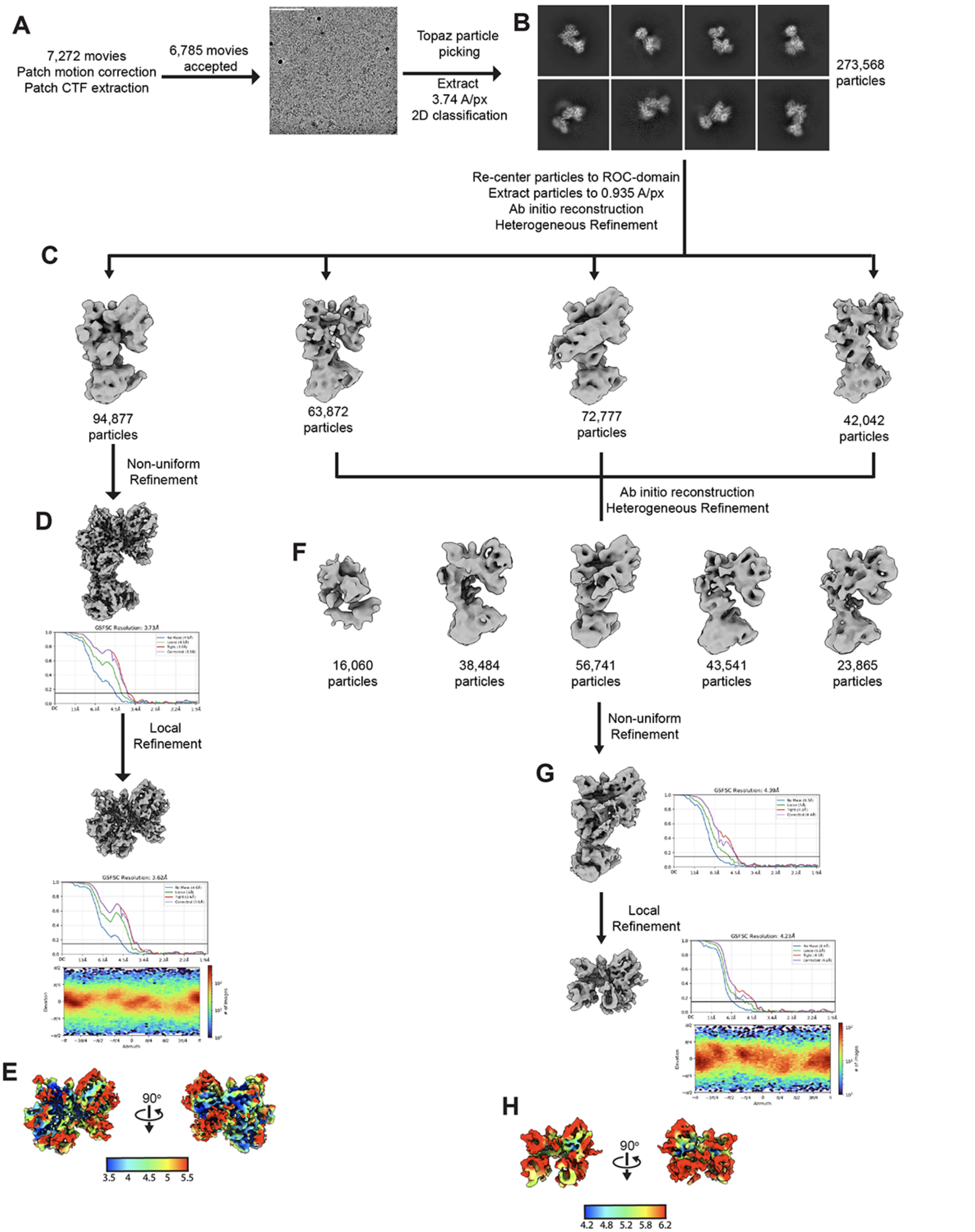

Figure S5. Cryo-EM data processing pipeline for LRRK2 incubated with GMPPNP for 24h, active and semi-active conformations, related to Figure 2H. (A) Number of movies collected and kept for further processing, and representative micrograph. **(B)** 2D class averages. **(C, F)** Data processing strategy. **(D)** Volume, FSC curve and Euler angle distribution, and **(E)** local resolution map of the ROC–COR domain of activated LRRK2. **(G)** Volume, FSC curve and Euler angle distribution. **(H)** Local resolution map of the ROC–COR domain of 'semi-activated' LRRK2.

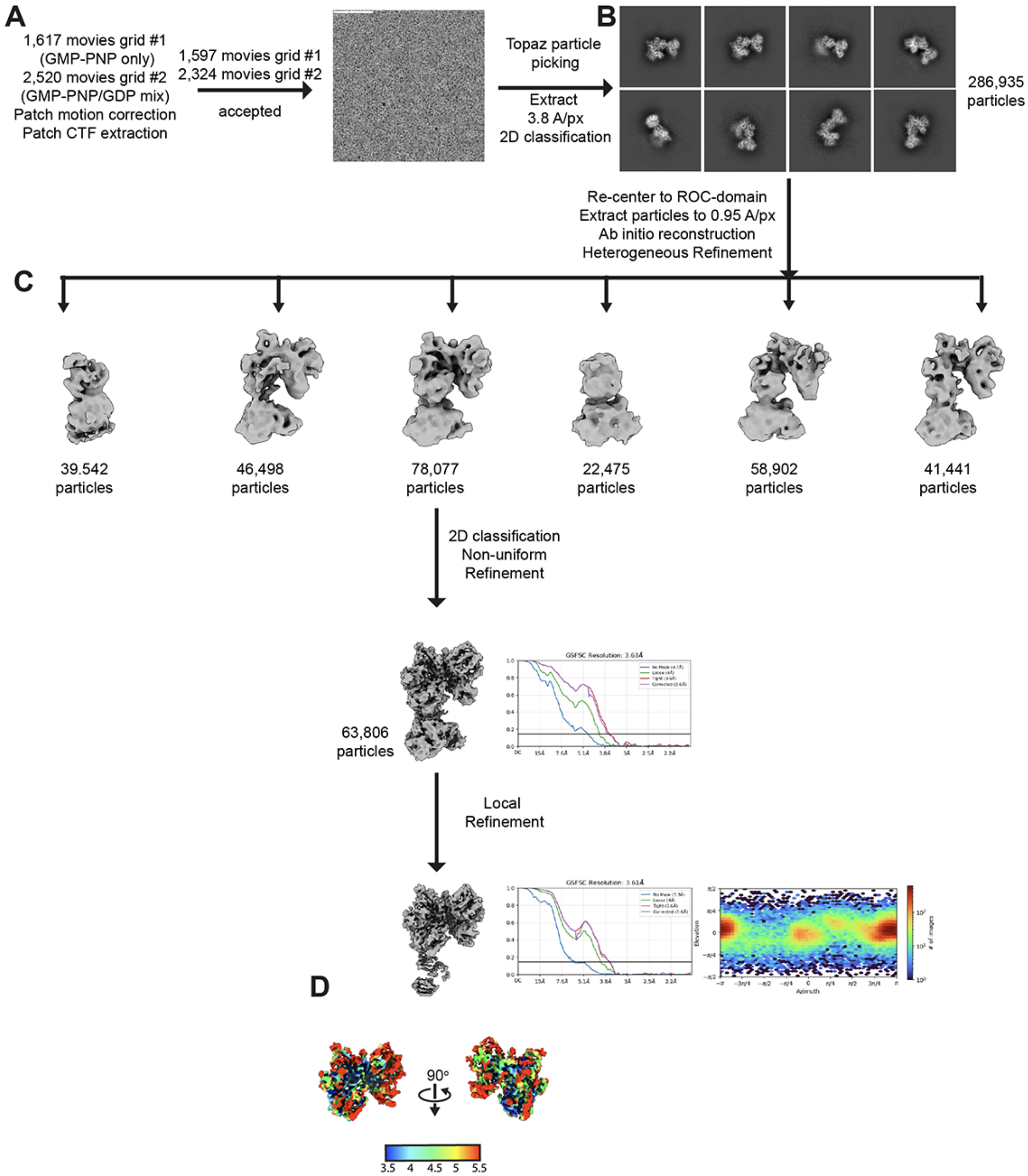

Figure S6. Cryo-EM processing pipeline for activated LRRK2[R1441C], related to Figures 1 and 3. (A) Number of movies collected and kept for further processing, and representative micrograph. **(B)** 2D class averages. **(C)** Data processing strategy. **(D)** Volume, FSC curve, Euler angle distribution, and local resolution map of the ROC–COR domain of activated LRRK2[R1441C].

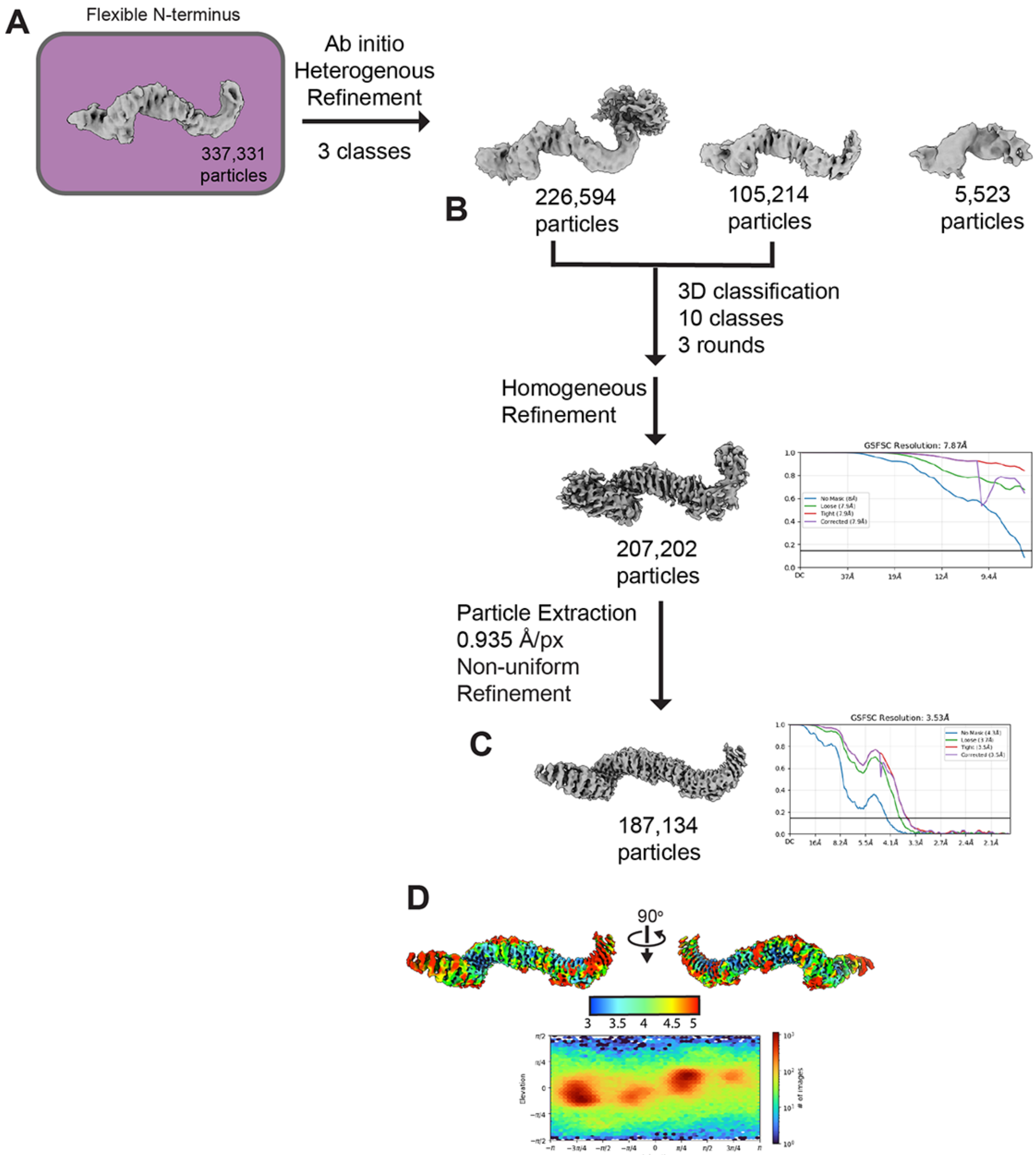

Figure S7. Cryo-EM data processing pipeline for the N-terminal repeats, related to Figures 1 and 3. (A, B) Data processing strategy. **(C)** Volume and FSC curve. **(D)** Local resolution map and Euler angle distribution.

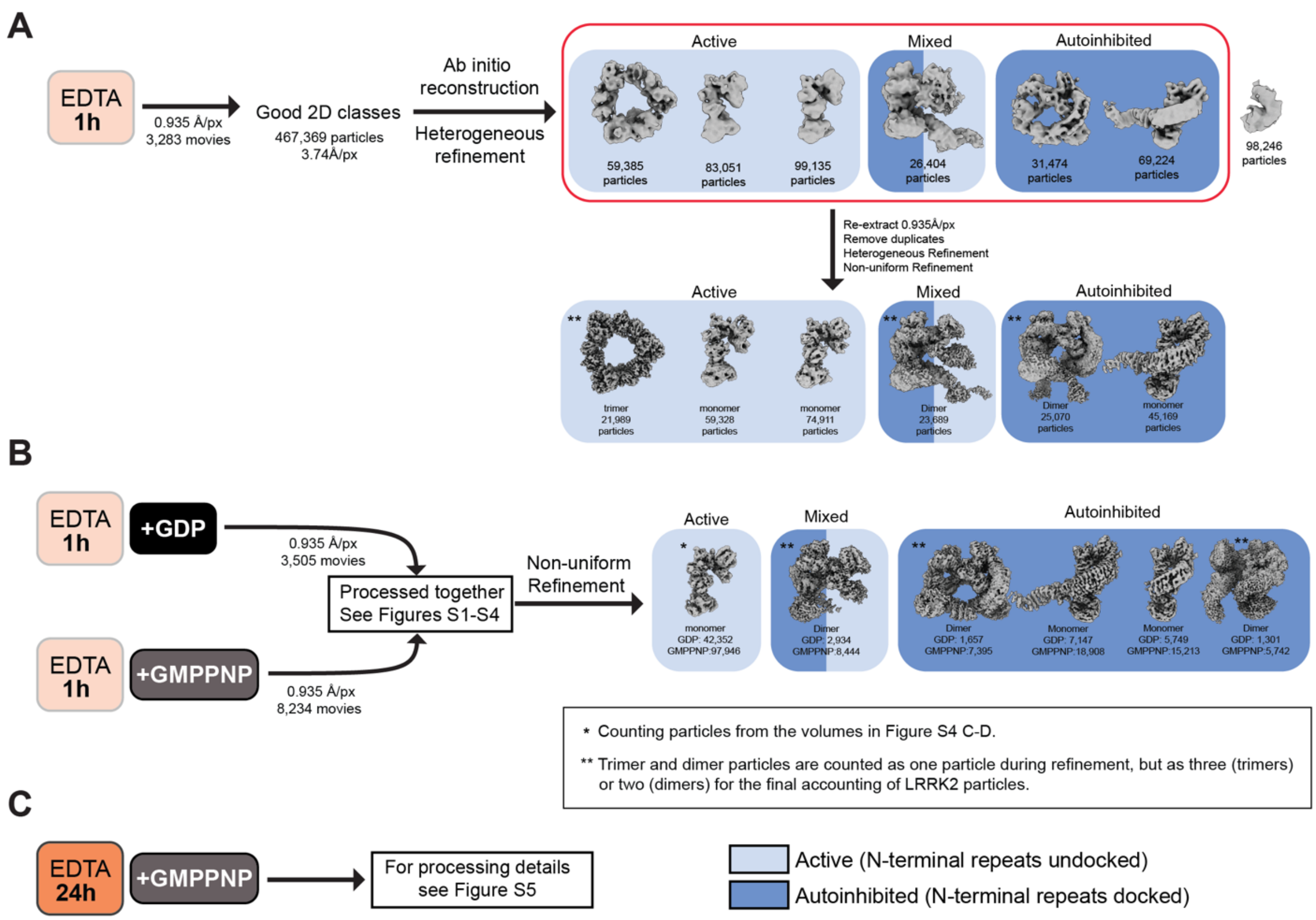

D

EDTA	1h	1h	1h	24h
**Nucleotide**	**none**	**GDP / 1h**	**GMPPNP / 1h**	**GMPPNP / 24h**
Active	223,895	45,291	230,482	257,508
Autoinhibited	118,998	21,751	68,839	0

EDTA	1h	1h	1h	24h
**Nucleotide**	**none**	**GDP / 1h**	**GMPPNP / 1h**	**GMPPNP / 24h**
Active	65%	68%	77%	100%
Autoinhibited	35%	32%	23%	0%

Figure S8. Distribution of autoinhibited and activated LRRK2 in samples that were treated with EDTA for 1h vs. 24h, related to Figure 2H. (A) Cryo-EM data processing of nucleotide-stripped LRRK2 (EDTA treatment only), yielding active LRRK2 in monomeric, trimeric, and mixed dimer forms, as well as autoinhibited LRRK2 in monomeric, dimeric, and mixed dimer forms. **(B)** Data classification for LRRK2 after nucleotide stripping and 1h incubation with GDP or GMPPNP, yielding active LRRK2 monomers and mixed dimers, and autoinhibited monomers, dimers, and mixed dimers. **(C)** Data classification for LRRK2 after nucleotide stripping for 24h and incubation with GMPPNP for 24h, yielding exclusively active LRRK2 monomers (see Figure S5 for data processing details). **(D)** Number and relative abundance (%) of particles corresponding to the active and inactive conformations of LRRK2 derived from cryo-EM datasets of LRRK2 subjected to EDTA treatment for 1h or 24h and then incubated with one of the following: no nucleotide, GDP, GMPPNP. The graph in Figure 2H corresponds to the relative abundances shown in the table.

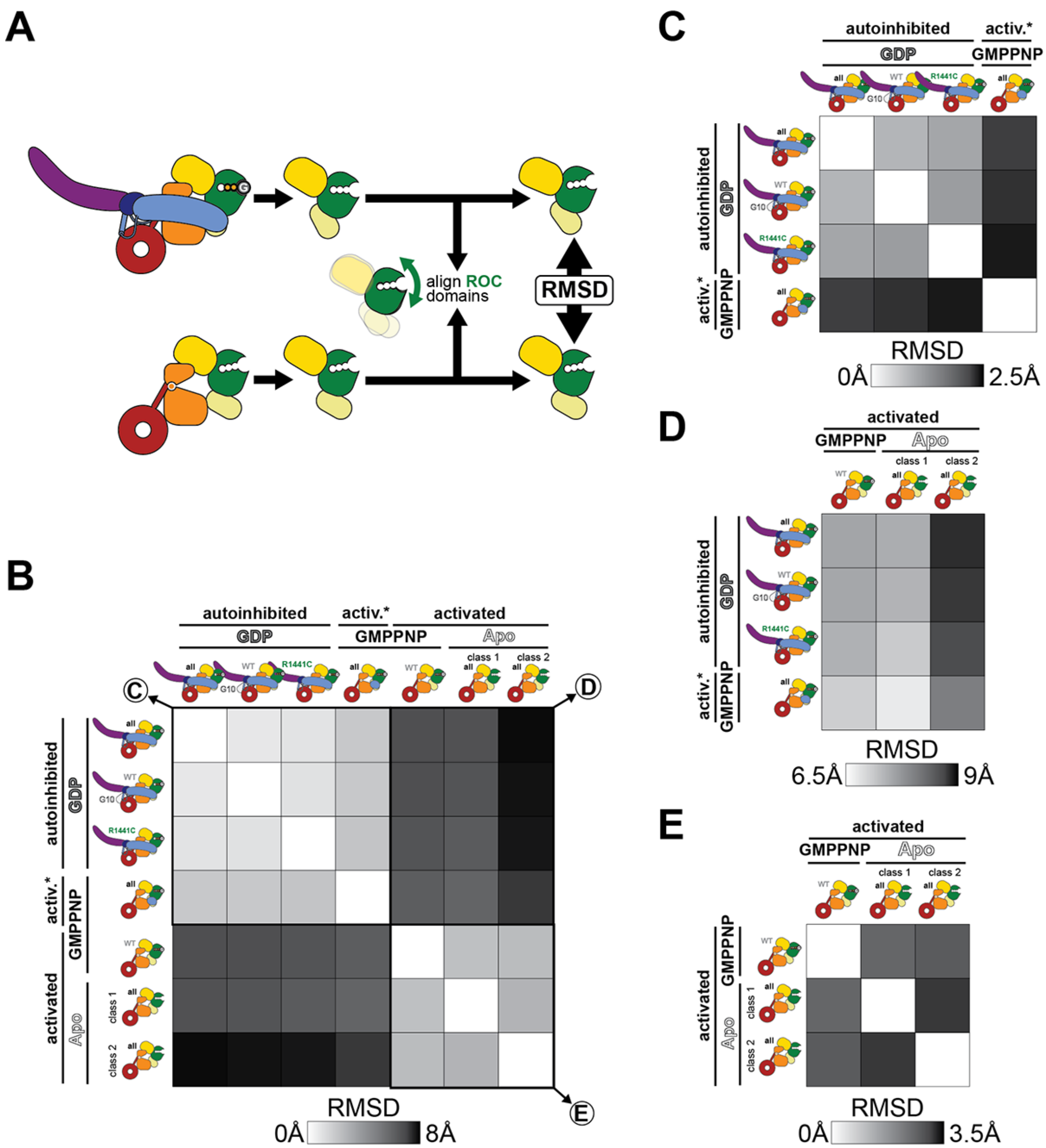

Figure S9. Quantification of conformational differences among structures of autoinhibited and activated LRRK2, related to Figure 3G-J. (A) Schematic representation of the approach used here to quantify conformation differences between structures. We focused our analysis on the ROC-COR module, where the largest conformational changes take place in the C-terminal half of LRRK2 ($LRRK2^{RCKW}$). For any pair of structures—in this example an autoinhibited LRRK2:GDP and an activated LRRK2:Apo—we extracted their ROC-COR modules and aligned them to each other by their ROC domains. We then measured the Root Mean Squared Deviation (RMSD) between the two ROC-COR modules (see Methods). Those values are shown in the matrices in greyscale. **(B)** Matrix of pairwise comparisons (RMSDs) for 7 structures: 3 of autoinhibited LRRK2, 3 of activated LRRK2, and the one structure of activated LRRK2 where we saw the LRR partially docked (labeled 'activated*' here). For all structures, we indicated the LRRK2 variant and the nucleotide state of the ROC domain. The RMSD scale used in this matrix is shown at the bottom. Three quadrants, which are replotted separately with different RMSD scales, are highlighted with thicker lines and their panels indicated. **(C-E)**, The 3 quadrants highlighted in (B) are shown with RMSD scales expanded to reveal differences among each subset of structures.

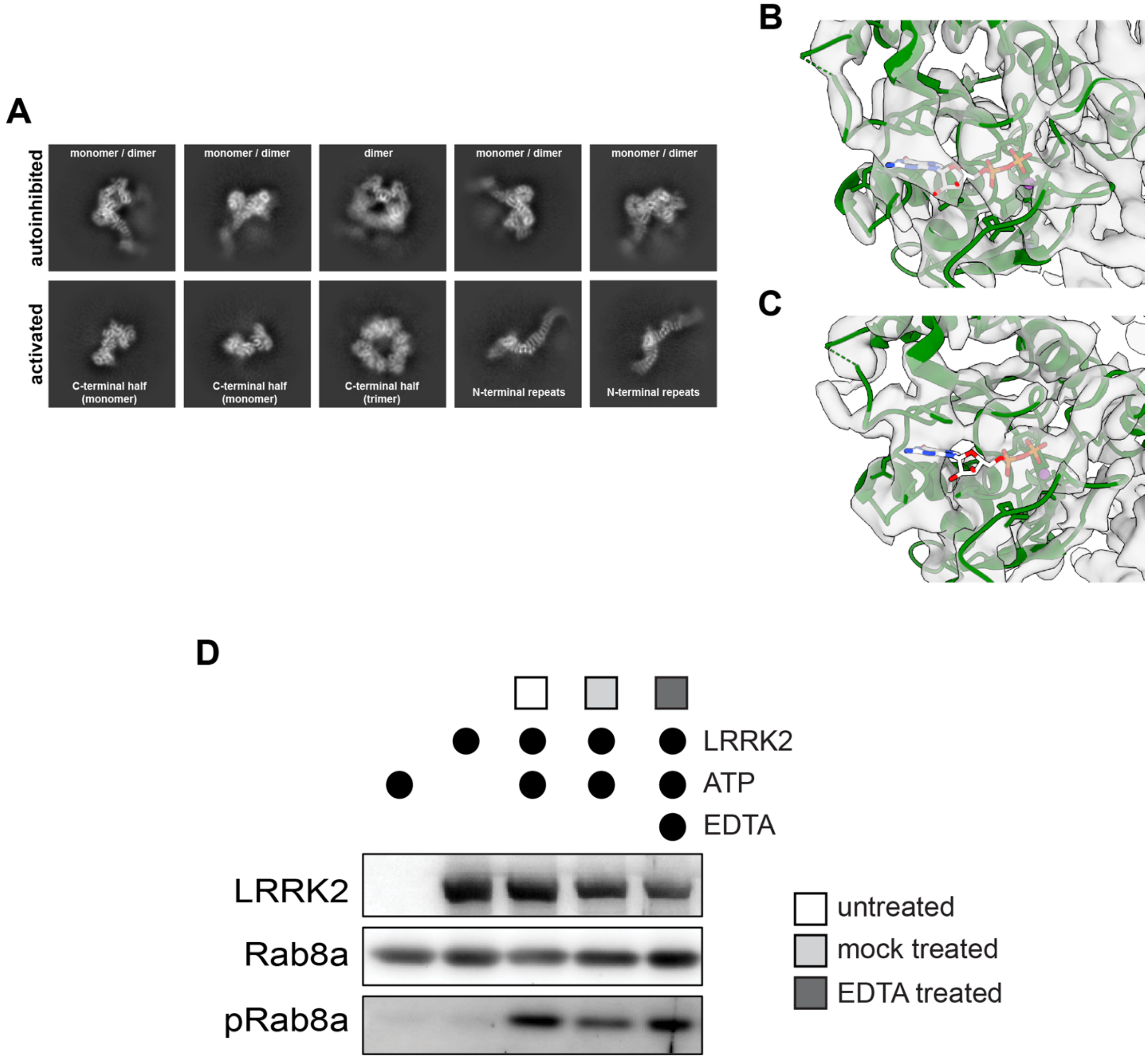

Figure S10. The nucleotide stripping protocol activates LRRK2, related to Figure 4A,B. (A) 2D class averages for LRRK2 that was subjected to the nucleotide-stripping protocol but to which no additional nucleotide was added. The top and bottom rows show species of autoinhibited (top) and activated (bottom) LRRK2. **(B, C)** Close-ups of the ROC domain for reconstructions obtained from the data shown in (A) for autoinhibited (B) and activated (C) LRRK2. The density in autoinhibited LRRK2 (B) can accommodate a nucleotide (GDP was modeled here based on the data discussed in the manuscript) while activated LRRK2 (C) cannot. **(D)** Representative immunoblot for in vitro Rab8a phosphorylation assays with or without treatment with EDTA (Figure 4B). ATP was omitted in one condition as a negative control.

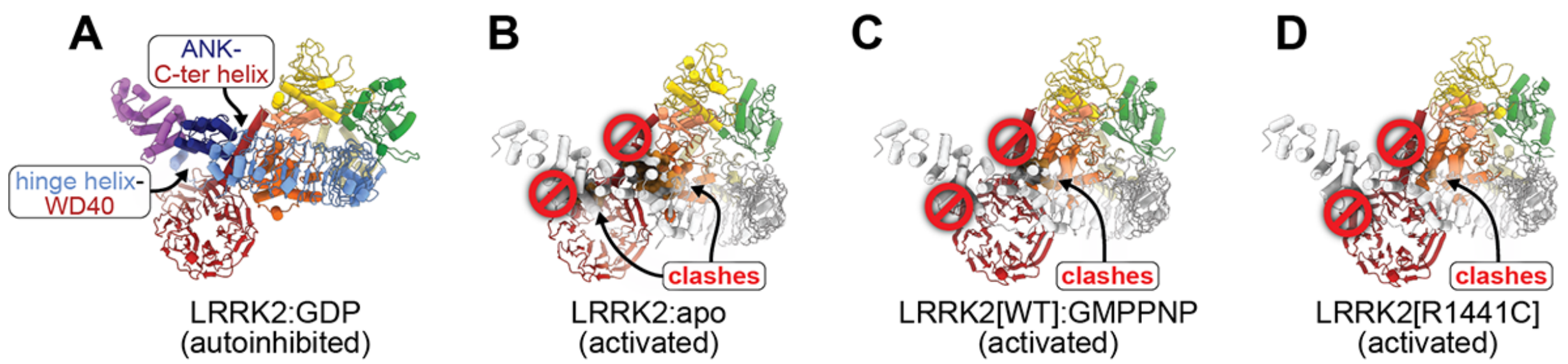

Figure S11. Modeling the redocking of the N-terminal repeats in the context of activated LRRK2, related to Figure 3L. **(A)** Structure of GDP-bound, autoinhibited LRRK2 highlighting the two main interactions that stabilize the docked conformation: one between the hinge helix in the LRR and the WD40 domain, and the other between the ANK domain and the C-terminal helix the follows the WD40. **(B-D)**, We modeled the N-terminal repeats into three of our structures of activated LRRK2: LRRK2:Apo (class 2) (Figure 3E), LRRK2[WT]:GMPPNP (Figure 3B), and LRRK2[R1441C] (Figure 3F). In all cases, we took a fragment comprising the N-terminal repeats and the ROC domain from autoinhibited LRRK2 and aligned that to each of the structures of activated LRRK2 via their ROC domains. These panels show that the interactions shown in (A) cannot form when the C-terminal half of LRRK2 is in its active conformation without additional flexibility in the N-terminal repeats, which we did not observe in our data. Furthermore, there are clashes between the repeats and the C-terminal half in all three models.

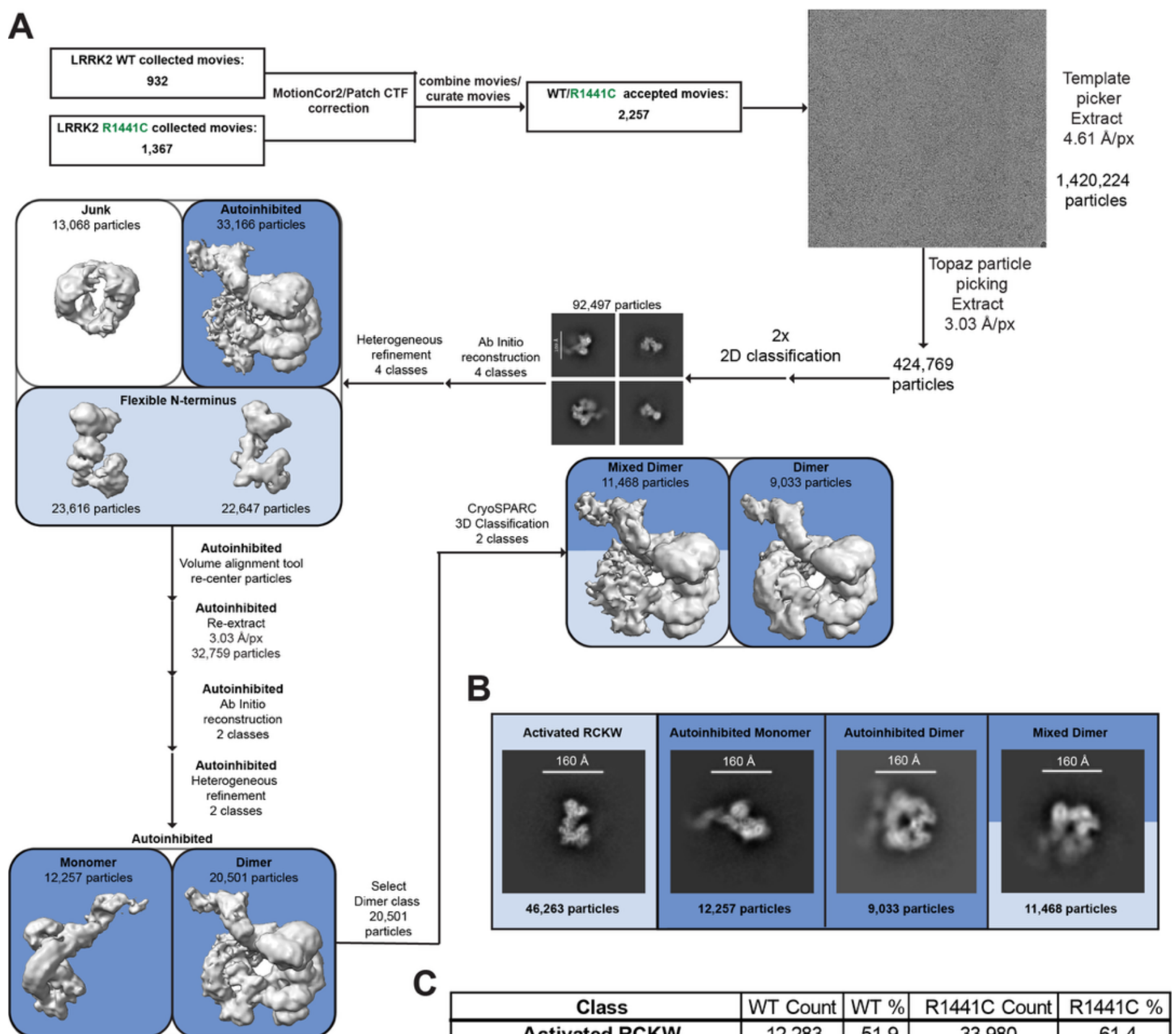

C

Class	WT Count	WT %	R1441C Count	R1441C %
Activated RCKW	12,283	51.9	33,980	61.4
Autoinhibited Monomer	5,090	21.5	7,167	13
Autoinhibited Dimer	3,077	13	5,956	10.8
Mixed Dimer	3,248	13.7	8,220	14.9

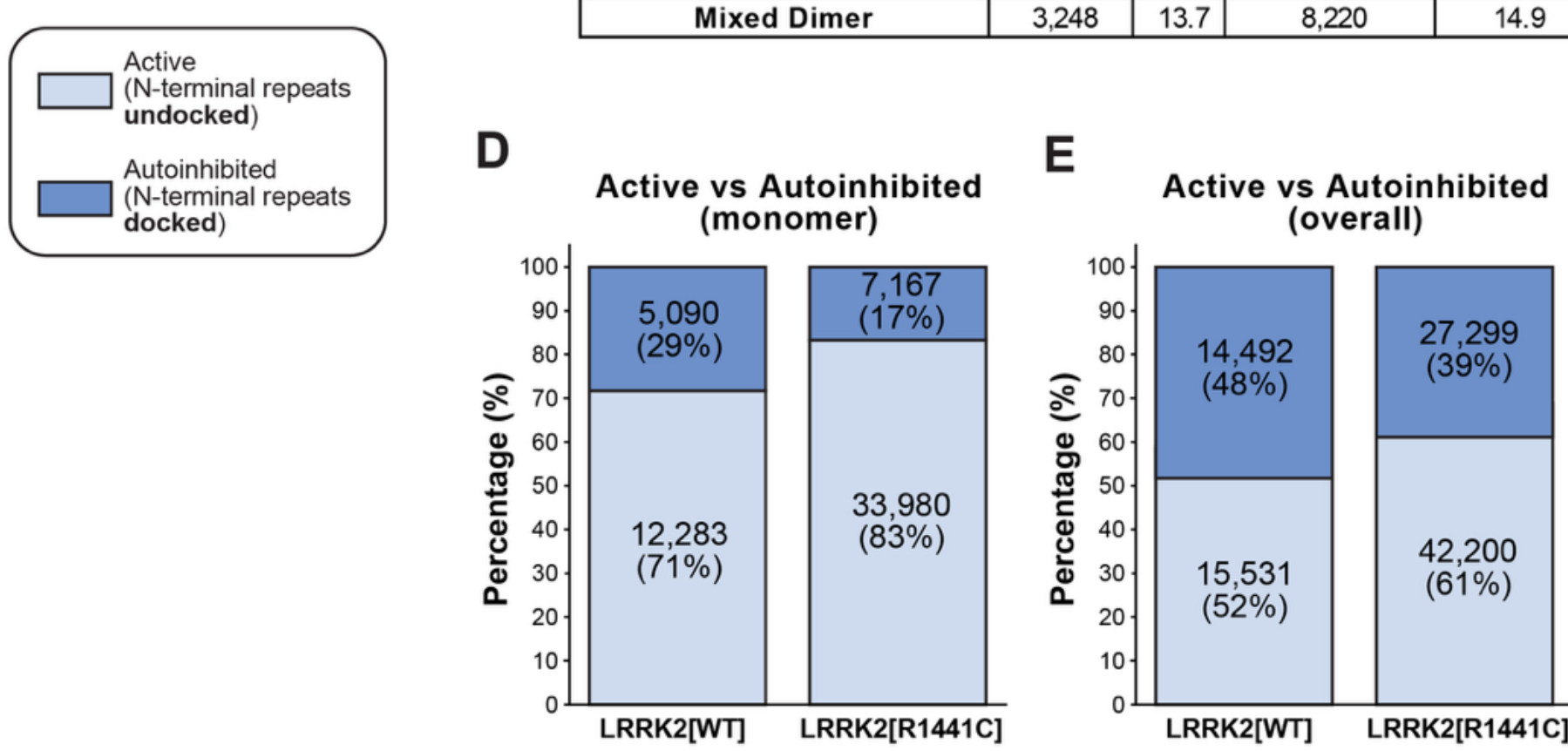

Figure S12. **The mutation R1441C increases the fraction of LRRK2 in the active conformation (N-terminal repeats undocked), related to Figure 3N-S. (A)** Data processing workflow for the combined LRRK2[WT] and LRRK2[R1441C] datasets. Light blue denotes active states and dark blue denotes autoinhibited states. **(B)** Representative 2D class averages for Activated LRRK2 ("RCKW"), Autoinhibited Monomer, Autoinhibited Dimer, and Mixed Dimer states derived from the combined dataset (79,021 total particles). **(C)** Particle counts and distributions across structural classes for LRRK2[WT] and LRRK2[R1441C] datasets. **(D)** Partition of active and autoinhibited states for monomeric LRRK2[WT] and LRRK2[R1441C]. **(E)** Partition of active and autoinhibited states for all species of LRRK2[WT] and LRRK2[R1441C]. Mixed dimers are counted twice, contributing one protomer to the active counts and one protomer to the autoinhibited counts.

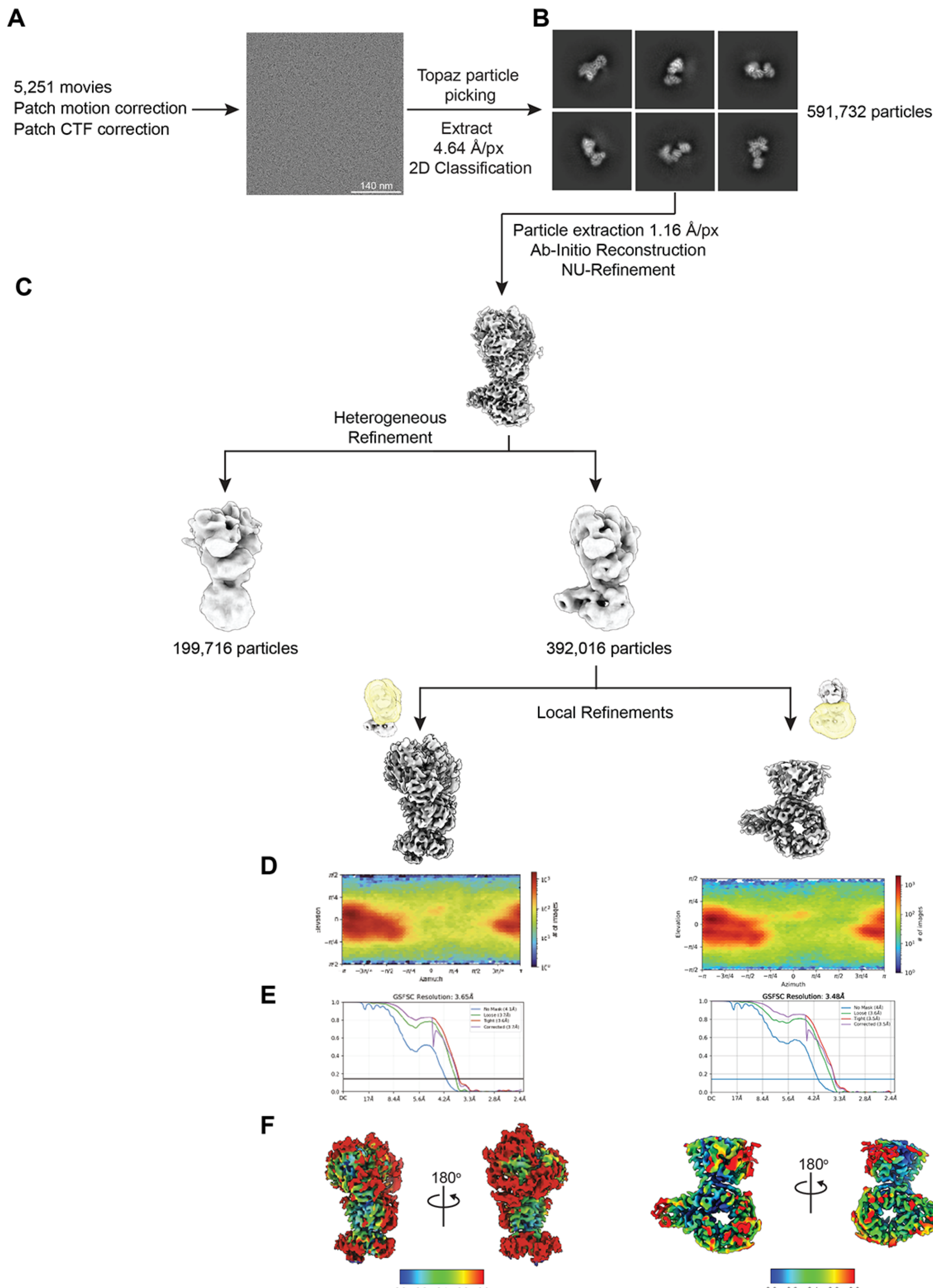

Figure S13. Cryo-EM data processing pipeline for LRRK2^{RCKW} bound to DARPin C12, related to Figure 4. (A) Number of movies collected and kept for further processing, and representative micrograph. **(B)** 2D class averages. **(C)** Data processing strategy. **(D, E)** Euler angle distribution (D) and FSC curve (E) for the volumes shown above. **(F)** Local resolution map.

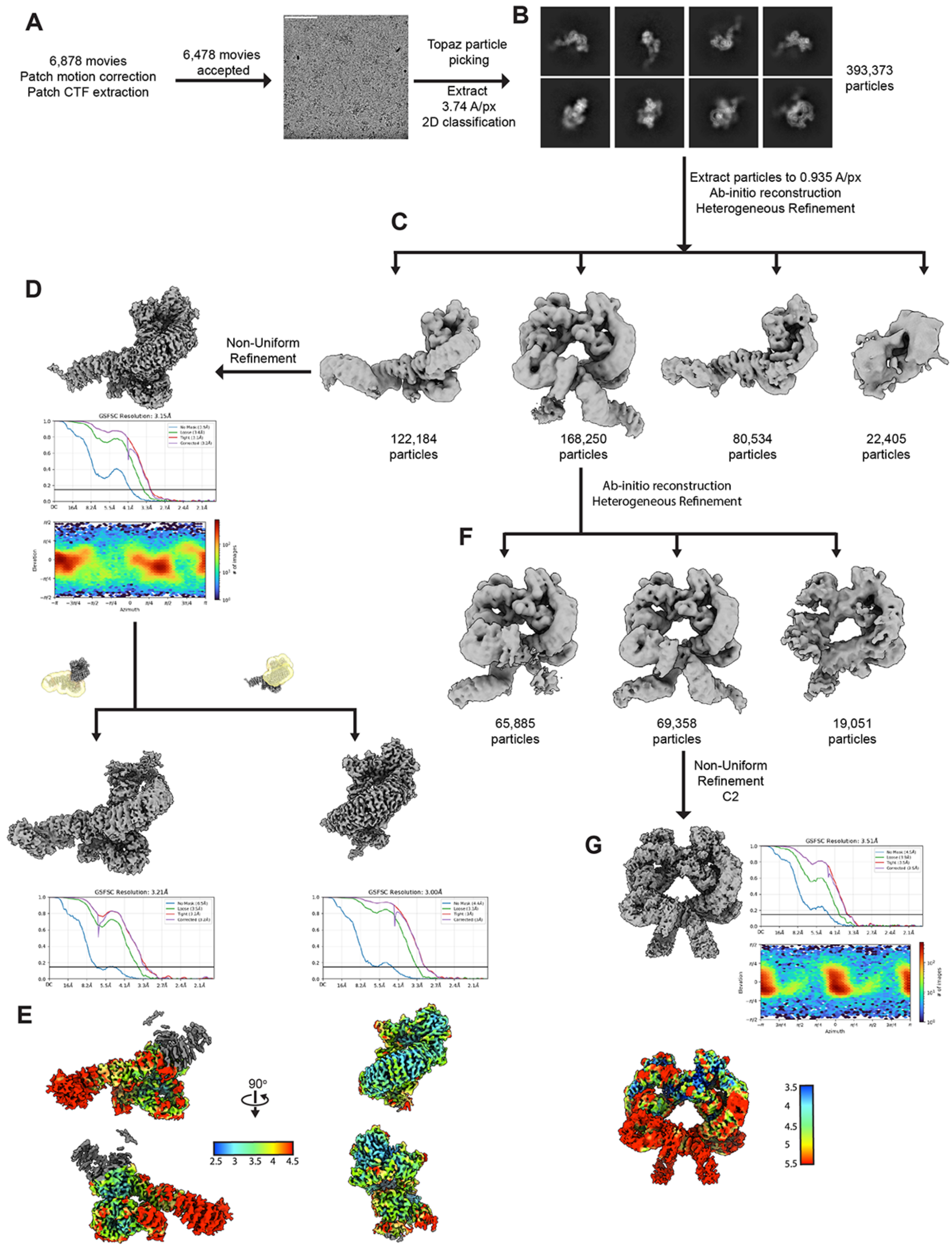

Figure S14. Cryo-EM data processing pipeline for LRRK2 bound to DARPin G10, related to Figure 4. (A) Number of movies collected and kept for further processing, and representative micrograph. **(B)** 2D class averages. **(C)** Data processing strategy. **(D)** Processing of monomeric LRRK2:G10, volume, Euler angle distribution and FSC curve. **(E)** Local resolution map of monomeric LRRK2:G10. **(F)** Processing of dimeric LRRK2:G10. **(G)** Volume, FSC curve, Euler angle distribution and local resolution map of dimeric LRRK2:G10.

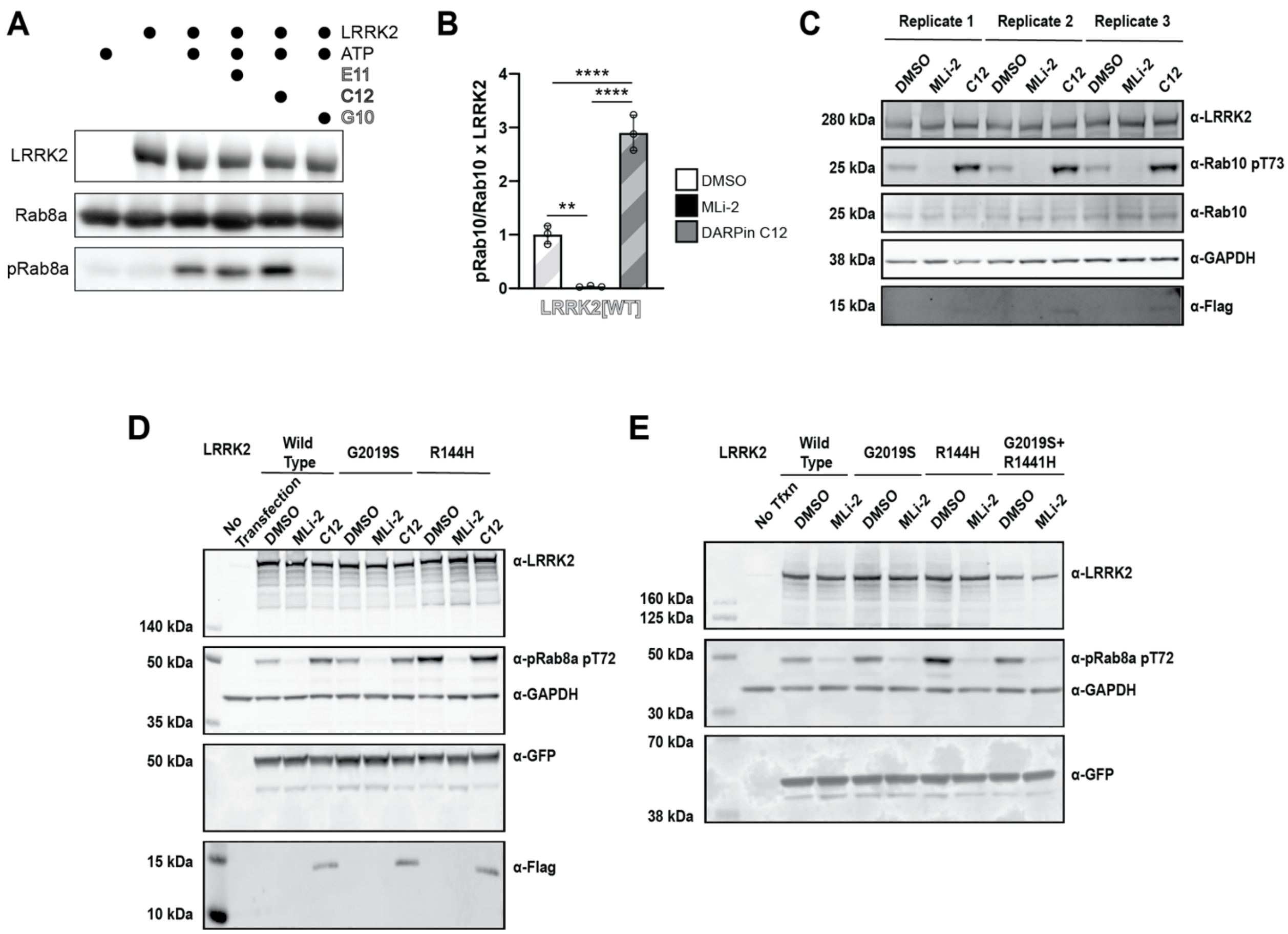

Figure S15. Representative immunoblots and phosphorylation of Rab10 in cells, related to Figures 5 and 6. (A) Representative immunoblot for Figure 5K (in vitro Rab8a phosphorylation assays with DARPins E11, C12, and G10). LRRK2[WT] was incubated with Rab8a in the presence of ATP and either no DARPin or one of the following DARPins: E11, C12, or G10. ATP was omitted in one condition as a negative control. **(B,C) Phosphorylation of Rab10 in cells.** Quantification **(B)** and immunoblot **(C)** of endogenous pRab10 (pT73) / Rab10 x LRRK2 normalized to the mean of LRRK2[WT] + DMSO condition**.** HEK 293Ts co-expressing LRRK2[WT] were treated with either DMSO or MLi-2 (5µM) for 1 hour. Cells were lysed and immunoblotted for LRRK2, Rab10, phosphor-Rab10 (T73), GAPDH, and Flag-tagged DARPin C12. All data were analyzed using a two-way ANOVA with Tukey's multiple comparison correction: DMSO vs MLi-2: **p=0.0036; MLi-2 vs DARPin C12: ****p<0.0001; DMSO vs DARPin C12: ****p<0.0001. Error bars ± SD**. (D,E) Representative immunoblots for Figure 6B,C** (effect of DARPin C12 on Rab8a phosphorylation in cells by LRRK2[WT], LRRK2[G2019S], and LRRK2[R1441H]) and Figure 6C (Rab8a phosphorylation in cells by LRRK2[G2019S], LRRK2[R1441H], and LRRK2[G2019S/R1441H]). **(D)** Representative immunoblot from 293T cells transiently transfected with GFP 11-tagged LRRK2[WT], LRRK2[R1441H], or LRRK2[G2019S] and its substrate GFP-Rab8a plus or minus Flag-tagged DARPin C12. LRRK2 and its mutants with GFP-Rab8a alone were treated with either DMSO or MLi-2 (5µM) for 1 hour. Lysed cells were immunoblotted for LRRK2, GFP-Rab8a, phospho-Rab8a (pT72), and GAPDH. Samples were immunoblotted for Flag separately. **(E)** Representative immunoblot from 293T cells transiently transfected with GFP 11-tagged LRRK2[WT], LRRK2[R1441H], LRRK2[G2019S], or LRRK2[G2019S/R1441H] and its substrate GFP-Rab8a and treated with either DMSO or MLi-2 (5µM) for 1 hour. Lysed cells were immunoblotted for LRRK2, GFP-Rab8a, phospho-Rab8a (pT72), and GAPDH.

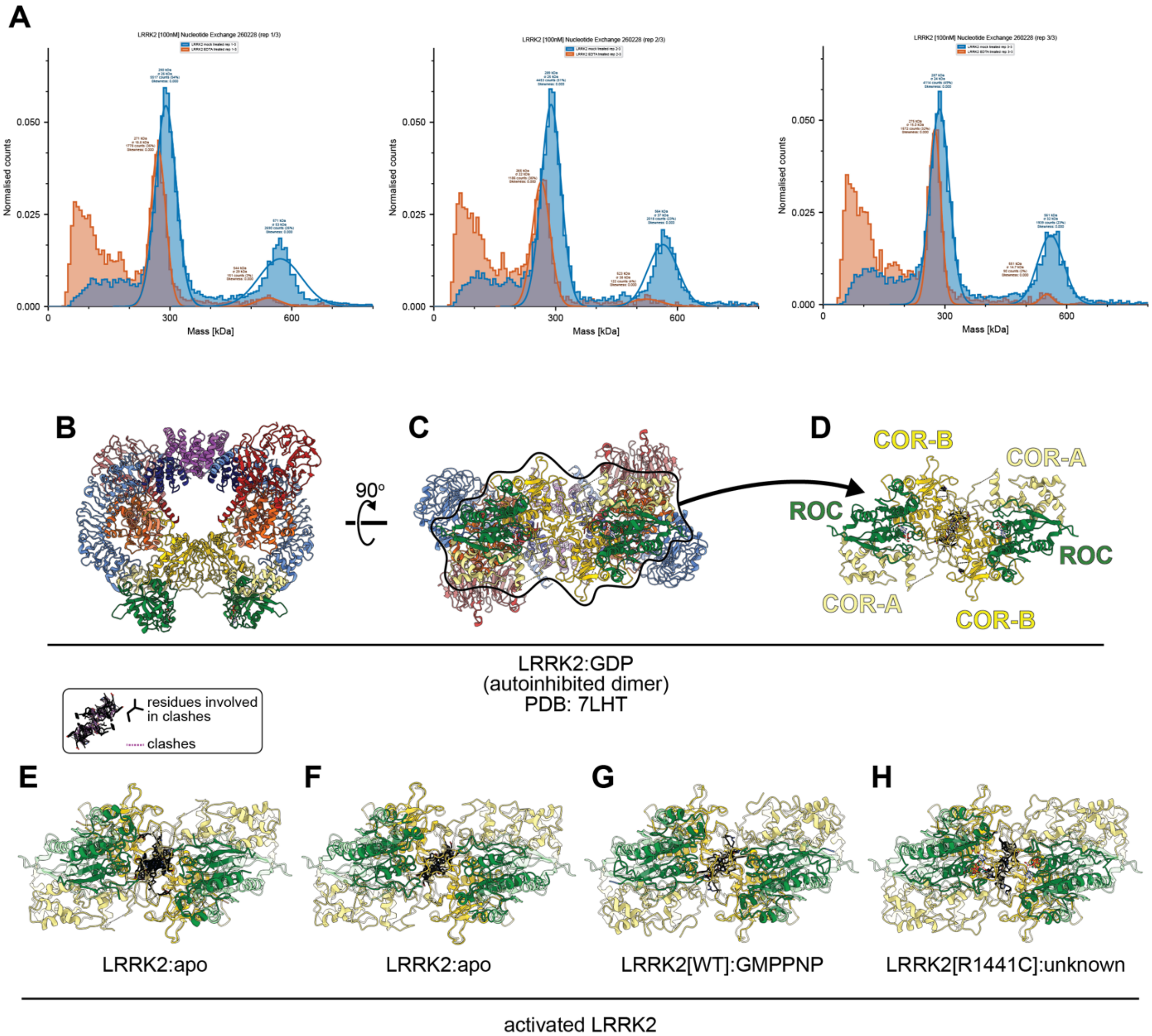

Figure S16. The active state of LRRK2 is likely to be a monomer, related to Figure 4. (A) Treatment of LRRK2 with EDTA shifts the population from the dimeric to the monomeric form. LRRK2 was either treated with EDTA for 2h (as in our nucleotide stripping protocol) or left untreated (mock treatment) (see Methods). The resulting samples were subjected to mass photometry to measure the abundance of LRRK2 monomers and dimers. The three technical replicates of the mass photometry measurements quantified in Figure 4D are shown here. **(B-H)** Modeling of activated LRRK2 in the context of the autoinhibited dimer. **(B,C)** Two views of the dimeric form of autoinhibited LRRK2:GDP (PDB: 7LHT). The view in (C) is from the dimeric interface, which involves the COR-B and COR-A domains. **(D)** Structure of the ROC-COR moieties of the LRRK2 dimer, showing the contacts (dashed black lines) involved in formation of the dimer. **(E-H)** Modeling of dimer interfaces for activated LRRK2. We took the ROC-COR moieties from four structures of activated LRRK2—LRRK2:Apo (1) (E), LRRK2:Apo (2) (F), LRRK2[WT]:GMPPNP (G), and LRRK2[R1441C] (nucleotide undetermined) (H)—and aligned them to the two COR-B domains that form the dimer interface in autoinhibited LRRK2. Residues involved in clashes are shown in black, and the clashes between them, as identified in ChimeraX, as purple dashed lines. Overlayed on these models is a semi- transparent version of panel (D) to highlight the conformational changes between the dimer interface in autoinhibited LRRK2 and the models shown here.

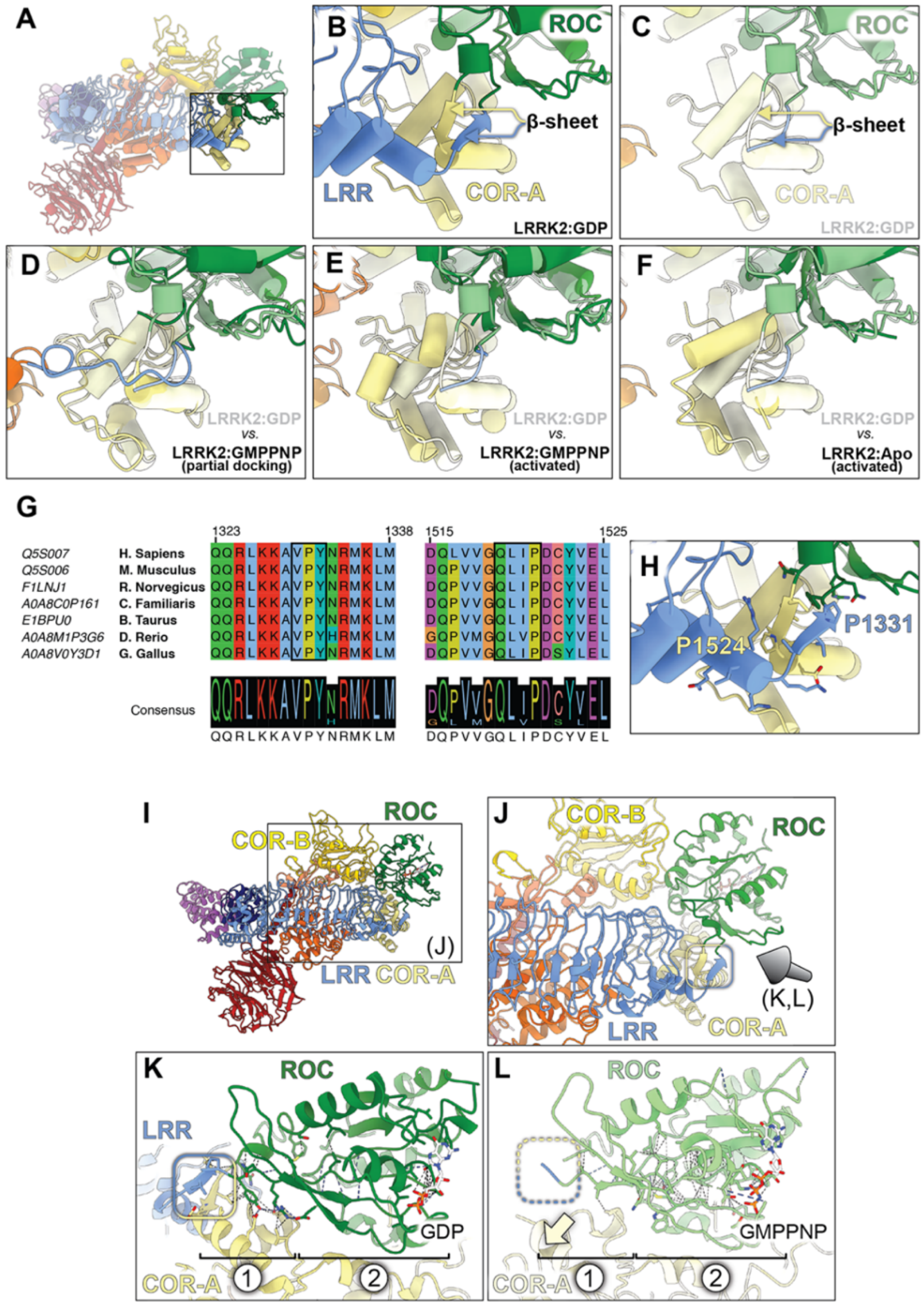

Figure S17. Model for nucleotide control of the docking and undocking of the N-terminal repeats. (A, B) In the autoinhibited (GDP-bound) form of LRRK2, the N-terminal repeats of LRRK2 connect to the GTPase (ROC) (A), via one strand of a two-stranded β-sheet (B). The other β-strand connects ROC to the COR-A domain (B). **(C)** Same panel as in (B), but with the N-terminal repeats of LRRK2 removed and the structure shown in lighter shades. This structure will be used as reference in panels (d-f) to illustrate the conformational changes between autoinhibited LRRK2 (in lighter shades) and different structures of activated LRRK2 (in the regular darker shades). **(D)** Comparison between autoinhibited LRRK2 and the structure of GMPPNP-bound LRRK2 (Figure 3C) with partial redocking of the LRR. Formation of the β-sheet is seen in this structure. **(E, F)** Comparison between autoinhibited LRRK2 and GMPPNP-bound (E) or Apo (F) activated LRRK2. The β-sheet is not formed in these structures. **(G)** Sequence alignment of the regions that encompass the two β-strands. **(H)** Close-up of the β-sheet and neighboring region, with the residues included in the alignment shown. The two prolines that mark the ends of the β-strands (P1331 and P1524) are labeled. **(I, J)** Structure of autoinhibited LRRK2, bound to GDP (I), with the region inside the square enlarged in (J). The blue/yellow rounded square in (J) shows the β-sheet connecting the LRR to the C-terminal half of LRRK2. The arrow indicates the viewing direction in panels (K) and (L). **(K, L)** Close-ups of the ROC domain and the region where it connects to the LRR and COR-A domains in the autoinhibited (GDP-bound) (K) and activated (GMPPNP-bound) (L) forms of LRRK2. The blue/yellow rounded square in (K) shows the β-sheet while the dotted version in (L) indicates its absence in the activated form of LRRK2. In addition to the presence or absence of this β-sheet, the two states differ in a set of interactions seen adjacent to the β-sheet when it is formed (region (1) in panel (K)), or close to the nucleotide in the activated (GMPPNP) form of LRRK2 (region (2) in panel (L)). The autoinhibited state of LRRK2 (K) shows several interactions in (1) and few in (2), while the reverse is seen in the activated state (L). The yellow arrow in (L) highlights the movement of the COR-A domain away from ROC responsible for breaking the β-sheet in activated LRRK2.

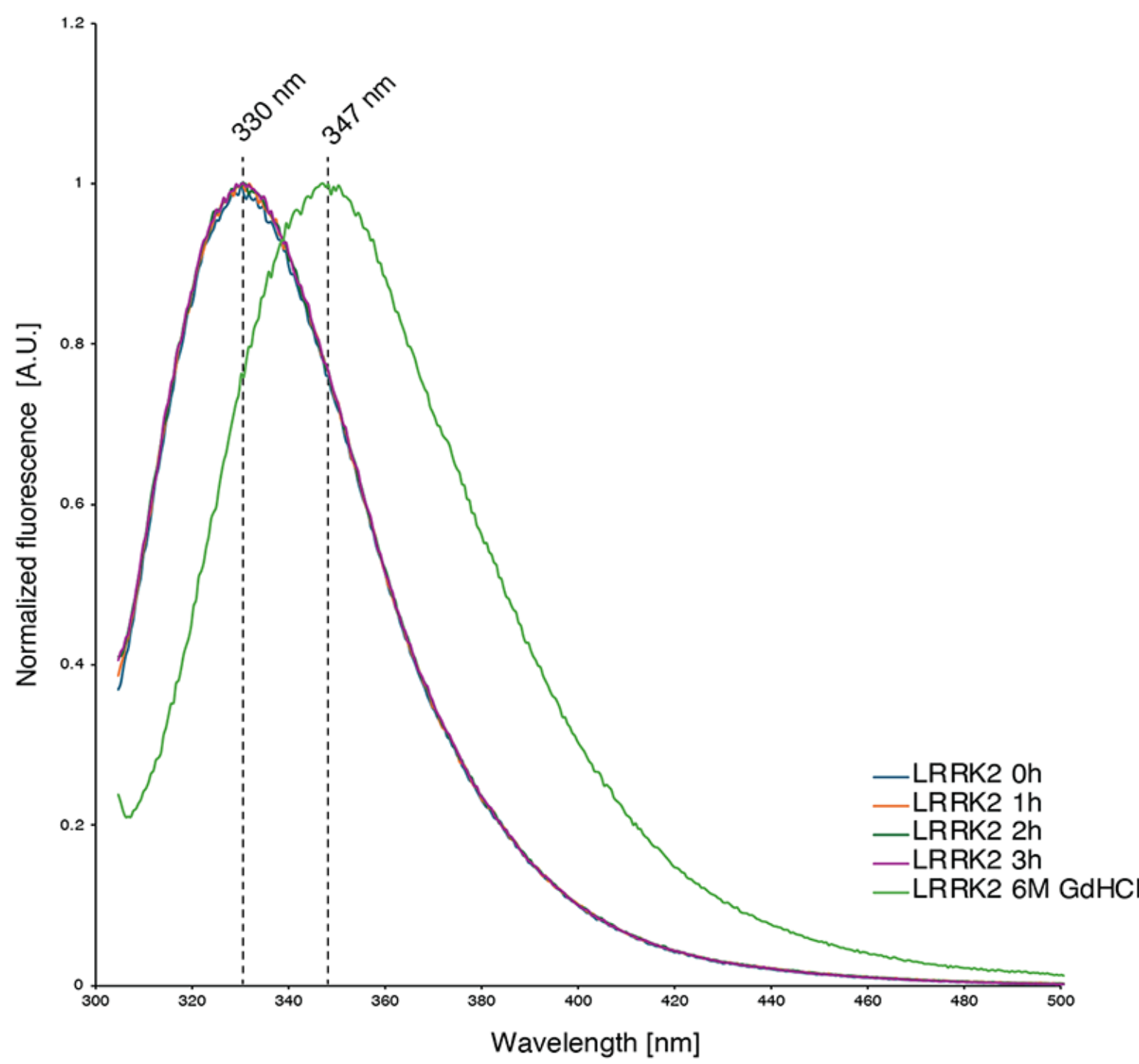

Figure S18. Fluorescence emission spectra for LRRK2 incubated at room temperature for different times. To determine the stability of LRRK2 incubated at room temperature for a few hours (needed for the nucleotide-exchange protocol), we measured fluorescence spectra for LRRK2[G2019S] incubated at room temperature at one-hour intervals from 0h (control) to 3h. We also measured the fluorescence spectrum of LRRK2 in 6M guanidinium hydrochloride as an unfolded control. LRRK2 was diluted to 1.56μM (or 40μM tryptophan).
